# Enantioselective Michael Addition of Cyclic β-Diones to α,β-Unsaturated Enones Catalyzed by Quinine-Based Organocatalysts

**DOI:** 10.3390/molecules22071096

**Published:** 2017-06-30

**Authors:** Qingqing Wang, Wei Wang, Ling Ye, Xuejun Yang, Xinying Li, Zhigang Zhao, Xuefeng Li

**Affiliations:** 1 College of Chemistry and Environment Protection Engineering, Southwest Minzu University, Chengdu 610041, China; wqqing767@163.com (Q.W.); m18328594286@163.com (W.W.); yangxuejun@swun.cn (X.Y.); lixinying1984@163.com (X.L.); zzg63129@163.com (Z.Z.); 2 Faculty of Geosciences and Environmental Engineering, Southwest Jiaotong University, Chengdu 610031, China; yeling_2004@126.com

**Keywords:** cyclic β-dione, cinnamone, chalcone, michael addition, enantioselective

## Abstract

An enantioselective (52–98% ee) Michael addition between cyclic β-diones and α,β-unsaturated enones was established in the presence of quinine-based primary amine or squaramide. A variety of cinnamones were smoothly converted into the desired 3,4-dihydropyrans in moderate to high yields (63–99%). Chalcones were also suitable acceptors and gave rise to the expected adducts in satisfactory yields (31–99%). The resulting adducts readily underwent further modification to form fused 4*H*-pyran or 2,3-dihydrofuran.

## 1. Introduction

The Michael addition of α,β-unsaturated compounds is an atom-economic carbon-carbon bond-forming reaction in organic synthesis, and the development of the enantioselective catalytic approach for this transformation, has attracted intensive attention [1,2,3,4]. Among the often-used acceptors, unactivated α,β-unsaturated enones always exhibit relatively sluggish reactivity and emerge as a class of historically challenging substrates for metal- and organocatalytic approaches [5,6,7,8,9,10,11]. In this context, the elegantly-designed chiral primary amines, especially those based on cinchona alkaloids, provide a particularly efficient LUMO-lowering (LUMO: lowest unoccupied molecular orbital) activation mode through the formation of iminium ions with these unsaturated enones [12,13,14]. Therefore, a broad range of conjugate additions of α,β-unsaturated enones with various different nucleophiles have been successfully established with constantly high enantiocontrol [15,16,17,18,19]. However, the asymmetric Michael addition of cyclic 1,3-dicarbonyl compounds [20,21,22,23,24,25,26,27], except for 4-hydroxycoumarin and its analogues [7,28,29,30,31,32,33,34,35,36], to α,β-unsaturated enones, especially chalcones, generally draws less attention in comparison with other type of donors [37,38,39], albeit the adducts of such a conjugate addition reaction are versatile precursors to construct several classes of compounds possessing enormous bioactivities [40,41]. Liu and Feng have successfully developed an efficient Michael addition between dimedone and cinnamones employing the unmodified chiral diphenylethylenediamine (DPEN) [37]. In contrast, only moderate enantioselectivity was obtained for the Michael addition of dimedone to unfunctionalized chalcone according to Singh’s protocol [38]. Consequently, the development of the enantioselective Michael addition of cyclic β-diones to α,β-unsaturated enone is still highly sought.

Based on our continuous interest in asymmetric Michael reactions involving α,β-unsaturated enones [42,43,44,45], herein we would like to further extend the scope of donor to cyclic β-diones [20,21,22,23,46,47,48,49]. Dimedone and its analogues smoothly react with a variety of α,β-unsaturated enones, furnishing the corresponding adducts in good yields and high levels of optical purities. The synthetic potential of the desired Michael adduct is demonstrated by the easy formation of enantioenriched 4*H*-pyran and 2,3-dihydrofuran.

## 2. Results and Discussion

**Figure 1:**
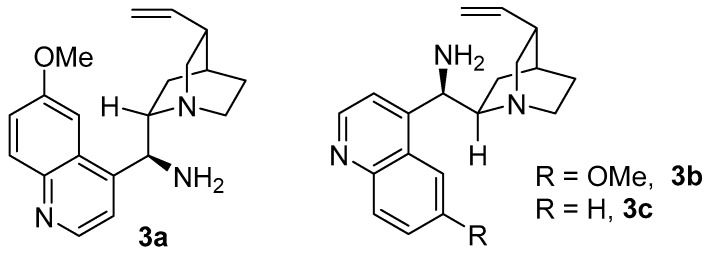
Structures of the chiral primary amine catalysts used.

We were pleased to find that the Michael addition of dimedone **1a** to cinnamone **2a** proceeded smoothly in the presence of 9-amino(9-deoxy)-epi-quinine **3a** (Figure 1) in combination with a series of different acid co-catalysts. It was documented that the acid co-catalyst had a great influence on the yield and enantioinduction [16]. The aromatic carboxylic acids displayed superior catalytic effect compared with sulfonic acid and aliphatic acids (Table 1, entries 4–9 vs. entries 1–3). The desired 3,4-dihydropyran **4a** was generated with good to excellent enantioselectivities (87–90% ee) in the presence of various aromatic acids. In contrast, salicylic acid (SA) afforded an optimal yield (99%) and a superior enantioselectivity (90% ee) (entry 9 vs. entries 4–8) [50]. Having identified salicylic acid as the preferential acid co-catalyst, we turned our attention to evaluate the effect of other primary amines **3b** and **3c** (Figure 1) derived from naturally occurring cinchona alkaloids [51]. Both **3b** and **3c** delivered the expected 3,4-dihydropyran **4a** possessing opposite configurations to the adduct afforded by **3a** (entries 10 and 11). Moreover, these two pseudo-enantiomers displayed poorer catalytic activities and enantioselectivities compared with 9-amino(9-deoxy)-epi-quinine **3a** (entries 10 and 11 vs. entry 9). Subsequently, we examined the effect of the solvent with a combination of **3a** and salicylic acid. Tetrahydrofuran (THF) emerged as the favorable one in terms of reactivity and enantioselectivity (entry 15 vs. entries 9, 12–14). Notably, the model process proceeded equally smoothly when the amount of cinnamone was decreased to 1.2 equivalents (entry 16 vs. entry 15). Reducing the reaction temperature (0 °C) led to a slightly higher enantioselectivity (97% ee) (entry 17).

With the optimal reaction conditions in hand, various cinnamones **2** were treated with dimedone **1a** to determine the scope and generality of this Michael addition. As presented in Table 2, the electronic property exerted marginal impact on this asymmetric process. The electron-deficient cinnamones **2c**–**2f** generally provided the corresponding 3,4-dihydropyrans in slightly higher chemical yields, in contrast with the electron-rich acceptors **2g** and **2h** (Table 2, entries 3–6 vs. entries 7 and 8). Meanwhile, all these enones gave rise to the desired adducts with excellent enantioselectivities (96–97% ee) irrespective of electronic nature (entries 3–8). On the other hand, the steric hindrance slightly impaired the reactivity of this conjugate addition reaction. In this context, the *ortho*-substituted enone **2b** afforded somewhat poorer conversion (87% yield) in comparison with other electron-poor cinnamones **2c**–**2f** (96–99% yield) (entry 2 vs. entries 3–6). Gratifyingly, **2i** and **2j**, both possessing a bulky naphthyl group at the β-site, were also compatible with this catalytic system (entries 9 and 10). The resulting adducts **4ai** and **4aj** were formed in excellent yields and with high levels of enantioselectivities. The heteroaromatic enones **2k** and **2l** were all suitable partners for this Michael reaction (entries 11 and 12). The alkyl-substituted enones **2m** and **2n** were found to react relatively slowly with dimedone, however, synthetically useful yields and satisfactory enantiocontrol were still obtained (entries 13 and 14). Remarkably, cyclic enone **2o** was also a competent acceptor, furnishing the bridged-ring compound **4ao** in 91% yield and 98% ee (entry 15) [37]. The ketone substituent (R_2_) could also be varied from methyl group to ethyl group. Although relatively poorer conversion was detected, excellent enantioselectivity was maintained for this sterically more hindered acceptor (entry 16). It seemed to be an effect of increased steric bulk on the ketone, retarding the acceptor to approach the catalyst, thereby slowing down the reaction rate. On the other hand, the unsubstituted cyclic β-dione, 1,3-cyclohexanedione, was also tolerated by this catalytic system. Acceptable yields (67–78%) and high degrees of enantiomeric excesses (94–96% ee) were successfully achieved (entries 17–19), despite its relatively lower reactivity in contrast with dimedone [20,52]. Notably, a one mmole-scale Michael addition of cinnamone **2a** and dimedone **1a** was performed under optimal reaction conditions. Excellent chemical yield (95%) and enantiopurity (94% ee) were both obtained (entry 1).

**Scheme 1:**
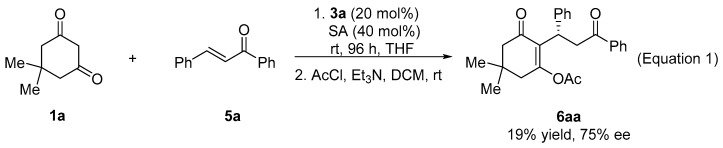
Michael addition of dimedone to chalcone.

Having identified cinnamones as the suitable acceptors, we successively turned our attention to chalcone (Scheme 1), a class of challenging substrates for iminium ion activation [16]. Different than cinnamones, the bulky benzene group might retard the later annulation process, therefore only the initial Michael adduct was accessed. Considering the unstability of the Michael adduct due to aerobic oxidation [53], a subsequent acetylation was conducted after the initial conjugate addition in a one-pot manner. To our disappointment, the titled process allowed access to the final acetyl derivative **6aa** in fairly low yield (<20%), even when the initial Michael addition was performed at room temperature.

Fortunately, we finally found that the Michael addition of chalcone worked properly in the presence of squaramide **7** derived from quinine (see supporting material) [54,55]. As outlined in Table 3, this Michael addition was independent of the electronic nature of the substituents on the aromatic rings. Both the electron-rich acceptors **5b** and **5f** and the electron-deficient acceptors **5c** and **5g** generated the expected adducts in satisfactory yields and excellent optical purities (Table 3, entries 2 and 6 vs. entries 3 and 7). Moreover, the steric hindrance exerted influence on this Michael addition to a certain extent. The enone **5e** possessing a naphthyl group afforded relatively lower isolated yield (68%) even after a prolonged reaction time, albeit accompanied by outstanding enantioselectivity (entry 5). Heteroaromatic chalcones **5d** and **5h** were also favorable partners, giving rise to the final acetyl derivatives with high levels of enantiopurities (entries 4 and 8). Except for dimedone, 1,3-cyclohexanedione **1b** was a competent donor as well, albeit a longer reaction time was required in order to achieve complete conversion (entry 9). In contrast with Singh’s precedent study (72% ee for **6aa**) [38], our protocol efficiently improved the enantioselectivity and displayed a wide substrate generality for this Michael addition of cyclic β-dione to chalcone [56]. Moreover, a one mmole-scale Michael addition of chalcone **5a** with dimedone **1a** proceeded smoothly as well. The expected acetyl derivate **6aa** was formed in an almost quantitative yield and with satisfactory enantioselectivity (entry 1). The alkyl-substituted enone **5i** was also a suitable acceptor, albeit unsatisfactory enantioselectivity was obtained for the resulting Michael adduct (entry 10).

**Scheme 2:**
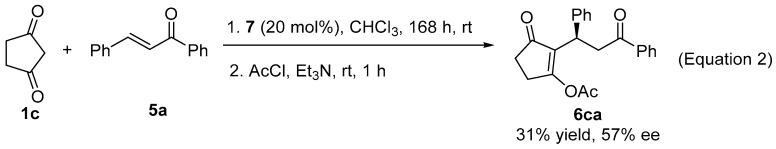
Michael addition of 1,3-cyclopentadione to chalcone.

The five-membered cyclic dione, 1,3-cyclopentadione **1c**, was also tolerated by our catalytic protocol (Scheme 2). However, it proved to be an inferior donor in terms of reactivity and enantioselectivity, in contrast with the six-membered cyclic dione. The related acetyl derivative **6ca** was obtained in an unsatisfactory yield and with moderate optical purity.

**Scheme 3:**
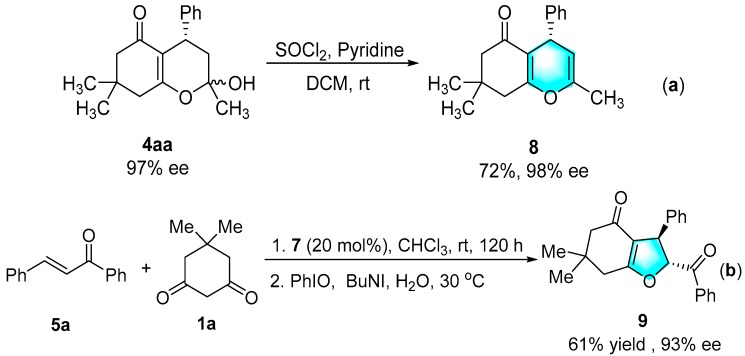
Synthetic elaborations of the Michael adducts.

To demonstrate the synthetic potential of this Michael reaction, product modification was performed on the Michael adducts. 3,4-Dihydropyran **4aa** readily underwent a dehydrating procedure to afford 4*H*-pyran **8** without the loss of optical purity (Scheme 3a) [20]. The Michael adduct of chalcone could be utilized for the facile preparation of the biologically interesting 2,3-dihydrofuran **9** via a successive stereoselective oxidative cyclization process (Scheme 3b) [57]. The fused 2,3-dihydrofuran **9** was obtained as a single *trans*-diastereomer in a synthetically useful yield and with excellent enantioselectivity.

**Scheme 4:**
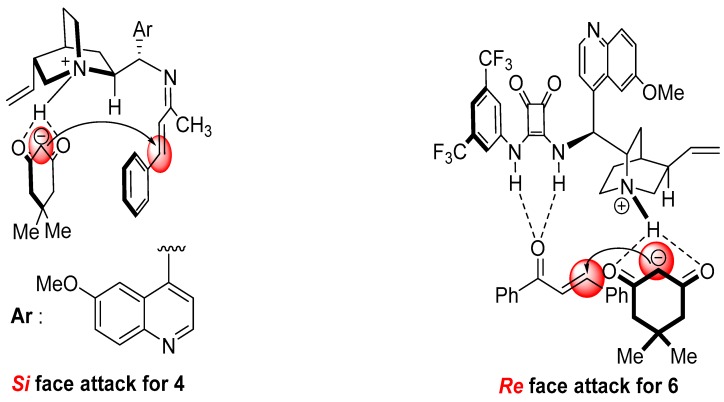
Proposed transition state models.

The absolute configuration of the Michael adduct **4ad** (Table 2, entry 4) was determined to be *S* via comparison of the optical rotation value and HPLC traces with that of the previous literature reports [37]. On the other hand, the absolute configuration of **6aa** (Table 3, entry 1) was established as *R* by the analysis of the optical rotation value with Singh’s protocol [38]. To account for the observed stereochemical outcome of these Michael reactions, the corresponding transition state models were proposed and described in Scheme 4. The primary amine motif of 9-amino(9-deoxy)-*epi*-quinine **3a** was engaged in iminium formation with the carbonyl group of benzalacetone **1a**. Meanwhile, dimedone was deprotonated by the tertiary amine moiety of aminocatalyst **3a** and orientated via hydrogen-bonding, thereby leading to a favorable attack toward the *si*-face of cinnamon **1a**. As a result, the desired *S*-configured product **4a** was obtained. On the other hand, chalcone **5a** was efficiently activated via hydrogen-bonding interactions between the NH moiety of the squaramide **7** and the carbonyl group of chalcone. Furthermore, the *re*-face approach of dimedone was induced by the tertiary amine of the squaramide **7** and led to the formation of the major stereoisomer with the *R* configuration [58]. 

## 3. Materials and Methods

### 3.1. General Remarks

^1^H- and ^13^C-NMR spectra were recorded on Varian 400 MHz spectrometers. Chemical shifts (δ) are reported in ppm downfield from CDCl_3_ (δ = 7.26 ppm) for ^1^H-NMR and relative to the central CDCl_3_ resonance (δ = 77.0 ppm) for ^13^C-NMR spectroscopy. Coupling constants (*J*) are given in Hz. ESI-HRMS spectrometry was performed with a Bruker Daltonics LCQDECA ion trap mass spectrometer. Enantiomeric excess was determined by HPLC analysis on Chiralpak AD-H, OD-H, and IC columns in comparison with the authentic racemates. Optical rotation data were recorded on a Rudolph Autopol I automatic polarimeter. Commercial grade solvents were dried and purified by standard procedures as specified in reference [59]. THF (AR grade) was used as received. All other reagents were purchased from commercial sources and were used without further purification.

### 3.2. General Procedure for the Asymmetric Michael Reaction of Cinnamones

9-Amino-*epi*-quinine **3a** (6.5 mg, 0.02 mmol), α,β-unsaturated enones (0.12 mmol), dimedone (14.0 mg, 0.1 mmol), and salicylic acid (4.9 mg, 0.04 mmol) were dissolved in THF (1 mL) without stirring. Once the solution was cooled down to 0 °C, the reaction mixture was stirred for 96 h. After the solvent was removed in vacuo, the residue was purified by flash chromatography on silica gel (EtOAc/petroleum ether) to afford the desired 3,4-dihydropyran.

*2-Hydroxy-2,7,7-trimethyl-4-phenyl-2,3,4,6,7,8-hexahydro-5H-chromen-5-one* (**4aa**) [37]. Colorless oil; 99% yield purified by flash column chromatography; ^1^H-NMR (400 MHz, CDCl_3_) δ (ppm) 7.30–7.23 (m, 2H), 7.19–7.12 (m, 3H), 4.03 (br s, 0.6H), 3.84 (t, *J* = 8.4 Hz, 0.6H), 3.31 (br s, 0.4H), 3.16–3.12 (m, 0.4H), 2.50–2.15 (m, 6H), 1.48 (s, 1.7H), 1.46 (s, 1.3H), 1.19 (s, 1.7H), 1.16 (s, 1.3H), 1.11 (s, 1.7H), 1.07 (s, 1.3H); ^13^C-NMR (100 MHz, CDCl_3_) δ (ppm) 197.3, 196.9, 169.6, 168.6, 144.9, 142.9, 128.8, 128.2, 127.8, 127.7, 126.9, 126.8, 126.5, 125.7, 113.0, 110.5, 99.8, 99.2, 50.6, 50.5, 42.9, 42.8, 42.7, 40.5, 33.9, 32.8, 31.9, 31.4, 29.5, 28.6, 28.3, 27.8, 27.4, 27.1; 97% ee was determined by HPLC on AD-H column, hexane/*i*-propanol (80/20), 1.0 mL/min, UV 254 nm, t_minor_ = 4.820 min, t_major_ = 7.627 min; ${\left[\alpha \right]}_{D}^{20}$ = −4.2° (*c* = 0.028, EtOH).

*4-(2-Chlorophenyl)-2-hydroxy-2,7,7-trimethyl-2,3,4,6,7,8-hexahydro-5H-chromen-5-one* (**4ab**) [60]. Colorless oil; 87% yield purified by flash column chromatography; ^1^H-NMR (400 MHz, CDCl_3_) δ (ppm) 7.36–7.29 (m, 1H), 7.10–7.05 (m, 3H), 4.36–4.24 (m, 1H), 3.83 (br s, 0.5H), 3.43 (br s, 0.5H), 2.49–2.11 (m, 6H), 1.49 (s, 1.4H), 1.48 (s, 1.6H), 1.18 (s, 1.6H), 1.16 (s, 1.4H), 1.09 (s, 1.6H), 1.07 (s, 1.4H); ^13^C-NMR (100 MHz, CDCl_3_) δ (ppm) 196.7, 196.5, 169.7, 140.5, 133.6, 129.9, 129.5, 127.8, 127.6, 126.9, 126.7, 126.6, 112.6, 110.4, 99.7, 98.1, 50.7, 50.6, 42.9, 42.8, 37.7, 31.9, 31.5, 29.4, 28.9, 28.2, 27.9, 27.6, 27.3; 91% ee was determined by HPLC on AD-H column, hexane/*i*-propanol (90/10), 1.0 mL/min, UV 254 nm, t_minor_ = 7.703 min, t_major_ = 9.353 min; ${\left[\alpha \right]}_{D}^{20}$ = −32.8° (*c* = 0.021, EtOH).

*4-(3-chlorophenyl)-2-hydroxy-2,7,7-trimethyl-2,3,4,6,7,8-hexahydro-5H-chromen-5-one* (**4ac**) [37]. Colorless oil; 99% yield purified by flash column chromatography; ^1^H-NMR (400 MHz, CDCl_3_) δ (ppm) 7.19–7.09 (m, 3H), 7.02 (t, *J* = 7.8 Hz, 1H), 4.34 (br s, 0.5H), 3.89 (t, *J* = 4.8 Hz, 0.5H), 3.82–3.77 (m, 1H), 2.48–2.09 (m, 6H), 1.44 (s, 3H), 1.18 (s, 1.3H), 1.15 (s, 1.7H), 1.09 (s, 1.3H), 1.07 (s, 1.7H); ^13^C-NMR (100 MHz, CDCl_3_) δ (ppm) 197.3, 197.0, 169.9, 169.1, 147.3, 145.9, 134.2, 133.9, 129.6, 129.5, 129.1, 127.9, 127.5, 127.1, 126.4, 125.9, 125.27, 125.25, 112.6, 110.3, 99.6, 98.1, 50.6, 50.5, 42.9, 42.8, 42.5, 40.5, 33.9, 33.3, 31.9, 31.5, 29.9, 29.5, 28.6, 28.3, 27.8, 27.4, 26.9; 96% ee was determined by HPLC on AD-H column, hexane/*i*-propanol (80/20), 1.0 mL/min, UV 254 nm, t_minor_ = 5.267 min, t_major_ = 7.583 min; ${\left[\alpha \right]}_{D}^{20}$ = +5.5° (*c* = 0.039, EtOH).

*4-(4-Chlorophenyl)-2-hydroxy-2,7,7-trimethyl-2,3,4,6,7,8-hexahydro-5H-chromen-5-one* (**4ad**) [37]. Colorless oil; 99% yield purified by flash column chromatography; ^1^H-NMR (400 MHz, CDCl_3_) δ (ppm) 7.23 (d, *J* = 8.8 Hz, 1H), 7.21 (d, *J* = 8.8 Hz, 1H), 7.11 (d, *J* = 8.4 Hz, 1H), 7.07 (d, *J* = 8.0 Hz, 1H), 3.95 (d, *J* = 4.4 Hz, 0.5H), 3.81 (d, *J* = 8.6 Hz, 0.5H), 3.16–3.09 (m, 0.5H), 2.95 (br s, 0.4H), 2.49–2.17 (m, 6H), 1.53 (s, 1.5H), 1.50 (s, 1.5H), 1.18 (s, 1.5H), 1.15 (s, 1.5H), 1.10 (s, 1.4H), 1.08 (s, 1.6H); ^13^C-NMR (100 MHz, CDCl_3_) δ (ppm) 196.9, 196.6, 169.3, 168.3, 143.5, 141.9, 132.1, 131.3, 129.1, 128.8, 128.49, 128.47, 128.3, 127.9, 112.9, 110.5, 99.5, 97.9, 50.74, 50.72, 42.9, 42.8, 42.5, 40.3, 33.6, 32.6, 32.0, 31.5, 29.5, 28.7, 28.3, 28.1, 27.5, 27.4; 97% ee was determined by HPLC on OD-H column, hexane/*i*-propanol (80/20), 1.0 mL/min, UV 254 nm, t_minor_ = 5.493 min, t_major_ = 8.417 min; ${\left[\alpha \right]}_{D}^{20}$ = +13.8° (*c* = 0.039, EtOH), [α]_D_^2^° = +10.5° (*c* = 0.039, DCM).

*4-(4-Fluorophenyl)-2-hydroxy-2,7,7-trimethyl-2,3,4,6,7,8-hexahydro-5H-chromen-5-one* (**4ae**) [37]. Colorless oil; 96% yield purified by flash column chromatography; ^1^H-NMR (400 MHz, CDCl_3_) δ (ppm) 7.13 (t, *J* = 6.4 Hz, 1H), 7.08 (t, *J* = 6.4 Hz, 1H), 6.94 (t, *J* = 9.4 Hz, 1H), 6.92 (t, *J* = 8.8 Hz, 1H), 3.94 (br s, 0.5H), 3.82 (dd, *J* = 9.8, 8.2 Hz, 0.5H), 3.51–3.27 (m, 1H), 2.48–2.11 (m, 6H), 1.47 (s, 3H), 1.78 (s, 1.4H), 1.14 (s, 1.6H), 1.09 (s, 1.4H), 1.07 (s, 1.6H); ^13^C-NMR (100 MHz, CDCl_3_) δ (ppm) 197.2, 196.9, 169.5, 168.6, 161.2 (d, *J*^1^_C-F_ = 243.3 Hz), 160.9 (d, *J*^1^_C-F_ = 241.8 Hz), 140.6 (d, *J*^4^_C-F_ = 3.2 Hz), 139.0 (d, *J*^4^_C-F_ = 3.3 Hz), 129.2, 129.1, 128.5 (d, *J*^3^_C-F_ = 7.8 Hz), 128.2 (d, *J*^3^_C-F_ = 7.8 Hz), 115.4 (d, *J*^2^_C-F_ = 21.1 Hz), 115.0 (d, *J*^2^_C-F_ = 21.2 Hz), 114.6, 114.4, 113.0, 110.7, 99.6, 98.1, 60.4, 50.7, 50.6, 42.9, 42.8, 42.7, 40.6, 33.4, 32.6, 31.9, 31.4, 29.5, 28.6, 28.3, 27.9, 27.3, 27.0, 20.9, 14.1; 96% ee was determined by HPLC on AD-H column, hexane/*i*-propanol (80/20), 1.0 mL/min, UV 254 nm, t_minor_ = 5.043 min, t_major_ = 9.330 min; ${\left[\alpha \right]}_{D}^{20}$ = −5.4° (*c* = 0.041, EtOH).

*4-(4-Bromophenyl)-2-hydroxy-2,7,7-trimethyl-2,3,4,6,7,8-hexahydro-5H-chromen-5-one* (**4af**) [37]. Colorless oil; 99% yield purified by flash column chromatography; ^1^H-NMR (400 MHz, CDCl_3_) δ (ppm) 7.37 (d, *J* = 8.4 Hz, 1H), 7.35 (d, *J* = 8.4 Hz, 1H), 7.04 (d, *J* = 8.0 Hz, 1H), 7.00 (d, *J* = 8.4 Hz, 1H), 3.90 (t, *J* = 5.4 Hz, 0.5H), 3.72 (pseudo triple, *J* = 5.4 Hz, 0.6H), 3.50 (br s, 0.5H), 3.19 (br s, 0.4H), 2.48–2.07 (m, 6H), 1.49 (s, 1.6H), 1.48 (s, 1.4H), 1.17 (s, 1.4H), 1.14 (s, 1.6H), 1.09 (s, 1.4H), 1.07 (s, 1.6H); ^13^C-NMR (100 MHz, CDCl_3_) δ (ppm) 197.0, 196.7, 169.4, 168.4, 144.1, 142.6, 131.6, 131.4, 130.9, 129.6, 128.9, 128.7, 120.0, 119.4, 112.8, 110.4, 99.5, 97.9, 50.7, 50.6, 42.9, 42.8, 42.4, 40.3, 33.7, 32.8, 31.9, 31.5, 29.5, 28.7, 28.3, 28.1, 27.4, 27.3; 97% ee was determined by HPLC on AD-H column, hexane/*i*-propanol (80/20), 1.0 mL/min, UV 254 nm, t_minor_ = 5.720 min, t_major_ = 10.570 min; ${\left[\alpha \right]}_{D}^{20}$ = +13.9° (*c* = 0.010, EtOH).

*2-Hydroxy-2,7,7-trimethyl-4-(p-tolyl)-2,3,4,6,7,8-hexahydro-5H-chromen-5-one* (**4ag**) [60]. Colorless oil; 95% yield purified by flash column chromatography; ^1^H-NMR (400 MHz, CDCl_3_) δ (ppm) 7.16–7.01 (m, 4H), 3.99 (br s, 0.6H), 3.80 (pseudo triple, *J* = 8.8 Hz, 0.6H), 3.33 (pseudo double, *J* = 9.6 Hz, 0.8H), 2.50–2.13 (m, 9H), 1.47 (s, 3H), 1.19 (s, 1.7H), 1.15 (s, 1.3H), 1.10 (s, 1.7H), 1.07(s, 1.3H); ^13^C-NMR (100 MHz, CDCl_3_) δ (ppm) 197.1, 196.7, 169.2, 169.1, 141.8, 139.6, 136.2, 135.1, 129.8, 129.0, 128.7, 127.5, 126.8, 126.7, 113.3, 110.5, 99.7, 98.1, 50.7, 42.9, 42.8, 42.7, 40.3, 33.6, 31.9, 31.5, 29.5, 28.8, 28.3, 27.9, 27.4, 27.3, 21.0, 20.9; 97% ee was determined by HPLC on AD-H column, hexane/*i*-propanol (80/20), 1.0 mL/min, UV 254 nm, t_minor_ = 5.313 min, t_major_ = 7.733 min; ${\left[\alpha \right]}_{D}^{20}$ = +6.2° (*c* = 0.041, EtOH).

*2-Hydroxy-4-(4-methoxyphenyl)-2,7,7-trimethyl-2,3,4,6,7,8-hexahydro-5H-chromen-5-one* (**4ah**) [37]. Colorless oil; 89% yield purified by flash column chromatography; ^1^H-NMR (400 MHz, CDCl_3_) δ (ppm) 7.10 (d, *J* = 8.8 Hz, 1.1H), 7.05 (d, *J* = 8.8 Hz, 0.9H), 6.82 (d, *J* = 7.6 Hz, 1.1H), 6.79 (d, *J* = 8.4 Hz, 0.9H), 3.99 (br s, 0.6H), 3.80 (pseudo triple, *J* = 8.8 Hz, 0.6H), 3.75 (s, 3H), 3.59 (br s, 0.4H), 3.31 (br s, 0.6H), 2.49–2.11 (m, 6H), 1.47 (s, 1.7H), 1.46 (s, 1.3H), 1.19 (s, 1.7H), 1.15 (s, 1.3H), 1.10 (s, 1.7H), 1.07 (s, 1.3H); ^13^C-NMR (100 MHz, CDCl_3_) δ (ppm) 197.0, 196.7, 169.1, 167.9, 158.2, 157.6, 136.8, 134.4, 128.7, 127.9, 127.8, 114.5, 113.8, 113.4, 113.3, 110.6, 99.7, 88.1, 55.2, 55.1, 50.8, 42.9, 42.8, 42.7, 40.1, 33.2, 31.9, 31.49, 31.47, 29.5, 28.8, 28.3, 27.9, 27.5; 97% ee was determined by HPLC on AD-H column, hexane/*i*-propanol (80/20), 1.0 mL/min, UV 254 nm, t_minor_ = 6.353 min, t_major_ = 12.270 min; ${\left[\alpha \right]}_{D}^{20}$ = +6.5° (*c* = 0.030, EtOH).

*2-Hydroxy-2,7,7-trimethyl-4-(naphthalen-1-yl)-2,3,4,6,7,8-hexahydro-5H-chromen-5-one* (**4ai**). Colorless oil; 89% yield purified by flash column chromatography; ^1^H-NMR (400 MHz, CDCl_3_) δ (ppm) 8.18 (d, *J* = 8.0 Hz, 0.3H), 8.13 (d, *J* = 8.4 Hz, 0.7H), 7.90 (d, *J* = 8.0 Hz, 0.7H), 7.85 (d, *J* = 7.6 Hz, 0.3H), 7.74 (d, *J* = 8.4 Hz, 0.7H), 7.68 (d, *J* = 8.0 Hz, 0.3H), 7.65–7.54 (m, 2.5H), 7.34 (t, *J* = 7.2 Hz, 1H), 7.27 (d, *J* = 5.6 Hz, 0.5H), 4.82 (d, *J* = 6.8 Hz, 0.8H), 4.70 (br s, 0.5H), 3.39 (br s, 0.8H), 2.63–2.23 (m, 6H), 1.47 (s, 3H), 1.27 (s, 2H), 1.24 (s, 1H), 1.17 (s, 2H), 1.12 (s, 1H); ^13^C-NMR (100 MHz, CDCl_3_) δ (ppm) 196.9, 196.7, 169.7, 138.6, 134.7, 130.8, 129.3, 128.9, 127.9, 126.2, 125.8, 125.4, 125.3, 125.2, 123.3, 122.5, 118.8, 117.1, 110.2, 99.5, 98.3, 50.8, 50.7, 43.1, 42.9, 37.9, 32.1, 31.5, 29.4, 29.2, 28.0, 27.9, 27.83, 27.80; ESI-HRMS calcd. for C_22_H_24_O_3_ + H^+^ 337.1804, found 337.1798; 96% ee was determined by HPLC on AD-H column, hexane/*i*-propanol (80/20), 1.0 mL/min, UV 254 nm, t_minor_ = 4.927 min, t_major_ = 6.790 min; ${\left[\alpha \right]}_{D}^{20}$ = −88.9° (*c* = 0.045, EtOH).

*2-Hydroxy-2,7,7-trimethyl-4-(naphthalen-2-yl)-2,3,4,6,7,8-hexahydro-5H-chromen-5-one* (**4aj**) [37]. Colorless oil; 98% yield purified by flash column chromatography; ^1^H-NMR (400 MHz, CDCl_3_) δ (ppm) 7.81–7.69 (m, 3H), 7.59 (s, 0.5H), 7.55 (s, 0.5H), 7.44–7.35 (m, 2.5H), 7.27–7.26 (m, 0.5H), 4.19 (br s, 0.5H), 4.01 (pseudo triple, *J* = 8.6 Hz, 0.5H), 3.29–3.17 (m, 1H), 2.57–2.17 (m, 6H), 1.49 (s, 3H), 1.26 (s, 2H), 1.19 (s, 1H), 1.13 (s, 1.6H), 1.09 (s, 1.4H); ^13^C-NMR (100 MHz, CDCl_3_) δ (ppm) 197.2, 196.9, 169.7, 168.6, 142.3, 140.5, 133.6, 133.5, 132.3, 132.1, 128.8, 127.9, 127.7, 127.53, 127.52, 127.4, 126.1, 125.7, 125.65, 125.57, 125.52, 125.4, 124.9, 124.8, 113.0, 110.4, 99.8, 98.2, 50.7, 50.6, 42.9, 42.8, 42.5, 40.0, 34.1, 32.9, 32.0, 31.5, 29.9, 29.5, 28.7, 28.4, 27.9, 27.5, 27.2; 98% ee was determined by HPLC on AD-H column, hexane/*i*-propanol (80/20), 1.0 mL/min, UV 254 nm, t_minor_ = 7.073 min, t_major_ = 13.053 min; ${\left[\alpha \right]}_{D}^{20}$ = +60.2° (*c* = 0.051, EtOH).

*4-(Furan-2-yl)-2-hydroxy-2,7,7-trimethyl-2,3,4,6,7,8-hexahydro-5H-chromen-5-one* (**4ak**) [37]. Colorless oil; 78% yield purified by flash column chromatography; ^1^H-NMR (400 MHz, CDCl_3_) δ (ppm) 7.36 (s, 0.6H), 7.29 (s, 0.4H), 6.28 (dd, *J* = 3.0, 1.8 Hz, 1H), 6.02 (d, *J* = 3.2 Hz, 0.7H), 5.95 (d, *J* = 2.0 Hz, 0.3H), 4.15 (d, *J* = 6.8 Hz, 1H ), 4.04 (br s, 1H), 2.55–2.22 (m, 6H), 1.55 (s, 2.2 H), 1.41 (s, 0.8H), 1.17 (s, 3H), 1.11 (s, 3H); ^13^C-NMR (100 MHz, CDCl_3_) δ (ppm) 196.7, 168.9, 155.5, 141.9, 140.3, 110.6, 110.3, 108.4, 106.0, 105.3, 98.3, 50.7, 42.8, 35.9, 32.0, 28.6, 28.2, 27.9, 26.1; 95% ee was determined by HPLC on AD-H column, hexane/*i*-propanol (80/20), 1.0 mL/min, UV 254 nm, t_minor_ = 5.327 min, t_major_ = 6.257 min; ${\left[\alpha \right]}_{D}^{20}$ = −12.9° (*c* = 0.015, EtOH).

*2-Hydroxy-2,7,7-trimethyl-4-(thiophen-2-yl)-2,3,4,6,7,8-hexahydro-5H-chromen-5-one* (**4al**). Brown oil; 97% yield purified by flash column chromatography; ^1^H-NMR (400 MHz, CDCl_3_) δ (ppm) 7.17 (d, *J* = 4.8 Hz, 0.6H), 7.06 (d, *J* = 4.8 Hz, 0.6H), 6.89–6.87 (m, 1H), 6.81 (s, 1H), 4.32 (d, *J* = 5.6 Hz, 0.6H), 4.23 (pseudo triple, *J* = 8.0 Hz, 0.4H), 3.53 (br s, 1H), 2.51-2.11 (m, 6H), 1.52 (s, 2H), 1.46 (s, 1H), 1.19 (s, 2H), 1.16 (s, 1H), 1.10 (s, 2H), 1.07 (s, 1H); ^13^C-NMR (100 MHz, CDCl_3_) δ (ppm) 196.8, 196.7, 169.1, 168.1, 148.7, 147.2, 127.0, 126.4, 124.6, 124.2, 123.8, 123.7, 123.2, 122.4, 112.9, 110.7, 99.4, 98.4, 50.6, 42.8, 42.7, 39.9, 31.9, 31.4, 29.4, 29.3, 28.5, 28.4, 27.7, 27.66, 27.61, 27.3; ESI-HRMS calcd. for C_16_H_20_O_3_S + H^+^ 293.1211, found 293.1206; 91% ee was determined by HPLC on AD-H column, hexane/*i*-propanol (80/20), 1.0 mL/min, UV 254 nm, t_minor_ = 5.850 min, t_major_ = 7.423 min; ${\left[\alpha \right]}_{D}^{20}$ = −18.9° (*c* = 0.047, EtOH).

*2-Hydroxy-2,4,7,7-tetramethyl-2,3,4,6,7,8-hexahydro-5H-chromen-5-one* (**4am**). Colorless oil; 69% yield purified by flash column chromatography; ^1^H-NMR (400 MHz, CDCl_3_) δ (ppm) 3.66–3.35 (m, 1H), 2.77–2.66 (m, 1H), 2.29–2.06 (m, 6H), 1.55 (s, 1.4H), 1.50 (s, 1.6H), 1.23 (d, *J* = 7.6 Hz, 2H), 1.21 (d, *J* = 7.6 Hz, 1H), 1.05 (s, 3H), 1.02 (s, 3H); ^13^C-NMR (100 MHz, CDCl_3_) δ (ppm) 213.5, 198.5, 197.9, 170.7, 166.7, 166.5, 117.1, 114.9, 114.3, 99.1, 97.8, 51.3, 51.2, 51.1, 49.2, 43.2, 42.8, 42.7, 41.5, 39.2, 31.9, 31.4, 29.8, 29.2, 28.4, 28.1, 28.0, 27.2, 26.9, 24.3, 22.9, 22.1, 19.7, 19.4, 18.5; ESI-HRMS calcd. for C_13_H_20_O_3_ + H^+^ 225.1491, found 225.1485; 91% ee was determined by HPLC on IC column, hexane/*i*-propanol (95/5), 1.0 mL/min, UV 254 nm, t_mino__r_ = 49.740 min, t_major_ = 77.910 min; ${\left[\alpha \right]}_{D}^{20}$ = −5.5° (*c* = 0.017, EtOH).

*4-Butyl-2-hydroxy-2,7,7-trimethyl-2,3,4,6,7,8-hexahydro-5H-chromen-5-one* (**4an**). Colorless oil; 75% yield purified by flash column chromatography; ^1^H-NMR (400 MHz, CDCl_3_) δ (ppm) 3.19–3.10 (m, 1H), 2.93–2.87 (m, 0.5H), 2.64 (s, 0.3H), 2.59 (s, 0.3H), 2.30–2.09 (m, 6H), 2.01–1.89 (m, 1H), 1.79–1.73 (m, 1H), 1.65–1.45 (m, 3H), 1.35–1.19 (m, 4H), 1.06–1.02 (m, 6H), 0.89–0.81 (m, 3H); ^13^C-NMR (100 MHz, CDCl_3_) δ (ppm) 213.8, 198.6, 197.8, 171.5, 167.1, 166.7, 115.7, 114.2, 113.9, 99.3, 97.9, 51.4, 51.3, 51.2, 48.3, 43.2, 42.9, 42.8, 38.2, 35.1, 32.1, 31.8, 31.7, 31.3, 30.9, 30.5, 29.8, 29.7, 29.4, 29.3, 28.5, 28.3, 28.2, 28.1, 28.0, 27.9, 27.1, 26.8, 22.8, 22.7, 22.4, 14.1, 14.0; ESI-HRMS calcd. for C_16_H_26_O_3_ + H^+^ 267.1960, found 267.1964; 94% ee was determined by HPLC on IC column, hexane/*i*-propanol (70/30), 1.0 mL/min, UV 254 nm, t_minor_ = 4.440 min, t_major_ = 8.093 min; ${\left[\alpha \right]}_{D}^{20}$ = −13.0° (*c* = 0.034, EtOH).

*2-Hydroxy-9,9-dimethyl-2,3,4,5,6,8,9,10-octahydro-7H-2,6-methanobenzo[b]oxocin-7-one* (**4ao**) [37]. White solid; 91% yield purified by flash column chromatography; ^1^H-NMR (400 MHz, CDCl_3_) δ (ppm) 4.46 (s, 1H ), 3.15 (s, 1H), 2.29 (s, 2H), 2.19 (s, 2H), 2.02 (d, *J* = 12.8 Hz, 1H), 1.93 (d, *J* = 12.4 Hz, 1H), 1.74 (d, *J* = 15.2 Hz, 1H), 1.16 (dd, *J* = 13.0, 3.8 Hz, 1H), 1.59 (d, *J* = 11.2 Hz, 2H), 1.45–1.33 (m, 2H), 1.04 (s, 6H); ^13^C-NMR (100 MHz, CDCl_3_) δ (ppm) 196.7, 171.2, 112.3, 101.3, 50.3, 42.0, 38.7, 36.2, 32.3, 28.5, 28.4, 28.2, 26.9, 19.2; 98% ee was determined by HPLC on IC column, hexane/*i*-propanol (90/10), 1.0 mL/min, UV 254 nm, t_major_ = 12.987 min, t_minor_ = 14.423 min; ${\left[\alpha \right]}_{D}^{20}$ = +4.7° (*c* = 0.023, EtOH).

*2-Ethyl-2-hydroxy-7,7-dimethyl-4-phenyl-2,3,4,6,7,8-hexahydro-5H-chromen-5-one* (**4ap**) [37]. Colorless oil; 63% yield purified by flash column chromatography; ^1^H-NMR (400 MHz, CDCl_3_) δ (ppm) 7.24–7.16 (m, 2.5H), 7.12 (d, *J* = 7.6 Hz, 1H), 7.09–7.06 (m, 1.5H), 3.99 (br s, 0.5H), 3.76 (pseudo triple, *J* = 9.0 Hz, 0.5H), 3.15–3.00 (m, 1H), 2.44–2.09 (m, 6H), 1.69–1.63 (m, 2H), 1.13 (s, 1.6H), 1.09 (s, 1.4H), 1.04 (s, 1.6H), 1.00 (s, 1.4H), 0.89 (0.86) (t, *J* = 7.6 Hz, 3H); ^13^C-NMR (100 MHz, CDCl_3_) δ (ppm) 196.9, 196.5, 169.3, 168.0, 145.1, 142.9, 129.0, 128.3, 126.9, 126.7, 125.8, 113.3, 110.4, 101.3, 99.7, 50.8, 42.9, 42.8, 40.3, 38.0, 33.9, 33.6, 33.2, 32.0, 31.9, 31.5, 29.5, 28.9, 28.3, 27.5, 7.3, 7.2; 98% ee was determined by HPLC on AD-H column, hexane/*i*-propanol (80/20), 1.0 mL/min, UV 254 nm, t_minor_ = 5.247 min, t_major_ = 11.667 min; ${\left[\alpha \right]}_{D}^{20}$ = −5.4° (*c* = 0.018, EtOH).

*2-Hydroxy-2-methyl-4-phenyl-2,3,4,6,7,8-hexahydro-5H-chromen-5-one* (**4ba**) [60]. White solid; 71% yield purified by flash column chromatography; ^1^H-NMR (400 MHz, CDCl_3_) δ (ppm) 7.28 (t, *J* = 7.2 Hz, 1H), 7.24 (d, *J* = 7.2 Hz, 1H), 7.19–7.12 (m, 3H), 4.02 (br s, 0.5H), 3.84 (pseudo triple, *J* = 8.8 Hz, 0.5H), 3.36–3.31(m, 0.5H), 2.63–2.45 (m, 2H), 2.42–2.30 (m, 2H), 2.27–2.15 (m, 2H), 2.11–1.95 (m, 2H), 1.47 (s, 1.5H), 1.45 (s, 1.5H); ^13^C-NMR (100 MHz, CDCl_3_) δ (ppm) 197.2, 196.9, 171.1, 170.1, 144.8, 142.7, 128.9, 128.3, 127.9, 127.7, 126.79, 126.77, 126.6, 125.8, 114.4, 111.6, 99.6, 97.9, 42.8, 40.4, 36.9, 33.9, 32.5, 29.3, 29.2, 27.9, 27.2, 20.8, 20.2; 94% ee was determined by HPLC on AD-H column, hexane/*i*-propanol (80/20), 1.0 mL/min, UV 254 nm, t_minor_ = 5.343 min, t_major_ = 6.693 min; ${\left[\alpha \right]}_{D}^{20}$ = −8.2° (*c* = 0.019, EtOH).

*4-(4-chlorophenyl)-2-hydroxy-2-methyl-2,3,4,6,7,8-hexahydro-5H-chromen-5-one* (**4bd**): White solid; 78% yield purified by flash column chromatography; ^1^H-NMR (400 MHz, CDCl_3_) δ (ppm) 7.25 (d, *J* = 7.2 Hz, 0.6H), 7.21 (d, *J* = 8.8 Hz, 1.5H), 7.11 (d, *J* = 8.8 Hz, 0.6H), 7.07 (d, *J* = 7.6 Hz, 1.4H), 3.95 (pseudo triple, *J* = 4.8 Hz, 0.5H), 3.82 (pseudo triple, *J* = 9.0 Hz, 0.5H), 3.33 (br s, 0.5H), 3.05 (br s, 0.5H), 2.61–2.33 (m, 4H), 2.26–2.17 (m, 2H), 2.07–1.97 (m, 2H), 1.51 (s, 1.6H), 1.49 (s, 1.4H); ^13^C-NMR (100 MHz, CDCl_3_) δ (ppm) 197.2, 196.8, 171.2, 170.2, 143.4, 141.8, 132.0, 131.2, 128.8, 128.4, 128.3, 128.2, 114.2, 111.6, 99.4, 97.8, 42.5, 40.3, 36.9, 33.5, 32.6, 29.3, 29.2, 28.1, 27.2, 20.7, 20.2; ESI-HRMS calcd. for C_16_H_17_ClO_3_ + H^+^ 293.0944, found 293.0937; 95% ee was determined by HPLC on AD-H column, hexane/*i*-propanol (80/20), 1.0 mL/min, UV 254 nm, t_minor_ = 6.397 min, t_major_ = 8.430 min; ${\left[\alpha \right]}_{D}^{20}$ = −11.9° (*c* = 0.007, EtOH). 

*4-(4-Bromophenyl)-2-hydroxy-2-methyl-2,3,4,6,7,8-hexahydro-5H-chromen-5-one* (**4bf**). White solid; 67% yield purified by flash column chromatography; ^1^H-NMR (400 MHz, CDCl_3_) δ (ppm) 7.38 (d, *J* = 8.8 Hz, 0.8H), 7.36 (d, *J* = 8.8 Hz, 1.2H), 7.05 (d, *J* = 10.0 Hz, 0.8H), 7.03 (d, *J* = 8.8 Hz, 1.2H), 3.93 (pseudo triple, *J* = 5.0 Hz, 0.5H), 3.81 (pseudo triple, *J* = 9.2 Hz, 0.5H), 3.23 (br s, 0.5H), 2.99 (br s, 0.5H), 2.61–2.33 (m, 4H), 2.28–2.17 (m, 2H), 2.07–1.97 (m, 2H), 1.52 (s, 1.6H), 1.49 (s, 1.4H); ^13^C-NMR (100 MHz, CDCl_3_) δ (ppm) 197.2, 196.8, 171.2, 170.2, 143.9, 142.4, 131.7, 131.4, 130.9, 129.6, 128.8, 128.6, 120.1, 119.4, 114.2, 111.6, 99.4, 97.8, 42.5, 40.3, 36.9, 36.8, 33.6, 32.7, 29.3, 29.2, 28.1, 27.2, 20.7, 20.2; ESI-HRMS calcd. for C_16_H_17_BrO_3_ + H^+^ 337.0439, found 337.0438; 96% ee was determined by HPLC on AD-H column, hexane/*i*-propanol (80/20), 1.0 mL/min, UV 254 nm, t_minor_ = 6.780 min, t_major_ = 8.967 min; ${\left[\alpha \right]}_{D}^{20}$ = −8.5° (*c* = 0.008, EtOH).

### 3.3. Procedure for the Asymmetric Michael Reaction of Chalcones

Dimedone **1a** (14.0 mg, 0.1 mmol), chalcone **5a** (25.0 mg, 0.12 mmol), and quinine-based squaramide **7** (12.5 mg, 0.02 mmol) were dissolved in chloroform (1.0 mL). After stirring at room temperature for 120 h, triethylamine (41.7 μL, 0.3 mmol) was added in one portion. Subsequently, acetyl chloride (14.2 μL, 0.2 mmol) was added dropwise. Once the reaction completed (1 h), the crude product was purified over silica gel by column chromatography (EtOAc/petroleum ether) to afford **6aa** (37.1 mg, 95 % yield) as a colorless oil.

*5,5-Dimethyl-3-oxo-2-(3-oxo-1,3-diphenylpropyl)cyclohex-1-en-1-yl acetate* (**6aa**) [38,53]. ^1^H-NMR (400 MHz, CDCl_3_) δ (ppm) 7.96 (d, *J* = 8.0 Hz, 2H), 7.54 (t, *J* = 7.2 Hz, 1H), 7.44 (t, *J* = 7.6 Hz, 2H), 7.28–7.22 (m, 4H), 7.15 (t, *J* = 6.6 Hz, 1H), 4.79 (t, *J* = 7.4 Hz, 1H), 3.82 (ABX, *J*_AB_ = 17.2 Hz, *J*_AX_ = 8.0 Hz, 1H), 3.75 (ABX, *J*_AB_ = 17.2, *J*_BX_ = 6.8 Hz, 1H), 2.53 (AB, *J*_AB_ = 18.0 Hz, 1H), 2.42 (AB, *J*_AB_ = 17.2 Hz, 1H), 2.24 (s, 2H), 2.15 (s, 3H), 1.02 (s, 3H), 1.00 (s, 3H); ^13^C-NMR (100 MHz, CDCl_3_) δ (ppm) 198.7, 198.6, 167.3, 163.9, 142.0, 136.8, 132.9, 128.9, 128.5, 128.2, 128.1, 127.4, 126.1, 116.4, 51.7, 42.7, 40.8, 35.7, 32.5, 28.1, 27.9, 20.9; 93% ee was determined by HPLC on AD-H column, hexane/*i*-propanol (80/20), 1.0 mL/min, UV 254 nm, t_minor_ = 7.467 min, t_major_ = 10.760 min; ${\left[\alpha \right]}_{D}^{20}$ = +41.5° (*c* = 0.032, CHCl_3_).

*2-(1-(4-Chlorophenyl)-3-oxo-3-phenylpropyl)-5,5-dimethyl-3-oxocyclohex-1-en-1-yl acetate* (**6ab**). Colorless oil; 99% yield purified by flash column chromatography; ^1^H-NMR (400 MHz, CDCl_3_) δ (ppm) 7.95 (d, *J* = 7.6 Hz, 2H), 7.55 (t, *J* = 7.2 Hz, 1H), 7.45 (t, *J* = 7.4 Hz, 2H), 7.24–7.17 (m, 4H), 4.74 (t, *J* = 7.2 Hz, 1H), 3.79 (ABX, *J*_AB_ = 17.6 Hz, *J*_AX_ = 7.2 Hz, 1H), 3.72 (ABX, *J*_AB_ = 17.8 Hz, *J*_BX_ = 7.8 Hz, 1H), 2.53 (AB, *J*_AB_ = 18.0 Hz, 1H), 2.42 (AB, *J*_AB_ = 17.6 Hz, 1H), 2.24 (s, 2H), 2.20 (s, 3H), 1.02 (s, 3H), 1.00 (s, 3H); ^13^C-NMR (100 MHz, CDCl_3_) δ (ppm) 198.8, 198.3, 167.4, 164.0, 140.5, 136.7, 133.1, 131.9, 128.9, 128.7, 128.6, 128.3, 128.1, 51.7, 42.7, 40.6, 35.3, 32.6, 27.9, 27.8, 20.9; ESI-HRMS calcd. for C_25_H_26_ClO_4_ + H^+^ 425.1514, found 425.1514; 94% ee was determined by HPLC on AD-H column, hexane/*i*-propanol (70/30), 1.0 mL/min, UV 254 nm, t_minor_ = 6.560 min, t_major_ = 11.080 min; ${\left[\alpha \right]}_{D}^{20}$ = +79.6° (*c* = 0.019, EtOH).

*5,5-Dimethyl-3-oxo-2-(3-oxo-3-phenyl-1-(p-tolyl)propyl)cyclohex-1-en-1-yl acetate* (**6ac**). Colorless oil; 96% yield purified by flash column chromatography; ^1^H-NMR (400 MHz, CDCl_3_) δ (ppm) 7.96 (d, *J* = 7.2 Hz, 2H), 7.54 (t, *J* = 7.4 Hz, 1H), 7.44 (t, *J* = 7.6 Hz, 2H), 7.16 (d, *J* = 8.0 Hz, 2H), 7.05 (d, *J* = 8.0 Hz, 2H), 4.74 (t, *J* = 7.4 Hz, 1H), 3.82 (ABX, *J*_AB_ = 17.2 Hz, *J*_AX_ = 8.0 Hz, 1H), 3.73 (ABX, *J*_AB_ = 17.2 Hz, *J*_BX_ = 6.8 Hz, 1H), 2.53 (AB, *J*_AB_ = 17.6 Hz, 1H), 2.43 (AB, *J*_AB_ = 17.6 Hz, 1H), 2.28 (s, 3H), 2.24 (s, 2H), 2.17 (s, 3H), 1.02 (s, 3H), 1.01 (s, 3H); ^13^C-NMR (100 MHz, CDCl_3_) δ (ppm) 198.9, 198.8, 167.5, 163.8, 138.9, 136.9, 135.7, 132.9, 129.1, 128.9, 128.5, 128.1, 127.4, 51.8 42.8, 40.9, 35.5, 32.6, 28.0, 27.9, 20.9; ESI-HRMS calcd. for C_26_H_29_O_4_ + H^+^ 405.2060, found 405.2061; 93% ee was determined by HPLC on AD-H column, hexane/*i*-propanol (70/30), 1.0 mL/min, UV 254 nm, t_minor_ = 6.467 min, t_major_ = 13.007 min; ${\left[\alpha \right]}_{D}^{20}$ = +70.1° (*c* = 0.018, EtOH).

*5,5-Dimethyl-3-oxo-2-(3-oxo-3-phenyl-1-(thiophen-2-yl)propyl)cyclohex-1-en-1-yl acetate* (**6ad**). Colorless oil; 95% yield purified by flash column chromatography; ^1^H-NMR (400 MHz, CDCl_3_) δ (ppm) 7.72–7.67 (m, 1H), 7.54 (d, *J* = 4.0 Hz, 1H), 7.20–7.16 (m, 4H), 7.10–7.09 (m, 1H), 7.05–7.00 (m, 1H), 4.71 (t, *J* = 7.4 Hz, 1H), 3.72 (ABX, *J*_AB_ = 16.2 Hz, *J*_AX_ = 8.4 Hz, 1H), 3.57 (ABX, *J*_AB_ = 16.4 Hz, *J*_BX_ = 6.4 Hz, 1H), 2.45 (AB, *J*_AB_ = 18.0 Hz, 1H), 2.36 (AB, *J*_AB_ = 17.6 Hz, 1H), 2.16 (s, 2H), 2.12 (s, 3H), 0.93 (s, 6H); ^13^C-NMR (100 MHz, CDCl_3_) δ (ppm) 198.8, 191.7, 167.4, 164.1, 144.3, 141.8, 133.7, 132.1, 128.7, 128.2, 128.0, 127.4, 126.2, 51.7, 42.7, 41.3, 36.0, 32.6, 27.9, 27.8, 20.9; ESI-HRMS calcd. for C_23_H_25_O_4_S + H^+^ 397.1468, found 397.1468; 91% ee was determined by HPLC on AD-H column, hexane/*i*-propanol (70/30), 1.0 mL/min, UV 254 nm, t_minor_ = 6.903 min, t_major_ = 10.567 min; ${\left[\alpha \right]}_{D}^{20}$ = +29.6° (*c* = 0.016, EtOH).

*5,5-Dimethyl-2-(1-(naphthalen-2-yl)-3-oxo-3-phenylpropyl)-3-oxocyclohex-1-en-1-yl acetate* (**6ae**). Colorless oil; 68% yield purified by flash column chromatography; ^1^H-NMR (400 MHz, CDCl_3_) δ (ppm) 8.00 (d, *J* = 8.0 Hz, 2H), 7.78–7.73 (m, 4H), 7.56 (t, *J* =7.4 Hz, 1H), 7.46 (t, *J* = 7.4 Hz, 2H), 7.43–7.39 (m, 3H), 4.97 (t, *J* = 7.2 Hz, 1H), 3.94 (ABX, *J*_AB_ = 17.6 Hz, *J*_AX_ = 8.4 Hz, 1H), 3.88 (ABX, *J*_AB_ = 17.0 Hz, *J*_BX_ = 7.0 Hz, 1H), 2.56 (AB, *J*_AB_ = 18.0 Hz, 1H), 2.43 (AB, *J*_AB_ = 18.0 Hz, 1H), 2.28 (AB, *J*_AB_ = 16.8 Hz, 1H), 2.24 (AB, *J*_AB_ = 17.6 Hz, 1H), 2.15 (s, 3H), 1.04 (s, 3H), 1.01 (s, 3H); ^13^C-NMR (100 MHz, CDCl_3_) δ (ppm) 198.8, 198.6, 167.4, 164.1, 139.5, 136.9, 133.3, 133.0, 132.0, 128.8, 128.5, 128.1, 127.9, 127.7, 127.5, 126.5, 125.8, 125.6, 125.3, 51.7, 42.8, 40.8, 35.9, 32.6, 27.9, 27.8, 20.9; ESI-HRMS calcd. for C_29_H_29_O_4_ + H^+^ 441.2060, found 441.2061; 91% ee was determined by HPLC on AD-H column, hexane/*i*-propanol (70/30), 1.0 mL/min, UV 254 nm, t_minor_ = 7.943 min, t_major_ = 11.667 min; ${\left[\alpha \right]}_{D}^{20}$ = +79.7° (*c* = 0.011, EtOH).

*2-(3-(4-Chlorophenyl)-3-oxo-1-phenylpropyl)-5,5-dimethyl-3-oxocyclohex-1-en-1-yl acetate* (**6af**). White solid; 98% yield purified by flash column chromatography; ^1^H-NMR (400 MHz, CDCl_3)_ δ (ppm) 7.90 (d, *J* = 8.4 Hz, 2H), 7.41 (d, *J* = 8.4 Hz, 2H), 7.26–7.23 (m, 4H), 7.20–7.23 (m, 1H), 4.77 (t, *J* = 7.4 Hz, 1H), 3.82 (ABX, *J*_AB_ = 17.2 Hz, *J*_AX_ = 8.0 Hz, 1H), 3.70 (ABX, *J*_AB_ = 17.2 Hz, *J*_BX_ = 6.8 Hz, 1H), 2.54 (AB, *J*_AB_ = 17.6 Hz, 1H), 2.42 (AB, *J*_AB_ = 18.0 Hz, 1H), 2.24 (s, 2H), 2.17 (s, 3H), 1.02 (s, 3H), 1.00 (s, 3H); ^13^C-NMR (100 MHz, CDCl_3_) δ (ppm) 198.9, 197.5, 167.3, 164.0, 141.8, 139.3, 135.1, 129.5, 128.75, 128.72, 128.2, 127.4, 126.2, 51.7, 42.7, 40.7, 35.8, 32.5, 27.8, 20.9; ESI-HRMS calcd. for C_25_H_26_ClO_4_ + H^+^ 425.1514, found 425.1514; 87% ee was determined by HPLC on AD-H column, hexane/*i*-propanol (70/30), 1.0 mL/min, UV 254 nm, t_minor_ = 9.913 min, t_major_ = 15.547 min; ${\left[\alpha \right]}_{D}^{20}$ = +57.9° (*c* = 0.016, EtOH).

*5,5-Dimethyl-3-oxo-2-(3-oxo-1-phenyl-3-(p-tolyl)propyl)cyclohex-1-en-1-yl acetate* (**6ag**). Colorless oil; 99% yield purified by flash column chromatography; ^1^H-NMR (400 MHz, CDCl_3_) δ (ppm) 7.87 (d, *J* = 8.0 Hz, 2H), 7.29–7.23 (m, 6H), 7.19–7.11 (m, 1H), 4.79 (t, *J* = 7.4 Hz, 1H), 3.79 (ABX, *J*_AB_ = 16.4 Hz, *J*_AX_ = 7.6 Hz, 1H), 3.73 (ABX, *J*_AB_ = 16.8 Hz, *J*_BX_ = 6.8 Hz, 1H), 2.53 (AB, *J*_AB_ = 18.0 Hz, 1H), 2.42 (AB, *J*_AB_ = 17.6 Hz, 1H), 2.40 (s, 3H), 2.25 (s, 2H), 2.17 (s, 3H), 1.03 (s, 3H), 1.01 (s, 3H); ^13^C-NMR (100 MHz, CDCl_3_) δ (ppm) 198.8, 198.3, 167.4, 163.8, 143.7, 142.1, 134.4, 129.1, 129.0, 128.2, 128.1, 127.5, 126.1, 51.7, 42.7, 40.6, 35.7, 32.6, 27.9, 21.6, 20.9; ESI-HRMS calcd. for C_26_H_29_O_4_ + H^+^ 405.2060, found 405.2061; 95% ee was determined by HPLC on AD-H column, hexane/*i*-propanol (80/20), 1.0 mL/min, UV 254 nm, t_minor_ = 9.573 min, t_major_ = 13.540 min; ${\left[\alpha \right]}_{D}^{20}$ = +77.1° (*c* = 0.018, EtOH)

*5,5-Dimethyl-3-oxo-2-(3-oxo-1-phenyl-3-(thiophen-2-yl)propyl)cyclohex-1-en-1-yl acetate* (**6ah**). Colorless oil; 99% yield purified by flash column chromatography; ^1^H-NMR (400 MHz, CDCl_3_) δ (ppm) 7.88 (d, *J* = 7.6 Hz, 2H), 7.47 (t, *J* = 7.2 Hz, 1H), 7.36 (t, *J* = 7.4 Hz, 2H), 7.03–6.97 (m, 1H), 6.83–6.74 (m, 2H), 4.97 (t, *J* = 7.0 Hz, 1H), 3.82 (ABX, *J*_AB_ = 17.4 Hz, *J*_AX_ = 7.8 Hz, 1H), 3.70 (ABX, *J*_AB_ = 17.6 Hz, *J*_BX_ = 6.4 Hz, 1H), 2.49 (AB, *J*_AB_ = 18.0 Hz, 1H), 2.38 (AB, *J*_AB_ = 17.6 Hz, 1H), 2.19 (s, 2H), 2.15 (s, 3H), 0.97 (s, 3H), 0.95 (s, 3H); ^13^C-NMR (100 MHz, CDCl_3_) δ (ppm) 198.4, 198.0, 167.3, 164.1, 145.7, 136.6, 133.0, 128.5, 128.2, 128.1, 126.4, 124.1, 123.3, 51.6, 42.6, 42.3, 32.6, 31.5, 27.9, 27.8, 20.9; ESI-HRMS calcd. for C_23_H_25_O_4_S + H^+^ 397.1468, found 397.1469; 97% ee was determined by HPLC on AD-H column, hexane/*i*-propanol (80/20), 1.0 mL/min, UV 254 nm, t_minor_ = 7.623 min, t_major_ = 9.193 min; ${\left[\alpha \right]}_{D}^{20}$ = +102.9° (*c* = 0.019, EtOH)

*3-Oxo-2-(3-oxo-1,3-diphenylpropyl)cyclohex-1-en-1-yl acetate* (**6ba**). Colorless oil; 93% yield purified by flash column chromatography; ^1^H-NMR (400 MHz, CDCl_3_) δ (ppm) 7.97 (d, *J* = 7.6 Hz, 2H), 7.55 (t, *J* = 7.2 Hz, 1H), 7.44 (t, *J* =7.4 Hz, 2H), 7.29–7.23 (m, 4H), 7.16 (t, *J* = 6.6 Hz, 1H), 4.80 (t, *J* = 7.2 Hz, 1H), 3.81 (ABX, *J*_AB_ = 17.8 Hz, *J*_AX_ = 6.6 Hz, 1H), 3.75 (ABX, *J*_AB_ = 17.6 Hz, *J*_BX_ = 7.6 Hz, 1H), 2.65 (dt, *J* = 18.0, 6.4 Hz, 1H), 2.53 (dt, *J* = 18.0, 5.9 Hz, 1H), 2.37 (t, *J* = 6.4 Hz, 2H), 2.17 (s, 3H), 1.98–1.89 (m, 2H); ^13^C-NMR (100 MHz, CDCl_3_) δ (ppm) 198.8, 198.7, 167.3, 165.6, 142.0, 136.8, 132.9, 130.1, 128.5, 128.1, 128.0, 127.5, 126.1, 40.8, 37.9, 35.7, 29.0, 20.9, 20.7; ESI-HRMS calcd. for C_23_H_23_O_4_ + H^+^ 363.1591, found 363.1591; 91% ee was determined by HPLC on AD-H column, hexane/*i*-propanol (70/30), 1.0 mL/min, UV 254 nm, t_minor_ = 7.667 min, t_major_ = 10.660 min; ${\left[\alpha \right]}_{D}^{20}$ = +92.4° (*c* = 0.016, EtOH).

*3-Oxo-2-(3-oxo-1,3-diphenylpropyl)cyclopent-1-en-1-yl acetate* (**6ca**). Colorless oil; 31% yield purified by flash column chromatography; ^1^H-NMR (400 MHz, CDCl_3_) δ (ppm) 7.94 (d, *J* = 7.6 Hz, 2H), 7.52 (t, *J* = 7.2 Hz, 1H), 7.42 (t, *J* = 7.0 Hz, 2H), 7.36–7.34 (m, 2H), 7.26 (t, *J* = 6.8 Hz, 2H), 7.18 (t, *J* = 7.0 Hz, 1H), 4.49 (t, *J* = 7.2 Hz, 1H), 4.06 (ABX, *J*_AB_ = 17.6 Hz, *J*_BX_ = 8.8 Hz, 1H), 3.56 (ABX, *J*_AB_ = 17.6 Hz, *J*_BX_ = 6.0 Hz, 1H), 2.93–2.79 (m, 2H), 2.47–2.38 (m, 2H), 2.21 (s, 3H); ^13^C-NMR (100 MHz, CDCl_3_) δ (ppm) 205.3, 198.3, 176.7, 166.5, 141.9, 136.8, 133.1, 129.9, 128.6, 128.5, 128.0, 127.8, 126.7, 40.8, 35.8, 34.7, 26.9, 21.1; ESI-HRMS calcd. for C_22_H_22_O_4_ + H^+^ 349.1440, found 349.1437; 57% ee was determined by HPLC on AD-H column, hexane/*i*-propanol (80/20), 1.0 mL/min, UV 254 nm, t_minor_ = 8.787 min, t_major_ = 12.983 min; ${\left[\alpha \right]}_{D}^{20}$ = +29.0° (*c* = 0.014, EtOH).

*5,5-Dimethyl-3-oxo-2-(1-oxo-1-phenylhexan-3-yl)cyclohex-1-en-1-yl acetate* (**6ai**). Colorless oil; 93% yield purified by flash column chromatography; ^1^H-NMR (400 MHz, CDCl_3_) δ (ppm) 7.91 (d, *J* = 7.2 Hz, 2H), 7.52 (t, *J* = 7.2 Hz, 1H), 7.42 (t, *J* = 7.4 Hz, 2H), 3.32 (ABX, *J*_AB_ = 16.4 Hz, *J*_BX_ = 6.8 Hz, 1H), 3.23 (ABX, *J*_AB_ = 16.0 Hz, *J*_BX_ = 6.4 Hz, 1H), 2.46 (AB, *J*_AB_ = 18.0 Hz, 1H), 2.41 (AB, *J*_AB_ = 18.0 Hz, 1H), 2.27 (AB, *J*_AB_ = 15.6 Hz, 1H), 2.21 (AB, *J*_AB_ = 15.2 Hz, 1H), 2.20 (s, 3H), 1.77–1.68 (m, 2H), 1.54–1.47 (m, 1H), 1.21–1.16 (m, 2H), 1.06 (s, 3H), 0.99 (s, 3H), 0.85 (t, *J* = 7.2 Hz, 3H); ^13^C-NMR (100 MHz, CDCl_3_) δ (ppm) 199.7, 199.4, 167.7, 163.8, 137.2, 132.8, 128.6, 128.4, 128.1, 52.1, 42.7, 42.0, 35.0, 32.4, 31.6, 28.0, 27.9, 21.1, 20.9, 13.9; ESI-HRMS calcd. for C_22_H_29_O_4_ + H^+^ 357.2066, found 357.2064; 52% ee was determined by HPLC on IC column, hexane/*i*-propanol (99/1), 1.0 mL/min, UV 254 nm, t_major_ = 39.457 min, t_minor_ = 42.963 min; ${\left[\alpha \right]}_{D}^{20}$ = +4.75° (*c* = 0.022, EtOH).

### 3.4. Preparation of 4H-Pyran via Dehydrating 

Thionyl chloride (7.3 μL, 11.9 mg, 0.1 mmol) was added dropwise to a solution of **4a** (28.6 mg, 0.1 mmol, 97% ee) and pyridine (14.1 μL, 15.8 mg, 0.2 mmol) in DCM (1.0 mL) at rt. After the reaction completed, the solvent was removed under reduced pressure. The residue was subjected to silica gel flash chromatography (EtOAc/petroleum ether) to provide **8** (19.3 mg, 72% yield) as a white solid.

*2,7,7-Trimethyl-4-phenyl-4,6,7,8-tetrahydro-5H-chromen-5-one* (**8**): ^1^H-NMR (400 MHz, CDCl_3_) δ (ppm) 7.29–7.24 (m, 4H), 7.17–7.13 (m, 1H), 4.90 (d, *J* = 4.8 Hz, 2H), 4.28 (d, *J* = 4.0 Hz, 1H), 2.39 (s, 2H), 2.22 (AB, *J*_AB_ = 16.0 Hz, 1H), 2.16 (AB, *J*_AB_ = 16.4 Hz, 1H), 1.88 (s, 3H), 1.09 (s, 3H), 1.03 (s, 3H); ^13^C-NMR (100 MHz, CDCl_3_) δ (ppm) 197.3, 164.7, 145.8, 145.6, 128.2, 127.8, 126.2, 112.2, 104.5, 50.8, 41.4, 35.2, 31.9, 29.1, 27.6, 18.6; ESI-HRMS calcd. for C_18_H_20_O_2_ + H^+^ 269.1542, found 269.1541; 98% ee was determined by HPLC on OD-H column, hexane/*i*-propanol (90/10), 1.0 mL/min, UV 254 nm, t_minor_ = 5.880 min, t_major_ = 8.550 min; ${\left[\alpha \right]}_{D}^{20}$ = −182.7° (*c* = 0.024, EtOH).

### 3.5. Preparation of Fused Dihydrofuran via Stereoselective Oxidative Cyclization

After the initial Michael addition between **5a** (49.9 mg, 0.24 mmol) and **1a** (28.0 mg, 0.2 mmol) was completed, the corresponding adduct was purified via flash column chromatography. Subsequently, the mixture of PhIO (66 mg, 0.3 mmol) and Michael adduct (69.6 mg, 0.2 mmol) in H_2_O (1 mL) was treated with Bu_4_NI (111 mg, 0.3 mmol). The reaction mixture was warmed up to 30 °C and allowed to stir for 16 h. The reaction was followed by TLC until completion. The reaction mixture was successively quenched with saturated Na_2_S_2_O_3_ (25 mL) and extracted by dichloromethane (25 mL × 3). The organic layer was dried over Na_2_SO_4_ and concentrated in vacuo. The residue was purified by column chromatography on silica gel (EtOAc/petroleum ether) to furnish 2,3-dihydrobenzofuran **9** in 61% yield as a colorless oil.

*2-Benzoyl-6,6-dimethyl-3-phenyl-3,5,6,7-tetrahydrobenzofuran-4(2H)-one* (**9**) [61]. White solid; 61% yield purified by flash column chromatography; ^1^H-NMR (400 MHz, CDCl_3_) δ (ppm) 7.82 (d, *J* = 7.2 Hz, 2H), 7.61 (t, *J* = 7.4 Hz, 1H), 7.46 (t, *J* = 7.6 Hz, 2H), 7.36 (t, *J* =7.2 Hz, 2H), 7.30–7.28 (m, 1H), 7.25–7.23 (m, 2H), 5.89 (d, *J* = 4.8 Hz, 1H), 4.40 (d, *J* = 4.0 Hz, 1H), 2.63 (ABX, *J*_AB_ = 18.0 Hz, *J*_AX_ = 1.6 Hz, 1H), 2.53 (AB, *J*_AB_ = 17.6 Hz, 1H), 2.25 (AB, *J*_AB_ = 16.4 Hz, 1H), 2.18 (AB, *J*_AB_ = 16.4 Hz, 1H), 1.17 (s, 6H); ^13^C-NMR (100 MHz, CDCl_3_) δ (ppm) 193.5, 192.8, 176.3, 141.2, 134.1, 133.1, 129.0, 128.89, 128.86, 127.6, 127.3, 115.1, 91.8, 51.1, 48.9, 37.6, 34.3, 29.0, 28.3; 93% ee was determined by HPLC on AD-H column, hexane/*i*-propanol (80/20), 1.0 mL/min, UV 254 nm, t_minor_ = 10.843 min, t_major_ = 15.107 min; ${\left[\alpha \right]}_{D}^{20}$ = −44.6° (*c* = 0.021, EtOH).

## 4. Conclusions

In summary, we have successfully developed an enantioselective Michael addition of cyclic β-diones to α,β-unsaturated enones in the presence of quinine-based primary amine or squaramide. These asymmetric processes displayed especially broad substrate generalities, and various cinnamones and chalcones furnished the desired adducts in good to high yields. Although chalcones proved to be a class of challenging acceptors in the precedent study [38], good reactivities and excellent enantiopurities were achieved in the case of their Michael addition with cyclic β-diones via our protocol.

## Figures and Tables

**Table 1:**
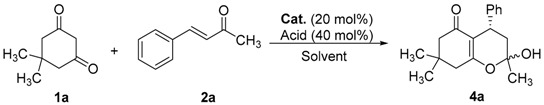
Optimization of reaction conditions for the Michael addition of dimedone **1a** to cinnamone **2a**. ^a^

Entry	Cat.	Acid	Solvent	Time (h)	Yield (%) ^b^	ee (%) ^c^
1	**3a**	TsOH	toluene	48	60	66
2	**3a**	TFA	toluene	48	65	75
3	**3a**	AcOH	toluene	48	82	85
4	**3a**	BA	toluene	24	89	90
5	**3a**	ONBA	toluene	36	91	90
6	**3a**	PNBA	toluene	36	96	89
7	**3a**	OFBA	toluene	24	96	88
8	**3a**	*p*-MeOC_6_H_4_CO_2_H	toluene	36	91	87
9	**3a**	SA	toluene	24	99	90
10	**3b**	SA	toluene	96	89	−82
11	**3c**	SA	toluene	36	89	−82
12	**3a**	SA	PhCF_3_	24	96	85
13	**3a**	SA	DCM	24	55	83
14	**3a**	SA	EtOH	24	99	69
15	**3a**	SA	THF	24	99	94
16 ^d^	**3a**	SA	THF	24	99	94
17 ^d,e^	**3a**	SA	THF	96	99	97

**^a^** Unless otherwise noted, the reaction was performed with 0.1 mmol of **1a**, 0.15 mmol of **2a**, 20 mol % of **3a**, and 40 mol % of acid in 1 mL of solvent at room temperature (r.t.). TsOH = *p-*toluenesulfonic acid, TFA = trifluoroacetic acid, BA = benzoic acid, ONBA = *o*-nitrobenzoic acid, PNBA = *p*-nitrobenzoic acid, OFBA = *o*-fluorobenzoic acid, DCM = dichloromethane. ^b^ Isolated yield after flash chromatography on silica gel. ^c^ Determined by HPLC analysis on a chiral stationary phase (Chiralcel AD-H). ^d^ 0.12 mmol of **2a** was employed. ^e^ Carried out at 0 °C.

**Table 2:**
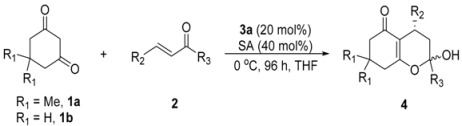
Substrate scope of the Michael addition of cyclic β-diones to cinnamones and its analogues. ^a^

Entry	1	R_2_	R_3_	2	4	Yield (%) ^b^	ee (%) ^c^
1	**1a**	Ph	Me	**2a**	**4aa**	99 (95) ^d^	97 (94) ^d^
2	**1a**	*o*-ClC_6_H_4_	Me	**2b**	**4ab**	87	91
3	**1a**	*m*-ClC_6_H_4_	Me	**2c**	**4ac**	99	96
4	**1a**	*p*-ClC_6_H_4_	Me	**2d**	**4ad**	99	97 (*S*) ^e^
5	**1a**	*p*-FC_6_H_4_	Me	**2e**	**4ae**	96	96
6	**1a**	*p*-BrC_6_H_4_	Me	**2f**	**4af**	99	97
7	**1a**	*p*-MeC_6_H_4_	Me	**2g**	**4ag**	95	97
8	**1a**	*p*-MeOC_6_H_4_	Me	**2h**	**4ah**	89	97
9	**1a**	1-naphthyl	Me	**2i**	**4ai**	89	96
10	**1a**	2-naphthyl	Me	**2j**	**4aj**	98	98
11	**1a**	2-furanyl	Me	**2k**	**4ak**	78	95
12	**1a**	2-thiophenyl	Me	**2l**	**4al**	97	91
13	**1a**	Me	Me	**2m**	**4am**	69	91
14	**1a**	*n*-Bu	Me	**2n**	**4an**	75	94
15	**1a**	-C_3_H_6_-		**2o**	**4ao**	91	98
16	**1a**	Ph	Et	**2p**	**4ap**	63	98
17	**1b**	Ph	Me	**2a**	**4ba**	71	94
18	**1b**	*p*-ClC_6_H_4_	Me	**2d**	**4bd**	78	95
19	**1b**	*p*-BrC_6_H_4_	Me	**2f**	**4bf**	67	96

**^a^** Unless otherwise noted, the reaction was performed with 0.1 mmol of **1a**, 0.12 mmol of **2a**, 20 mol% of **3a**, and 40 mol % of salicylic acid in 1 mL of THF at 0 °C for 96 h. ^b^ Isolated yield after flash chromatography on silica gel. ^c^ Determined by HPLC analysis on a chiral stationary phase. ^d^ Data within parentheses is that performed on an one-mmole scale. ^e^ Configuration of **4ad**.

**Table 3:**
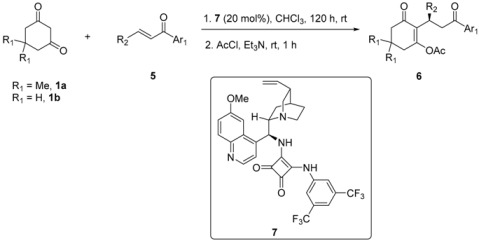
Substrate scope of the Michael addition of cyclic β-diones to chalcones. ^a^

Entry	1	R_2_	Ar_1_	5	6	Yield (%) ^b^	ee (%) ^c^
1	**1a**	Ph	Ph	**5a**	**6aa**	95 (99) ^d^	93 (91) ^d^ (*R*) ^e^
2	**1a**	*p*-ClC_6_H_4_	Ph	**5b**	**6ab**	99	94
3	**1a**	*p*-MeC_6_H_4_	Ph	**5c**	**6ac**	96	93
4 ^f^	**1a**	2-thiophenyl	Ph	**5d**	**6ad**	95	91
5 ^f^	**1a**	2-naphthyl	Ph	**5e**	**6ae**	68	91
6	**1a**	Ph	*p*-ClC_6_H_4_	**5f**	**6af**	98	87
7	**1a**	Ph	*p*-MeC_6_H_4_	**5g**	**6ag**	99	95
8	**1a**	Ph	2-thiophenyl	**5h**	**6ah**	99	97
9 ^f^	**1b**	Ph	Ph	**5a**	**6ba**	93	91
10 ^g^	**1a**	*n*-Pr	Ph	**5i**	**6ai**	93	52

**^a^** Unless otherwise noted, the Michael addition was performed with 0.1 mmol of **1**, 0.12 mmol of **5**, and 20 mol % of **7** in 1 mL of chloroform at rt for 120 h. ^b^ Isolated yield after flash chromatography on silica gel. ^c^ Determined by HPLC analysis on a chiral stationary phase. ^d^ Data within parentheses is that performed on a one-mmole scale. ^e^ Configuration of **6aa**. ^f^ Performed with 168 h. ^g^ Performed with 72 h.

## Supplementary Materials

The supplementary materials are available online.
