# Supplementary material for: Enantioselective Michael Addition of Cyclic β-Diones to α,β-Unsaturated Enones Catalyzed by Quinine-Based Organocatalysts
Source: Molecules. 2017 Jun 30;22(7):1096. doi: 10.3390/molecules22071096 (PMC6152274; doi:10.3390/molecules22071096)
Supplement: Supplementary file 1 [file molecules-22-01096-s001.pdf]

Highly Enantioselective Michael Addition of Cyclic β-Diones to α,β-Unsaturated Enones Catalyzed by Quinine-based Organocatalysts

Qingqing Wang [1], Wei Wang [1], Ling Ye [2], Xuejun Yang [1], Xinying Li [1], Zhigang Zhao [1] and Xuefeng Li [1,*]

[1] College of Chemistry and Environment Protection Engineering, Southwest Minzu University, Chengdu 610041, China.

[2] Faculty of Geosciences and Environmental Engineering, Southwest Jiaotong University, Chengdu 610031, China.

* Correspondence: lixuefeng@swun.edu.cn

Table of contents:

1. Optimization of reaction conditions for Michael addition of dimedone **1a** to chalcone **5a** ---2

2. NMR spectra and HPLC chromatograms of products----------------------------------3

Table 1. Optimization of reaction conditions for Michael addition of dimedone **1a** to chalcone **5a**.[a]

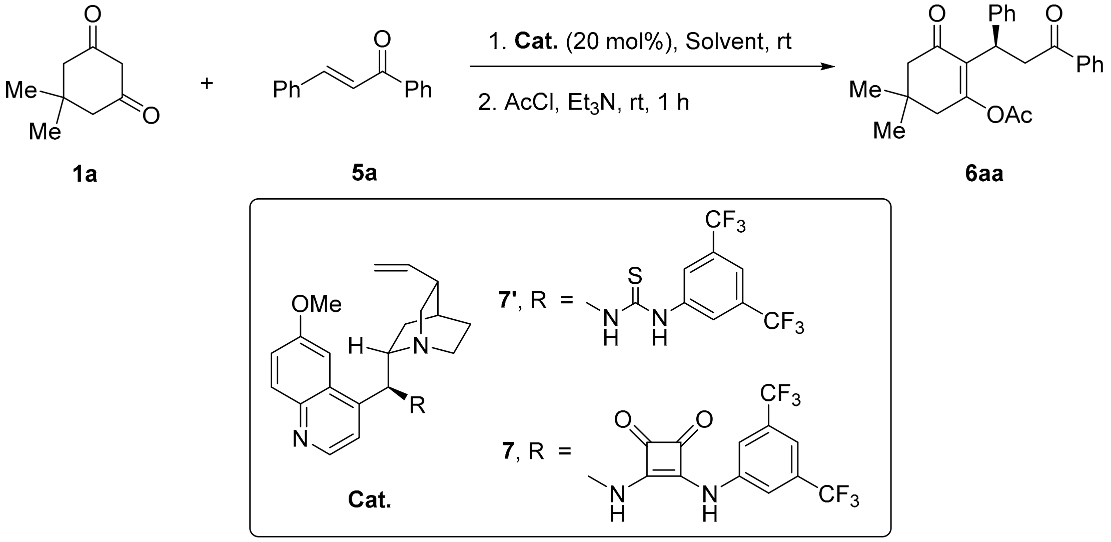

Entry	**Cat.**	Solvent	Time (h)	Yield (%) [b]	ee (%) [c]
1	**7'**	toluene	96	96	75
2	**7**	toluene	96	99	90
3	**7**	$PhCF_3$	168	76	88
4	**7**	DCM	120	99	92
5	**7**	DCE	120	92	92
6	**7**	$CHCl_3$	120	95	93
7	**7**	EtOAc	168	87	90

[a] Unless otherwise noted, the Michael addition was performed with 0.1 mmol of **1a**, 0.12 mmol of **5a**, 20 mol% of catalyst in 1 mL of solvent at rt. [b] Isolated yield after flash chromatography on silica gel. [c] Determined by HPLC analysis on a chiral stationary phase.

NMR spectra and HPLC chromatograms of products

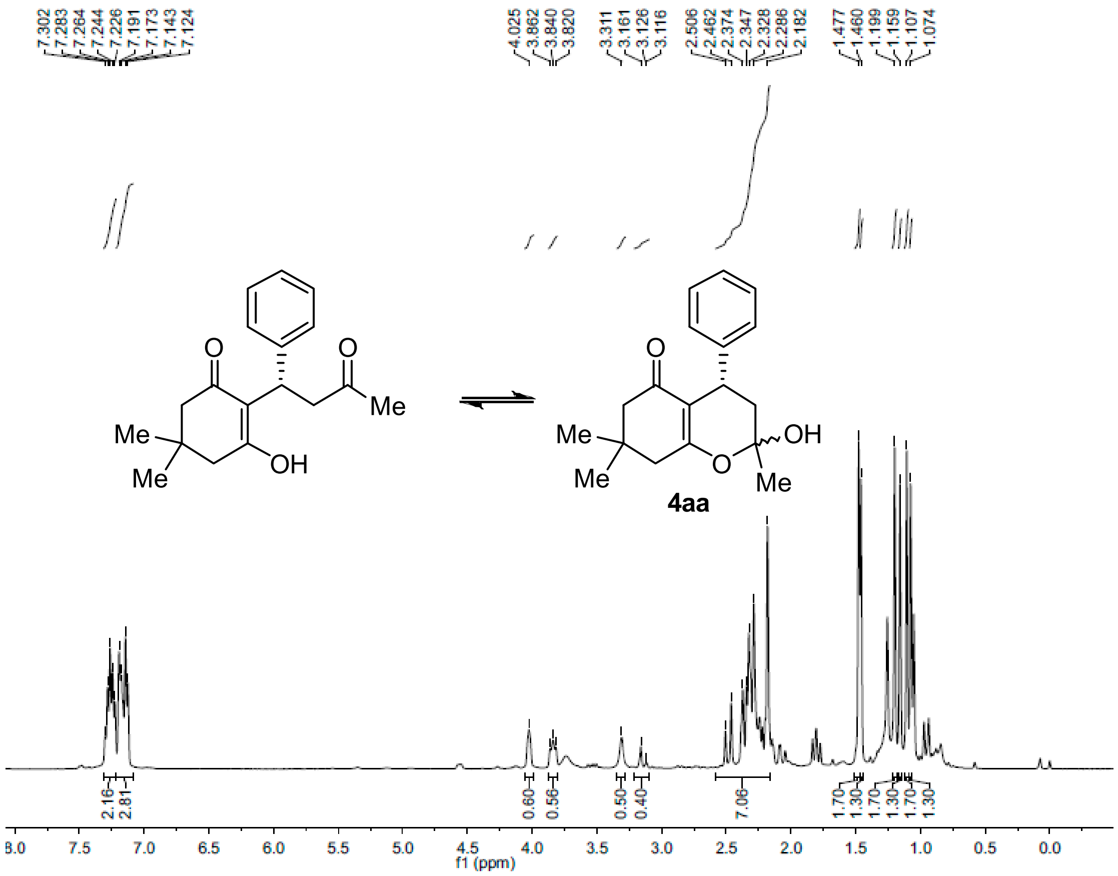

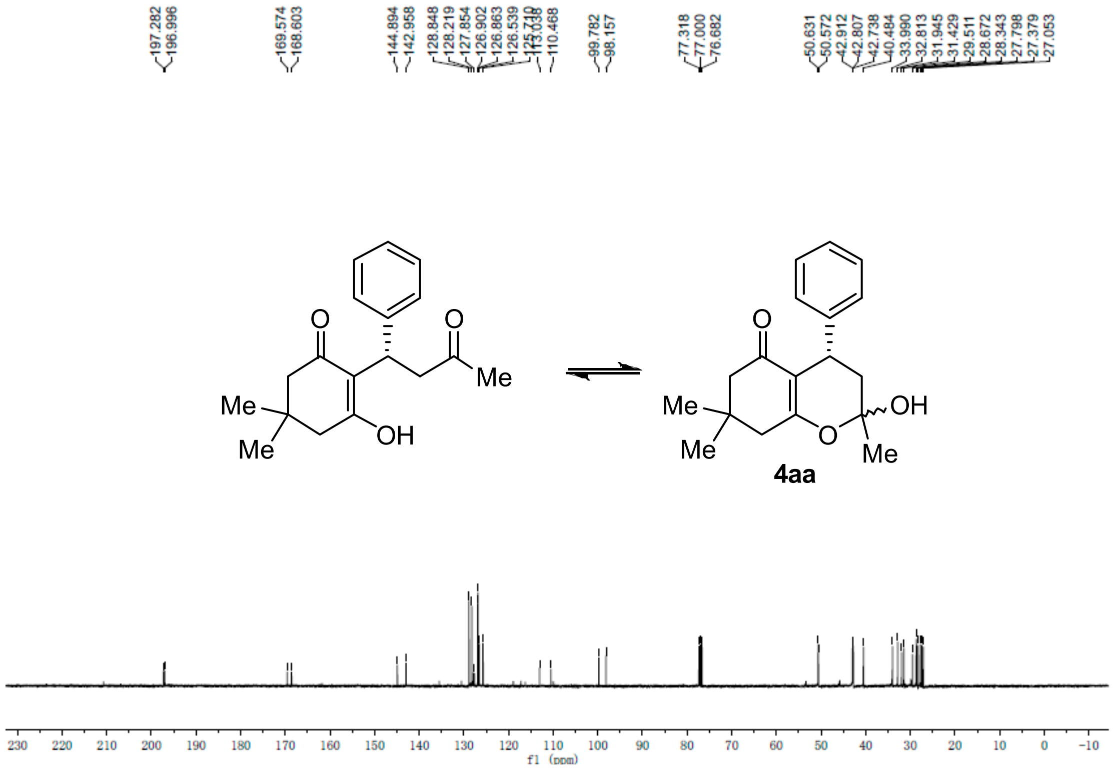

Chromatogram

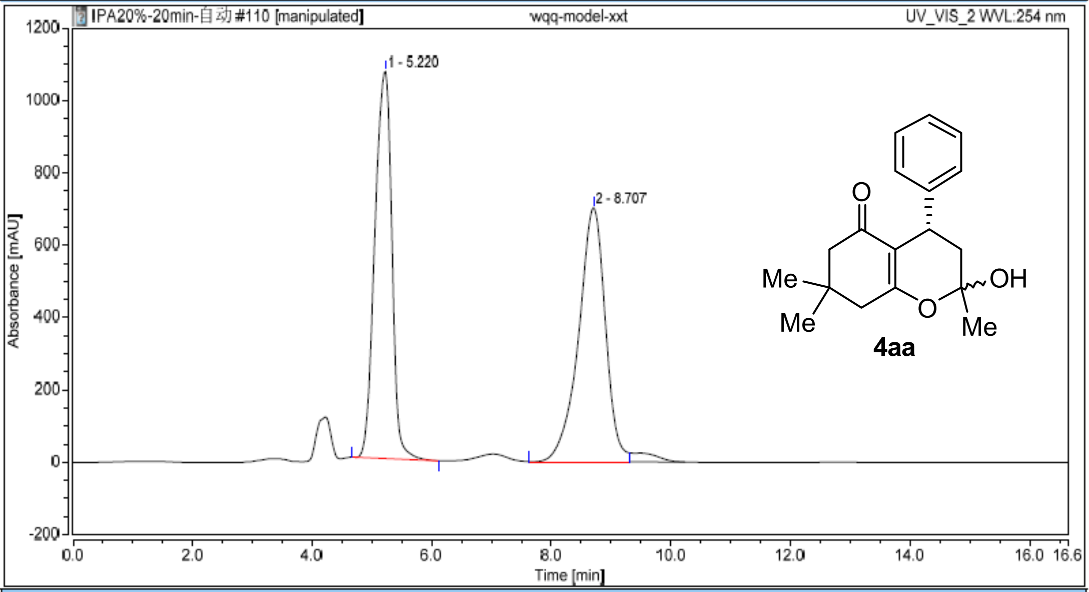

Integration Results

No.	Peak Name	Retention Time<br>min	Area<br>mAU*min	Height<br>mAU	Relative Area<br>%	Relative Height<br>%	Amount<br>n.a.
1		5.220	352.641	1071.092	49.14	60.40	n.a.
2		8.707	364.980	702.278	50.86	39.60	n.a.
Total:			**717.621**	**1773.370**	**100.00**	**100.00**	

Chromatogram

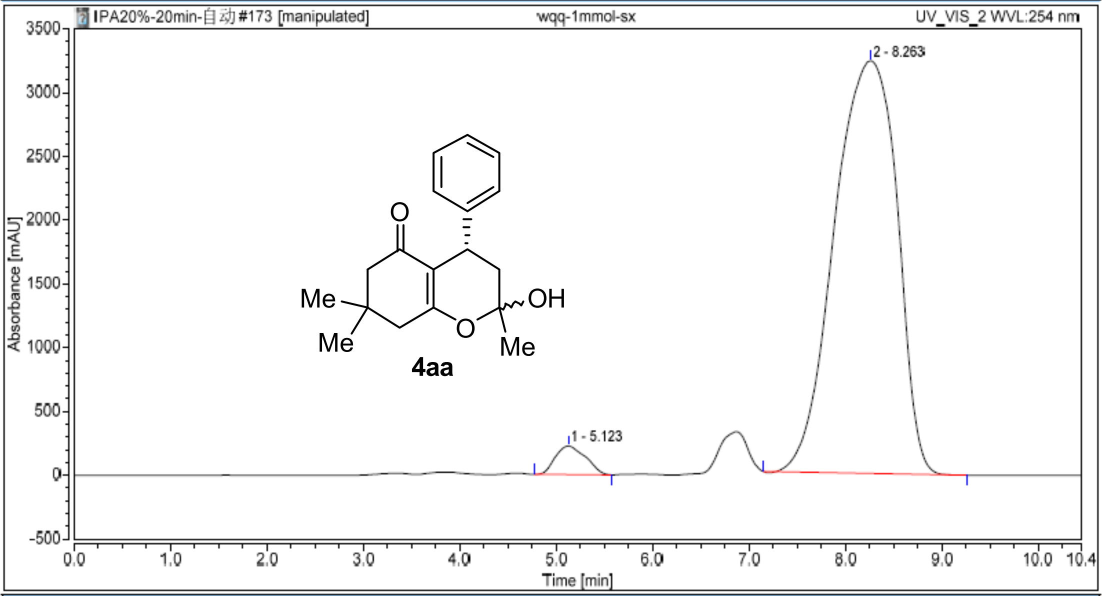

Integration Results

No.	Peak Name	Retention Time<br>min	Area<br>mAU*min	Height<br>mAU	Relative Area<br>%	Relative Height<br>%	Amount<br>n.a.
1		5.123	81.402	223.787	3.22	6.47	n.a.
2		8.263	2443.790	3235.832	96.78	93.53	n.a.
Total:			**2525.192**	**3459.619**	**100.00**	**100.00**	

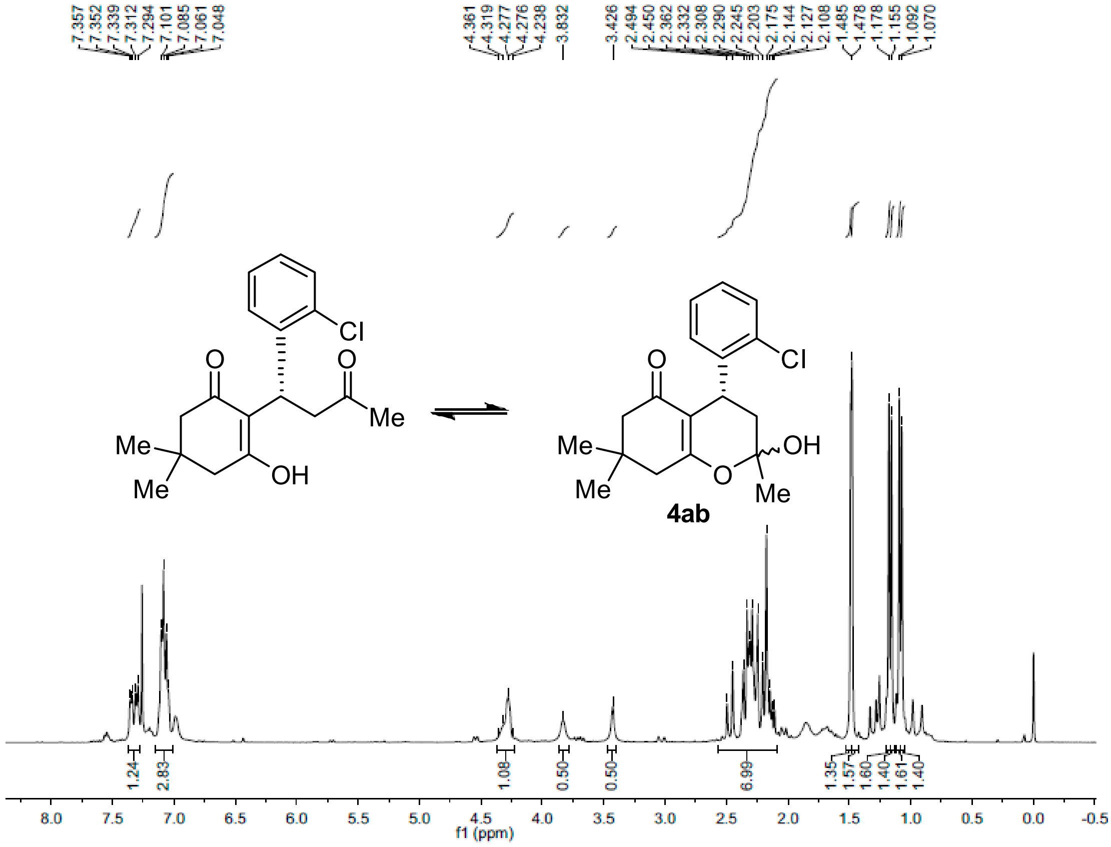
7.357
7.352
7.339
7.312
7.294
7.101
7.085
7.061
7.048
4.361
4.319
4.277
4.276
4.238
3.832
3.426
2.494
2.450
2.362
2.332
2.308
2.290
2.245
2.203
2.175
2.144
2.127
2.108
1.485
1.478
1.178
1.155
1.092
1.070
Cl
O
O
Me
Me
Me
OH
Cl
O
Me
Me
O
OH
Me
4ab
1.24
2.83
1.08
0.50
0.50
6.99
1.35
1.57
1.60
1.40
1.61
1.40
f1 (ppm)

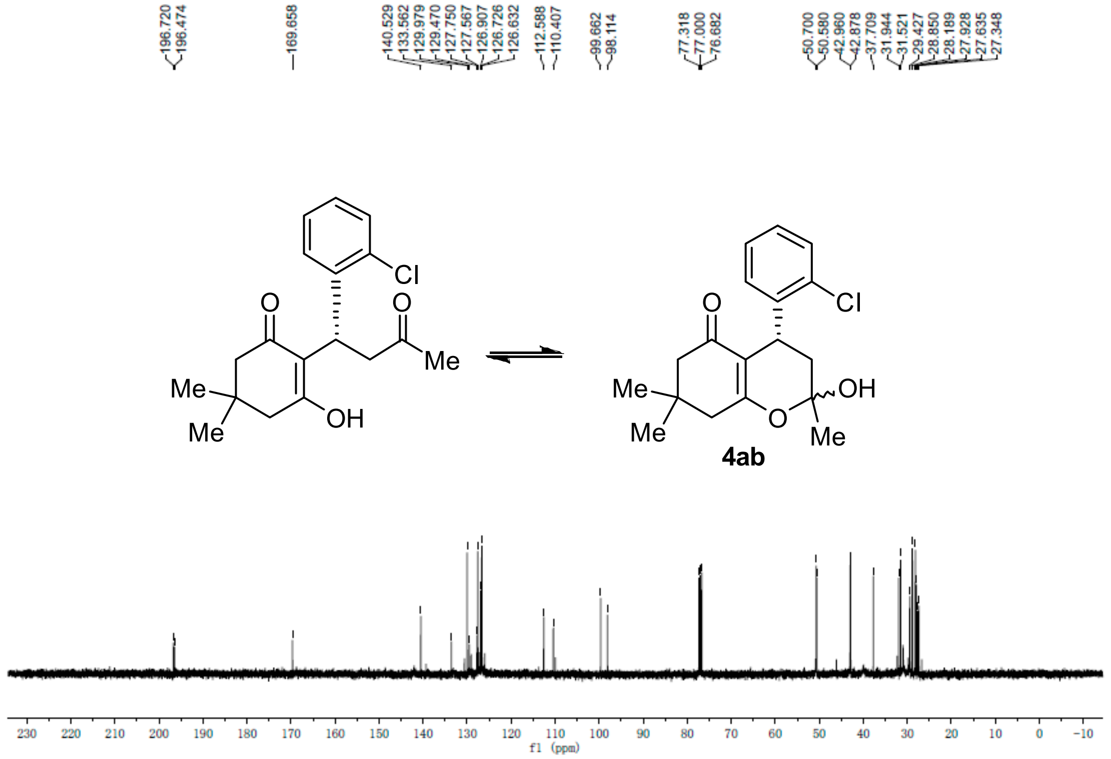
196.720
196.474
169.658
140.529
133.562
129.979
129.470
127.750
127.567
126.907
126.726
126.632
112.588
110.407
99.662
98.114
77.318
77.000
76.682
50.700
50.580
42.960
42.878
37.709
31.944
31.521
29.427
28.850
28.189
27.928
27.635
27.348
Cl
O
O
Me
Me
Me
OH
Cl
O
Me
Me
O
OH
Me
4ab
f1 (ppm)

Chromatogram

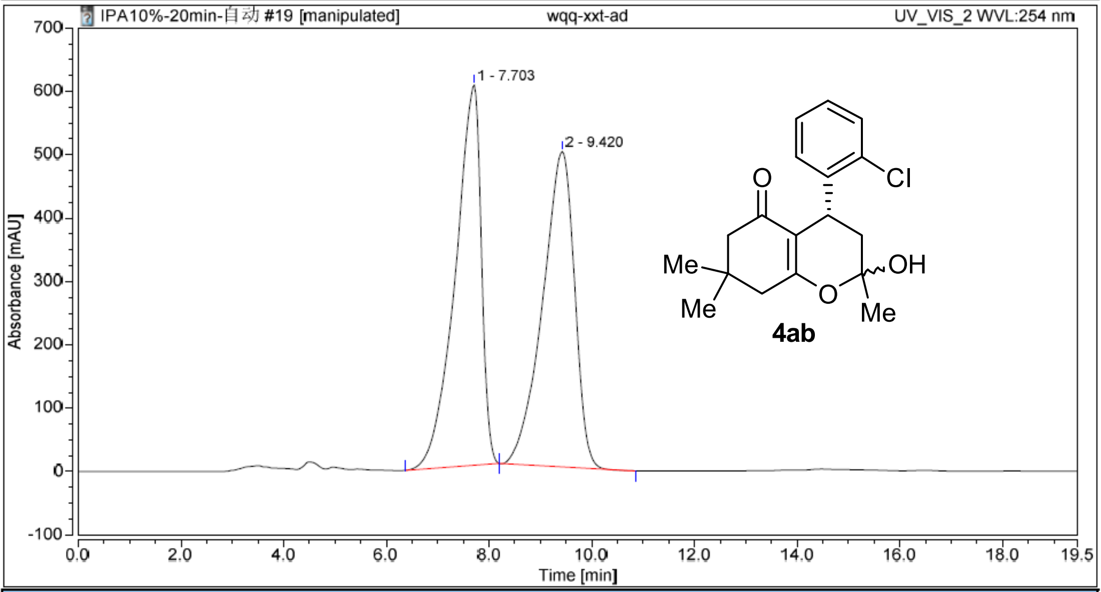

Integration Results

No.	Peak Name	Retention Time min	Area mAU*min	Height mAU	Relative Area %	Relative Height %	Amount n.a.
1		7.703	352.243	600.488	49.56	54.67	n.a.
2		9.420	358.569	497.929	50.44	45.33	n.a.
**Total:**			**710.811**	**1098.417**	**100.00**	**100.00**	

Chromatogram

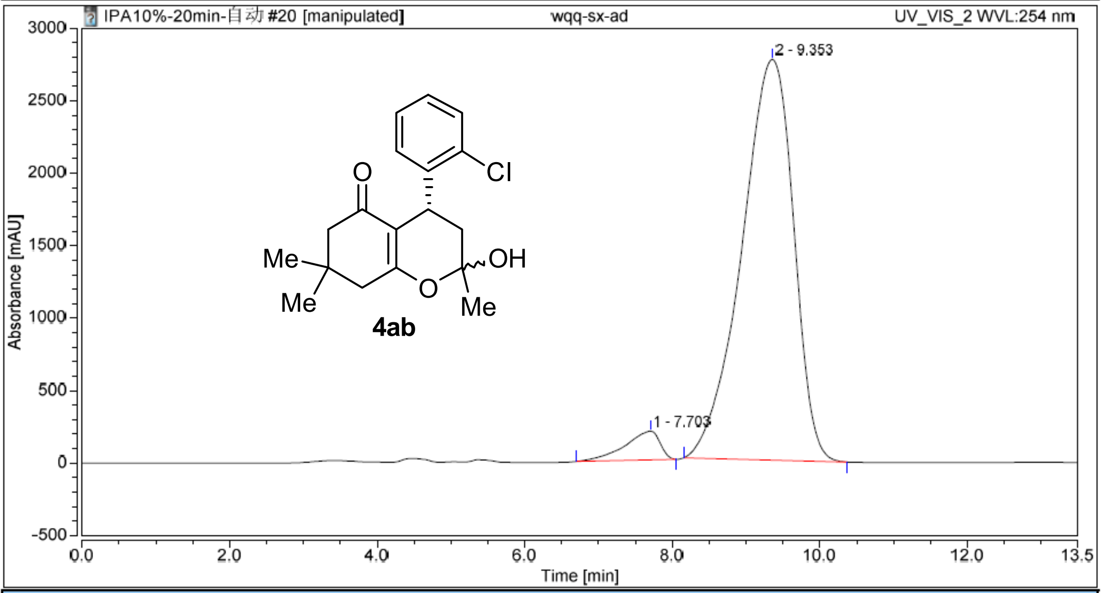

Integration Results

No.	Peak Name	Retention Time min	Area mAU*min	Height mAU	Relative Area %	Relative Height %	Amount n.a.
1		7.703	109.008	198.556	4.61	6.69	n.a.
2		9.353	2253.872	2767.411	95.39	93.31	n.a.
**Total:**			**2362.880**	**2965.967**	**100.00**	**100.00**	

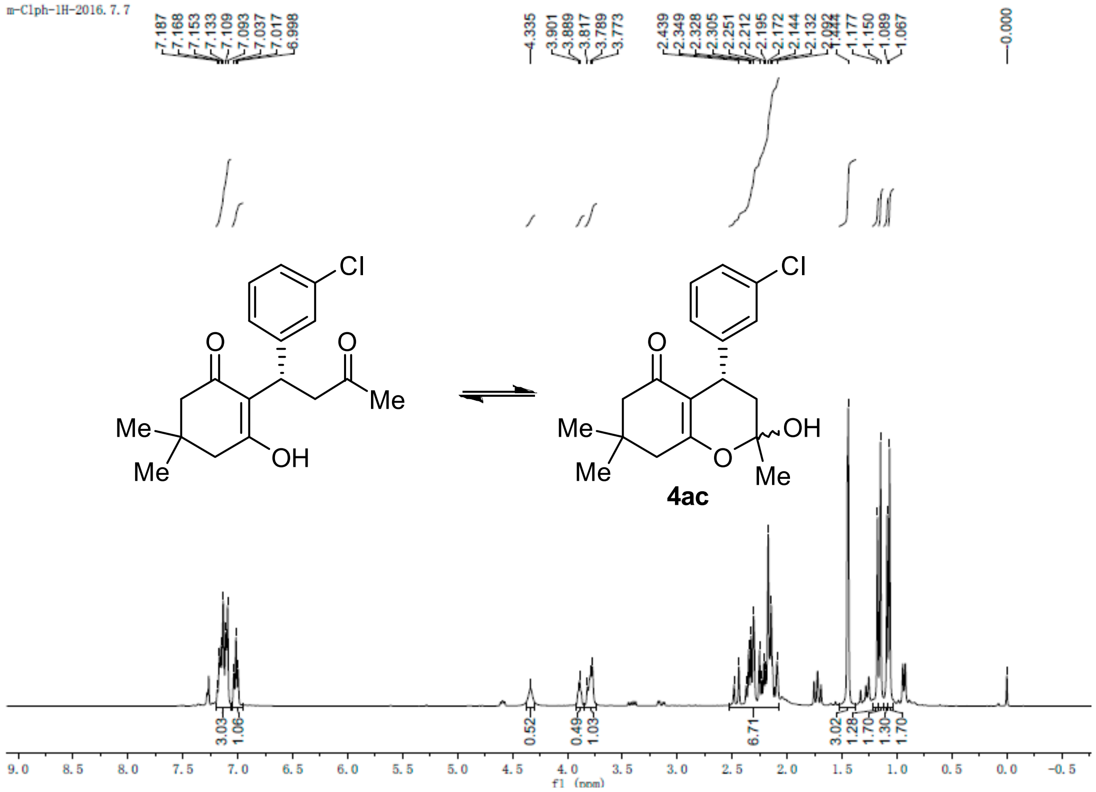
m-Clph-1H-2016. 7. 7
Cl
O
O
Me
Me
Me
OH
Cl
O
OH
Me
Me
O
Me
4ac
f1 (ppm)

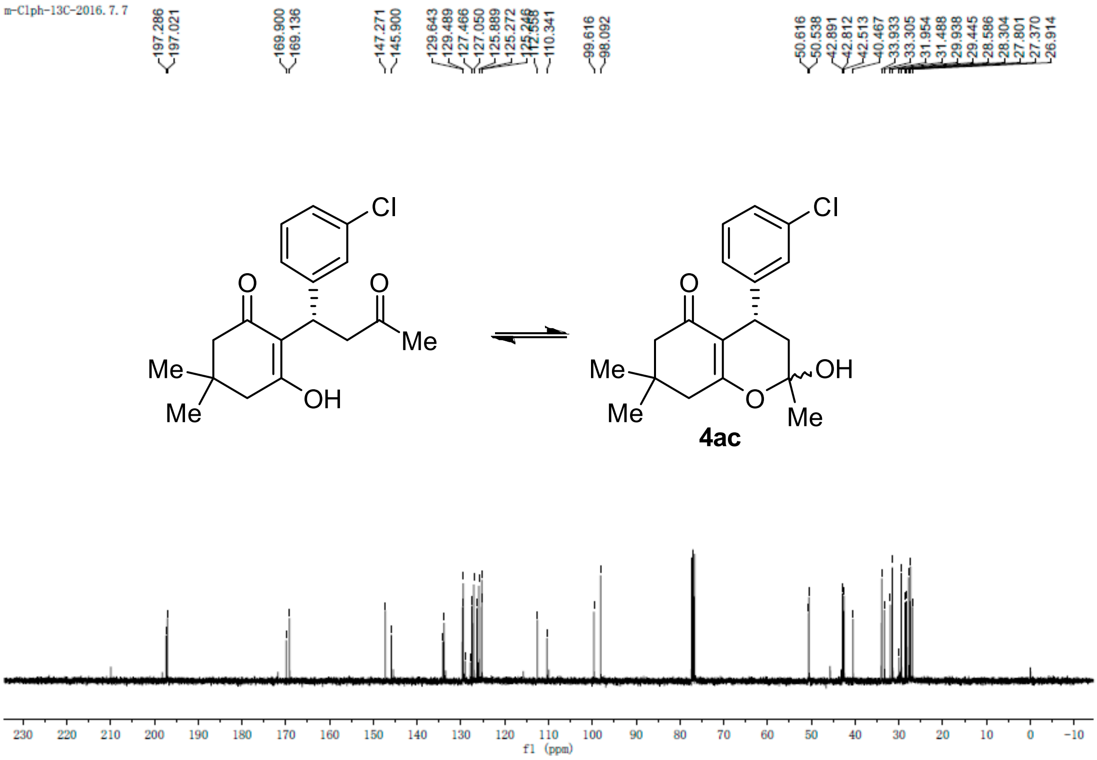
m-Clph-13C-2016. 7. 7
Cl
O
O
Me
Me
Me
OH
Cl
O
OH
Me
Me
O
Me
4ac
f1 (ppm)

Chromatogram

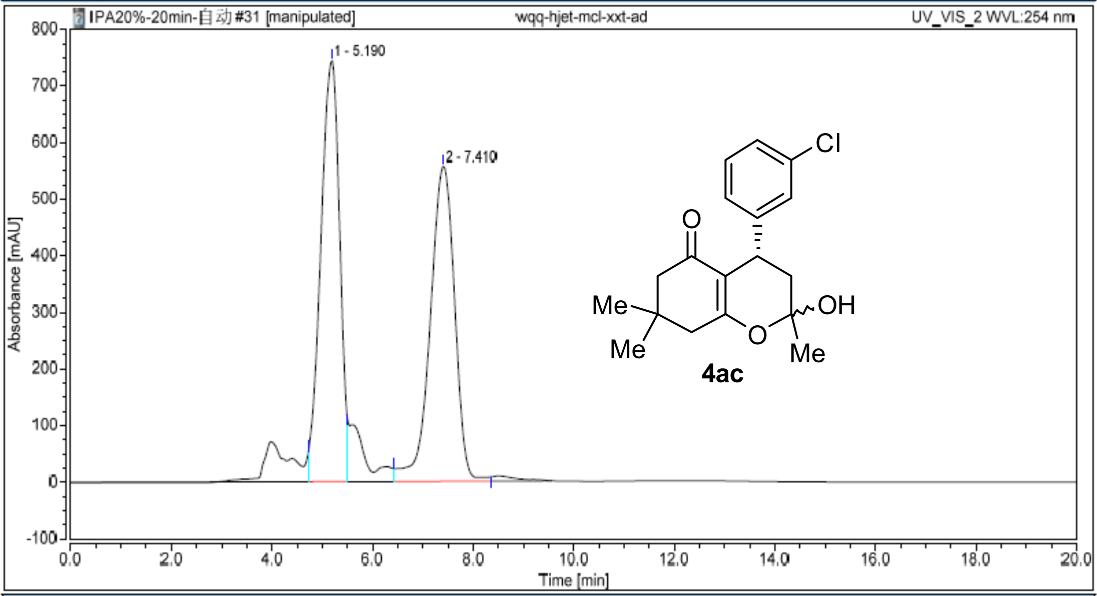

Integration Results

No.	Peak Name	Retention Time min	Area mAU*min	Height mAU	Relative Area %	Relative Height %	Amount n.a.
1		5.190	321.213	743.667	50.58	57.20	n.a.
2		7.410	313.884	556.348	49.42	42.80	n.a.
Total:			635.097	1300.015	100.00	100.00	

Chromatogram

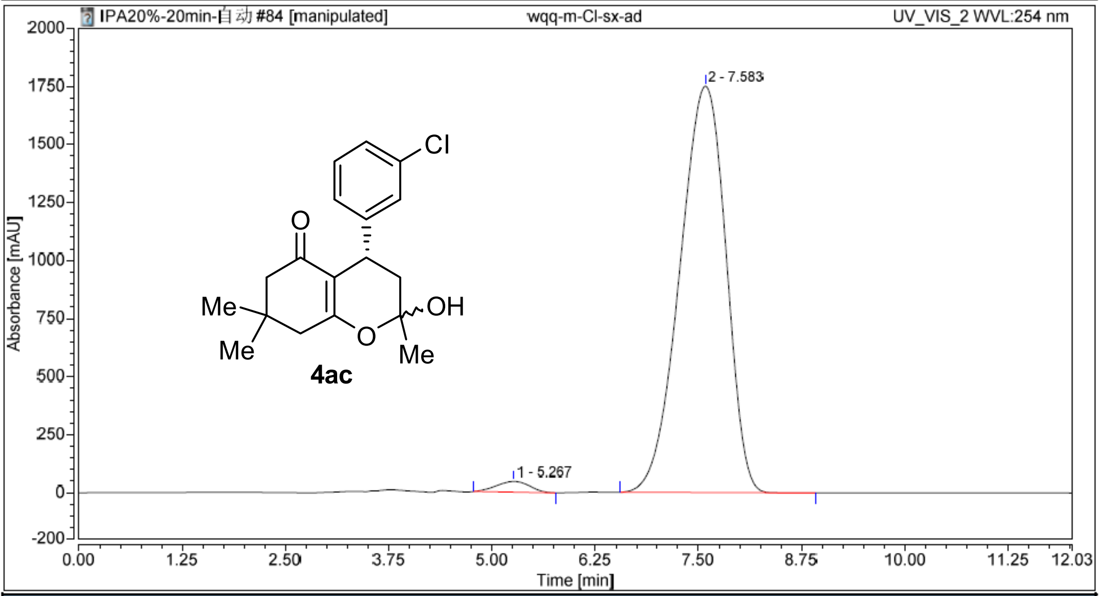

Integration Results

No.	Peak Name	Retention Time min	Area mAU*min	Height mAU	Relative Area %	Relative Height %	Amount n.a.
1		5.267	21.485	46.289	1.86	2.58	n.a.
2		7.583	1132.379	1750.489	98.14	97.42	n.a.
Total:			1153.864	1796.778	100.00	100.00	

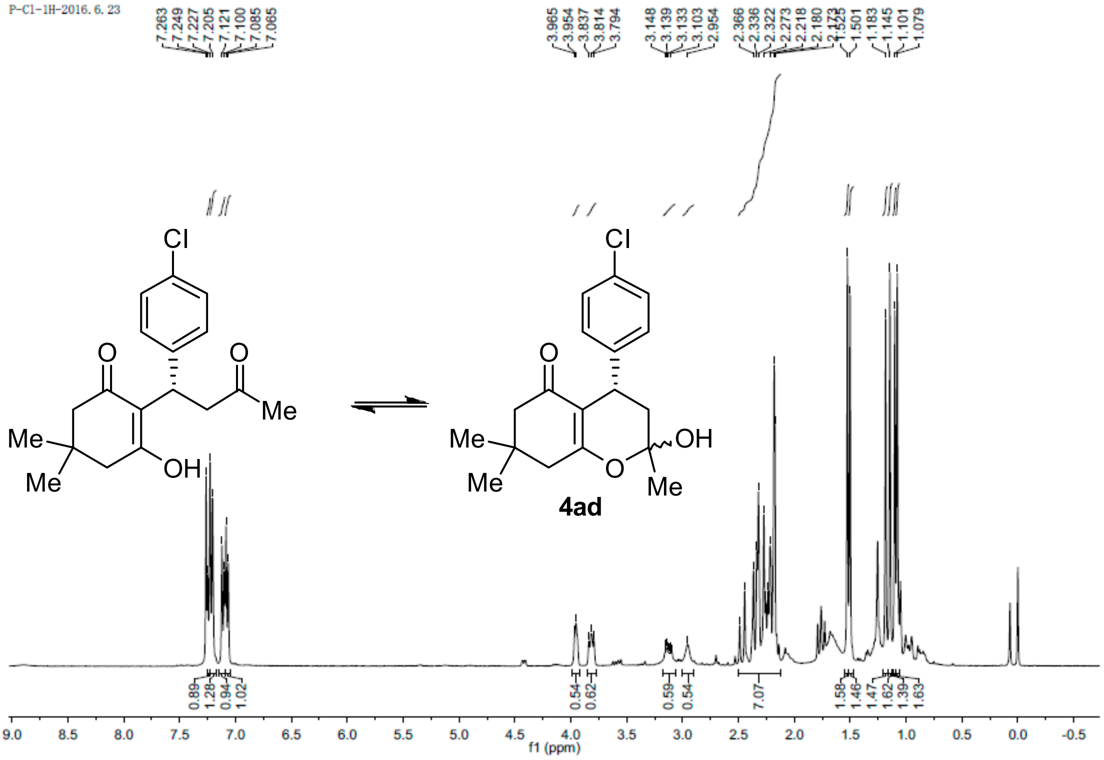
P-Cl-1H-2016. 6. 23
Cl
O
O
Me
Me
Me
OH
Cl
O
Me
Me
OH
O
Me
4ad
f1 (ppm)

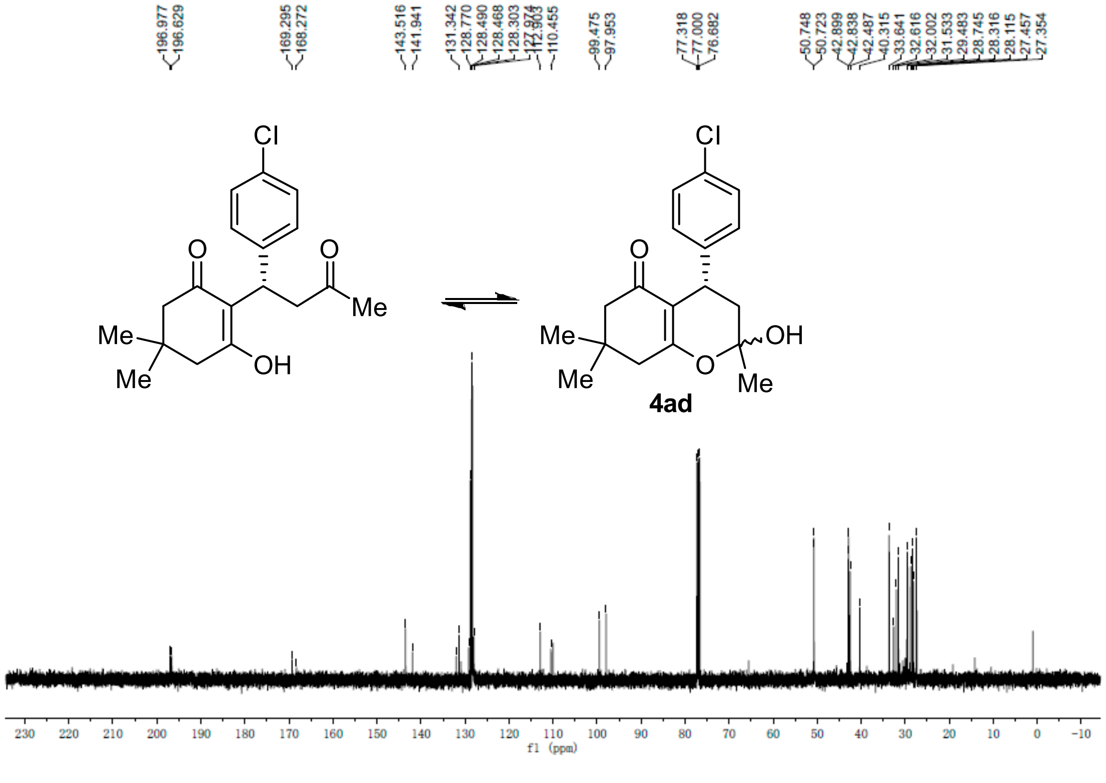
Cl
O
O
Me
Me
Me
OH
Cl
O
Me
Me
OH
O
Me
4ad
f1 (ppm)

Chromatogram

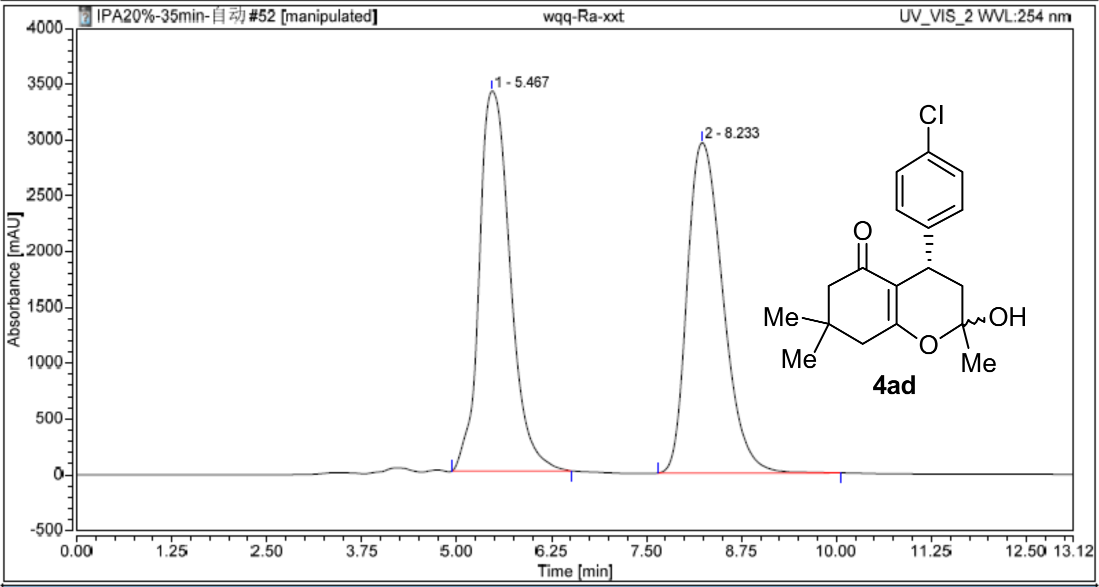

Integration Results

No.	Peak Name	Retention Time min	Area mAU*min	Height mAU	Relative Area %	Relative Height %	Amount n.a.
1		5.467	1613.364	3404.577	50.00	53.49	n.a.
2		8.233	1613.358	2960.608	50.00	46.51	n.a.
Total:			3226.722	6365.185	100.00	100.00	

Chromatogram

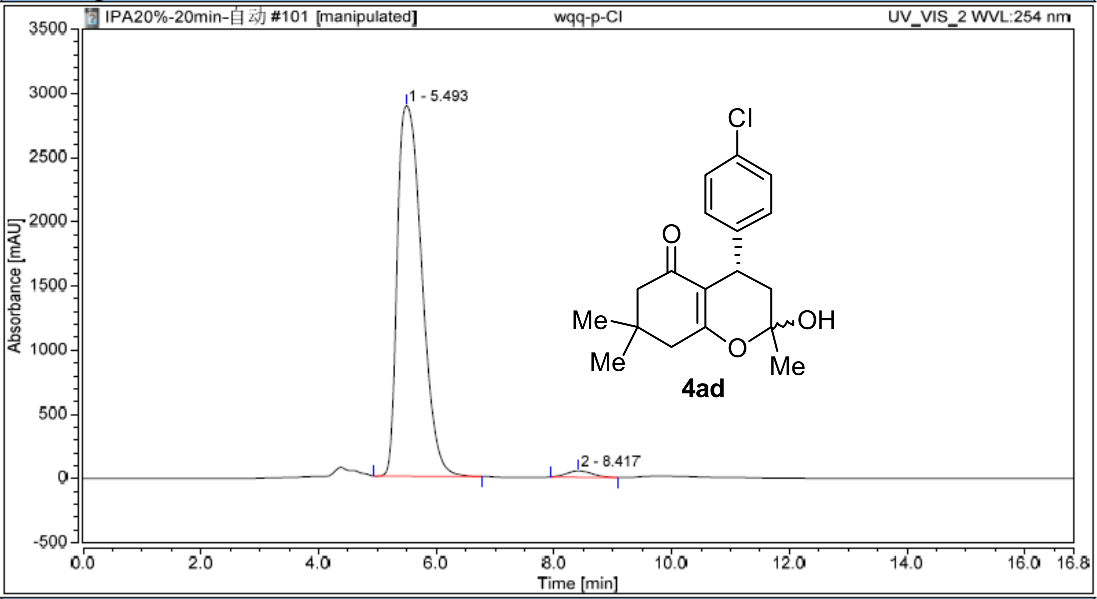

Integration Results

No.	Peak Name	Retention Time min	Area mAU*min	Height mAU	Relative Area %	Relative Height %	Amount n.a.
1		5.493	1389.584	2887.729	98.27	98.35	n.a.
2		8.417	24.408	48.518	1.73	1.65	n.a.
Total:			1413.992	2936.248	100.00	100.00	

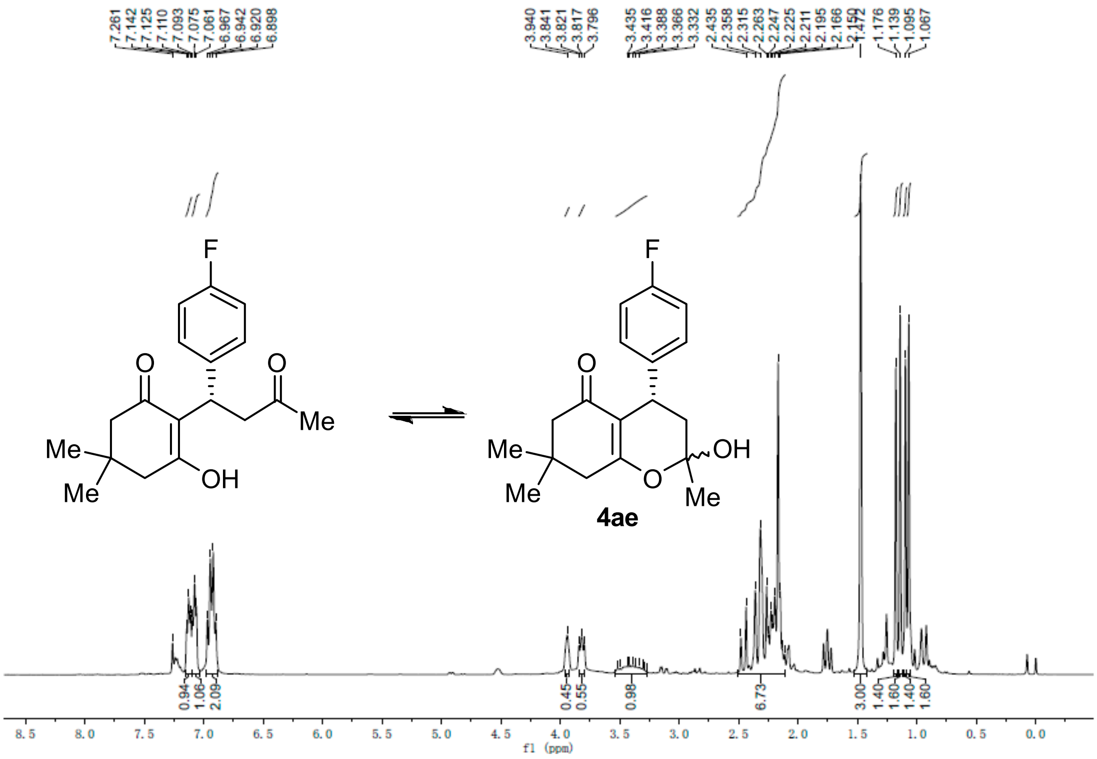

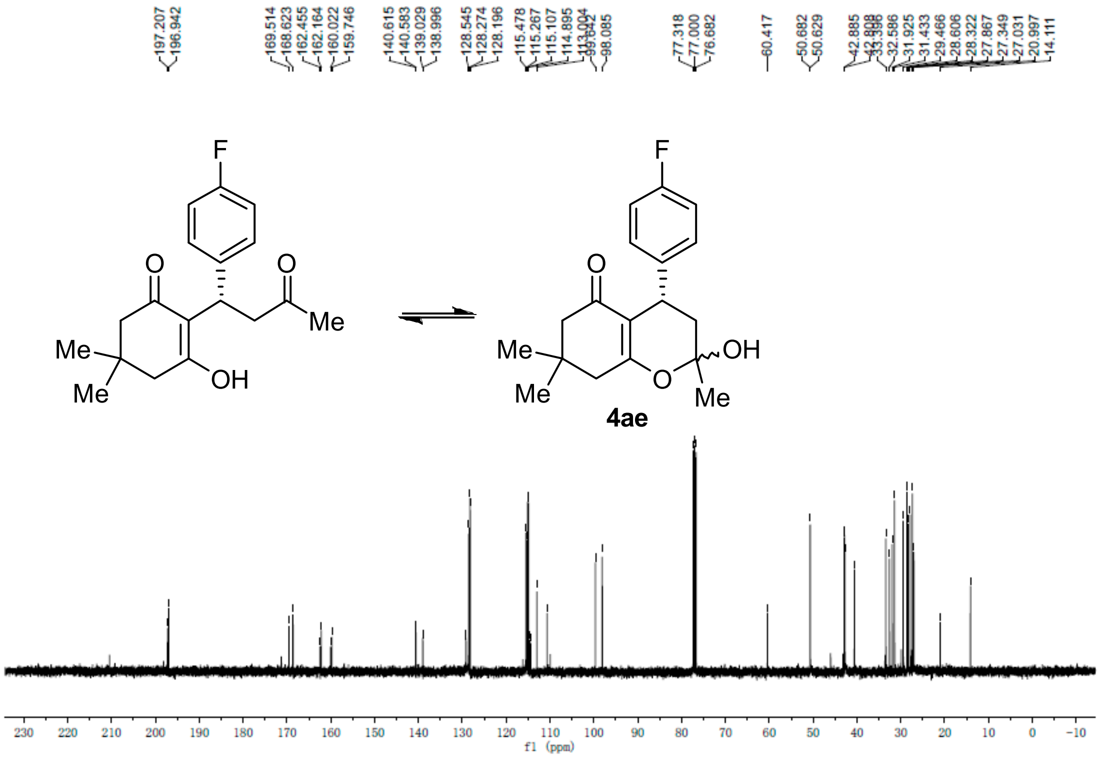

Chromatogram

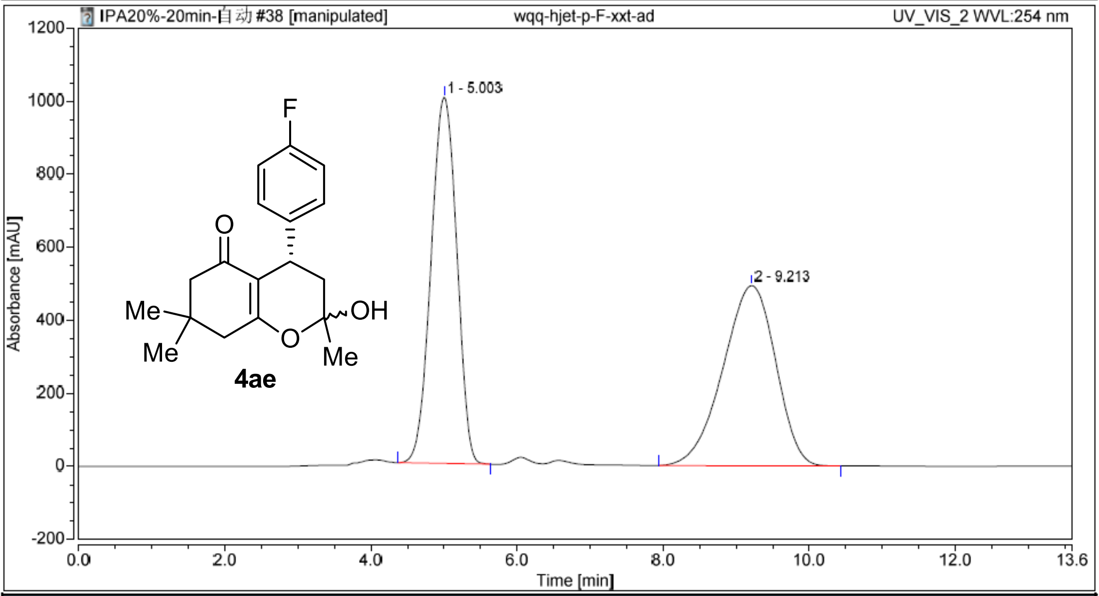

Integration Results

No.	Peak Name	Retention Time min	Area mAU*min	Height mAU	Relative Area %	Relative Height %	Amount n.a.
1		5.003	409.288	1003.007	49.83	67.03	n.a.
2		9.213	412.096	493.402	50.17	32.97	n.a.
**Total:**			**821.384**	**1496.409**	**100.00**	**100.00**	

Chromatogram

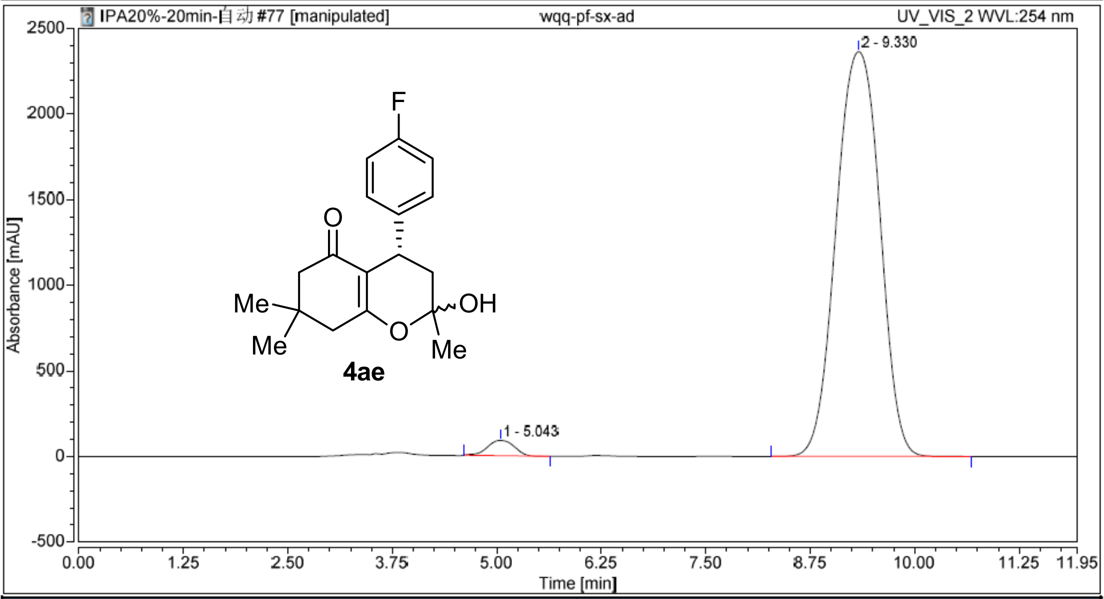

Integration Results

No.	Peak Name	Retention Time min	Area mAU*min	Height mAU	Relative Area %	Relative Height %	Amount n.a.
1		5.043	32.869	89.146	2.24	3.63	n.a.
2		9.330	1434.374	2364.527	97.76	96.37	n.a.
**Total:**			**1467.243**	**2453.673**	**100.00**	**100.00**	

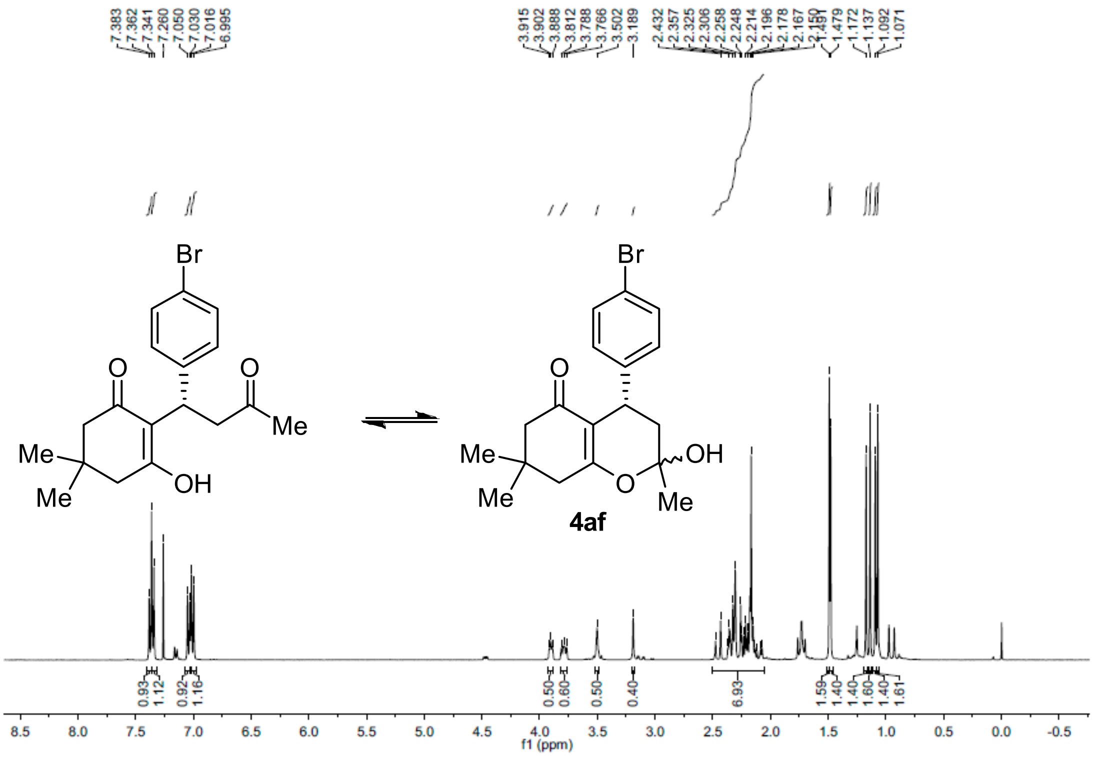
4af
f1 (ppm)

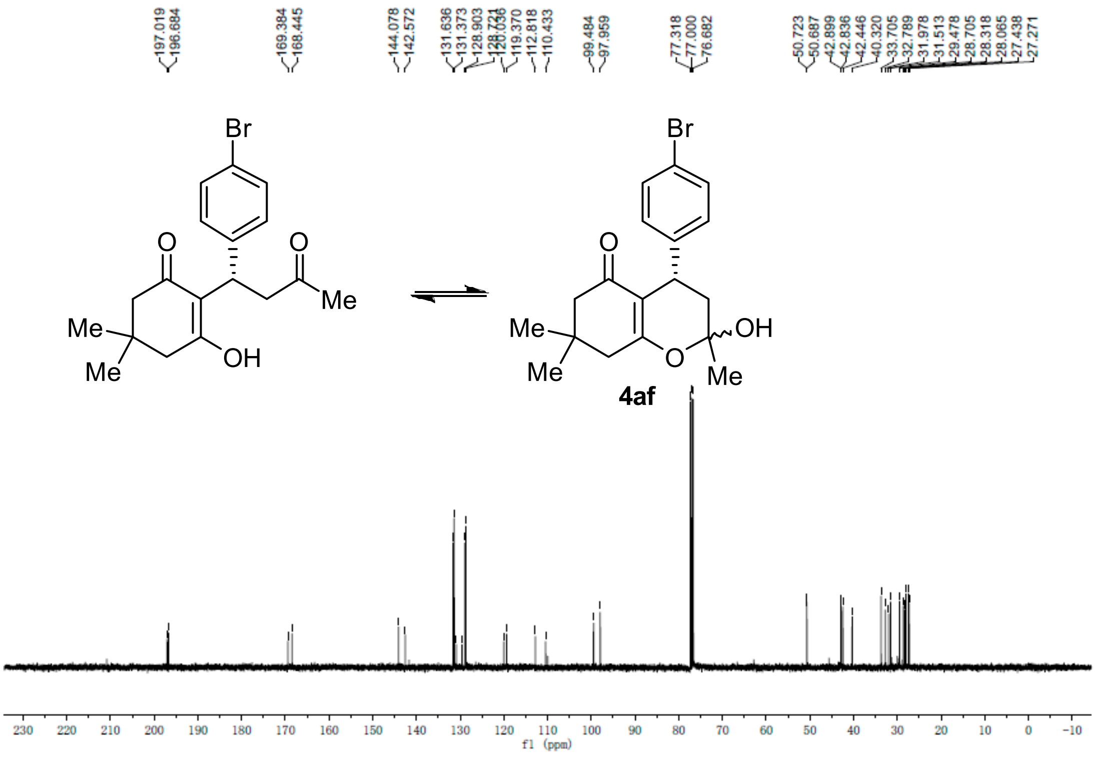
4af
f1 (ppm)

Chromatogram

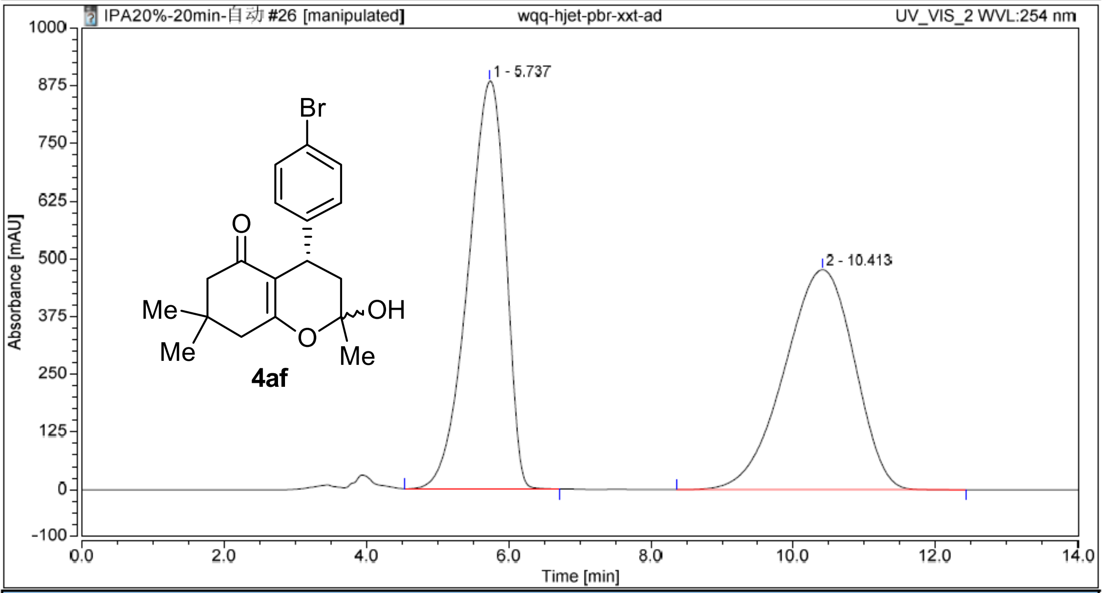

Integration Results

No.	Peak Name	Retention Time min	Area mAU*min	Height mAU	Relative Area %	Relative Height %	Amount n.a.
1		5.737	540.973	883.670	49.91	64.97	n.a.
2		10.413	543.026	476.361	50.09	35.03	n.a.
**Total:**			**1083.999**	**1360.031**	**100.00**	**100.00**	

Chromatogram

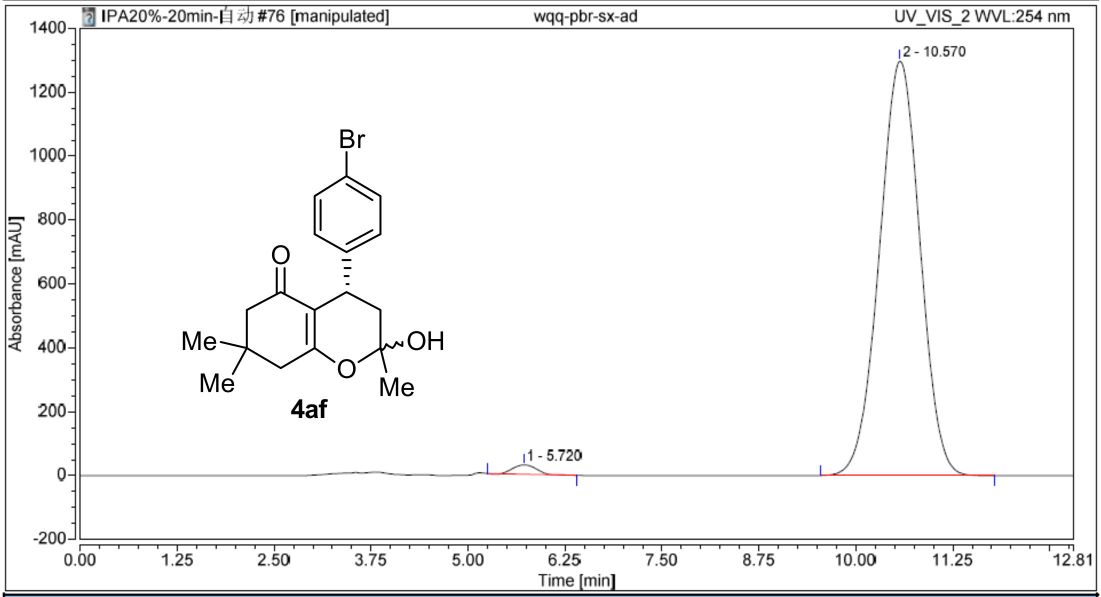

Integration Results

No.	Peak Name	Retention Time min	Area mAU*min	Height mAU	Relative Area %	Relative Height %	Amount n.a.
1		5.720	10.053	29.308	1.26	2.21	n.a.
2		10.570	787.526	1297.608	98.74	97.79	n.a.
**Total:**			**797.579**	**1326.916**	**100.00**	**100.00**	

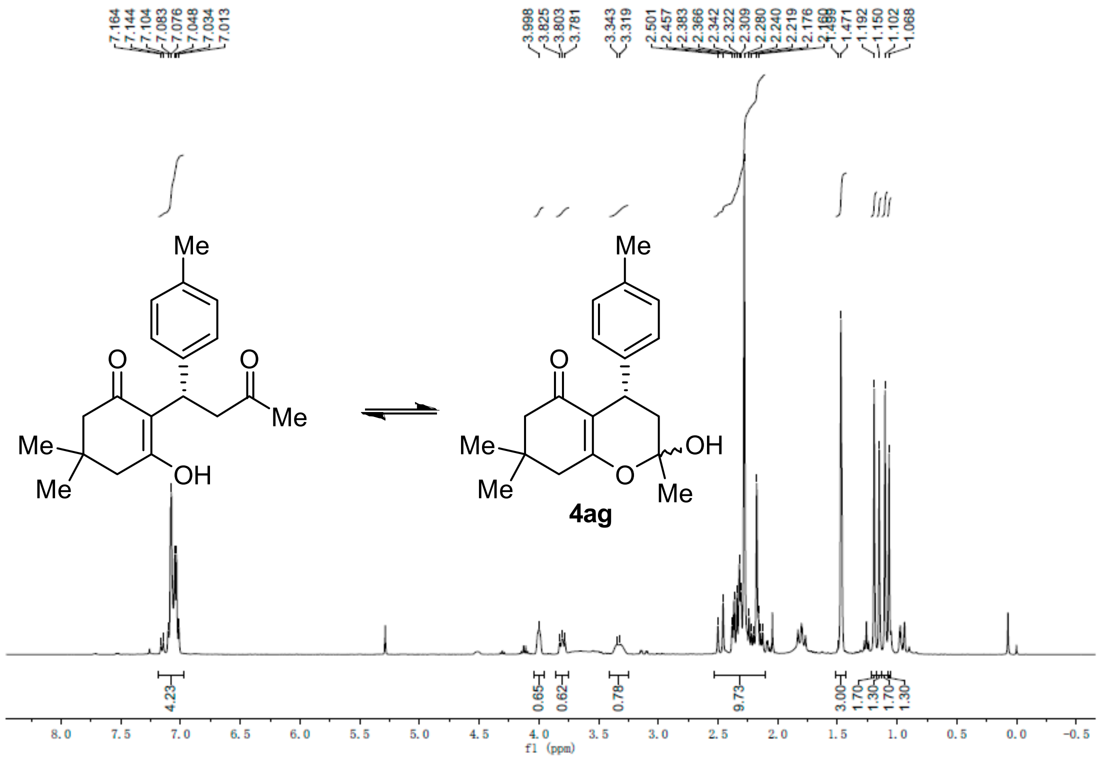

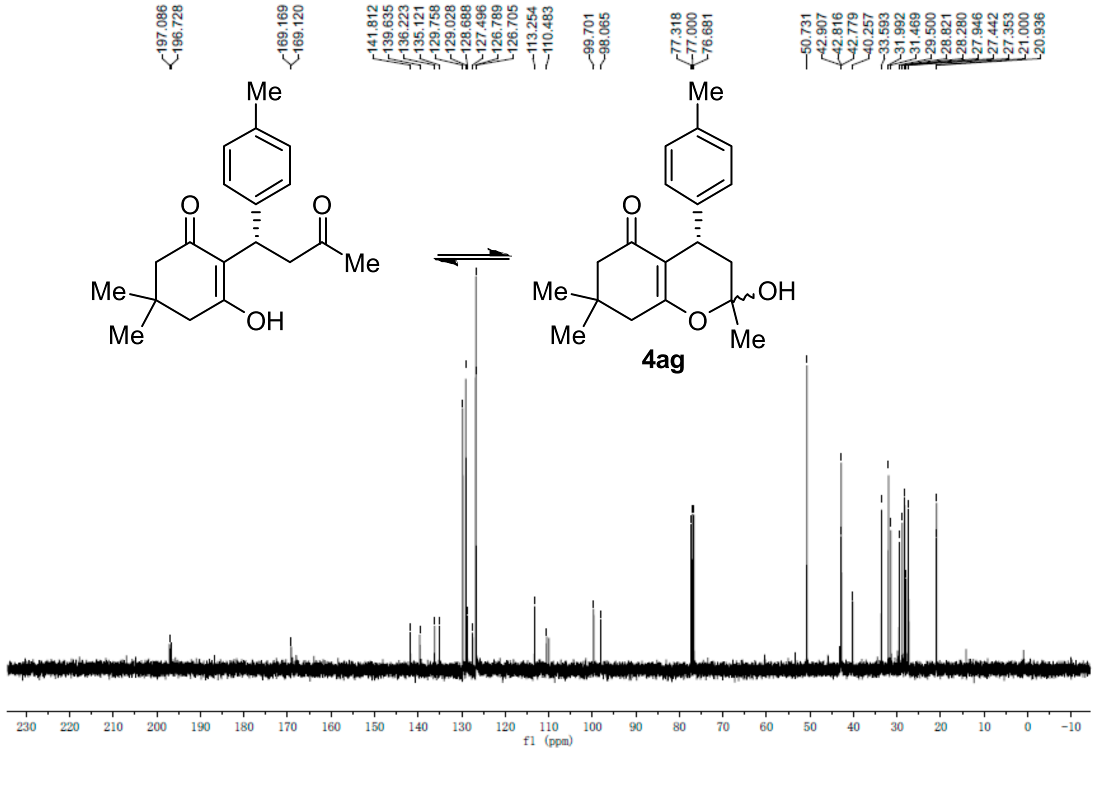

Chromatogram

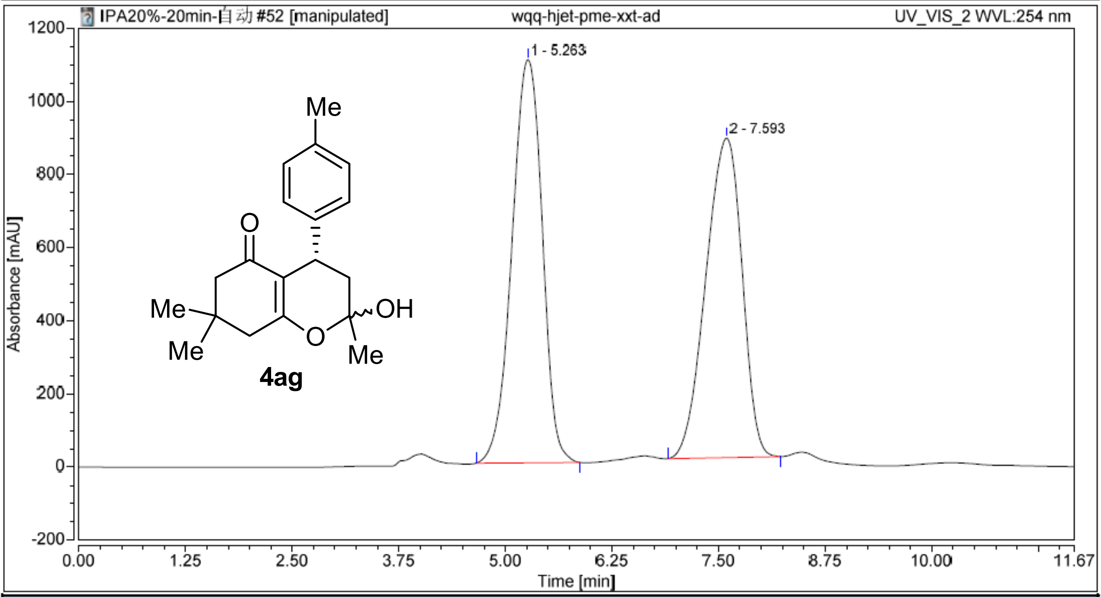

Integration Results

No.	Peak Name	Retention Time min	Area mAU*min	Height mAU	Relative Area %	Relative Height %	Amount n.a.
1		5.263	453.770	1103.175	51.35	55.80	n.a.
2		7.593	429.994	873.933	48.65	44.20	n.a.
**Total:**			**883.764**	**1977.109**	**100.00**	**100.00**	

Chromatogram

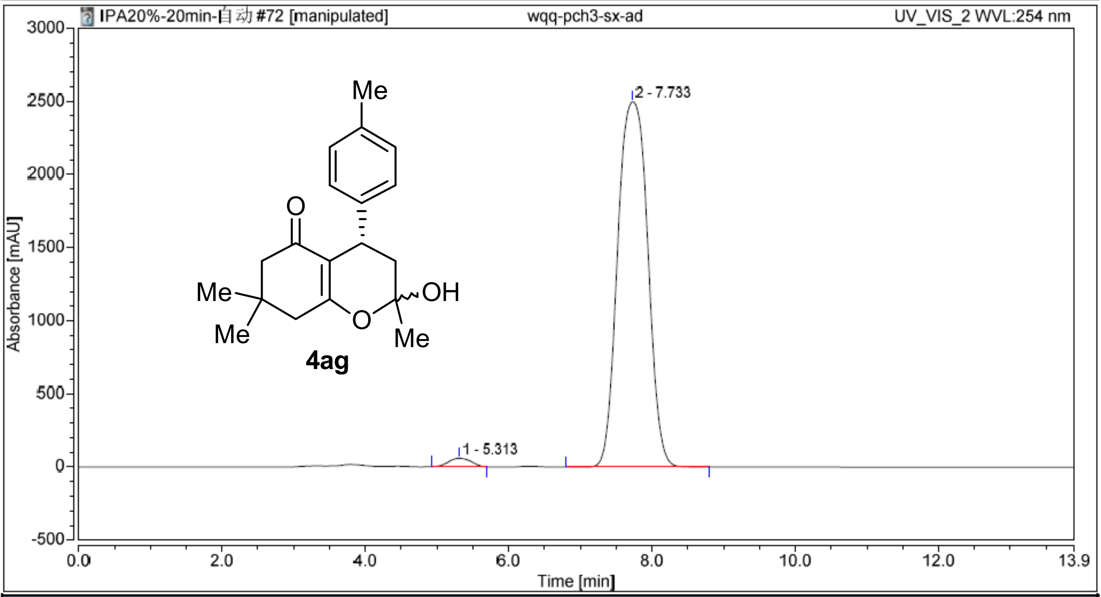

Integration Results

No.	Peak Name	Retention Time min	Area mAU*min	Height mAU	Relative Area %	Relative Height %	Amount n.a.
1		5.313	20.891	57.624	1.69	2.26	n.a.
2		7.733	1213.046	2497.261	98.31	97.74	n.a.
**Total:**			**1233.937**	**2554.885**	**100.00**	**100.00**	

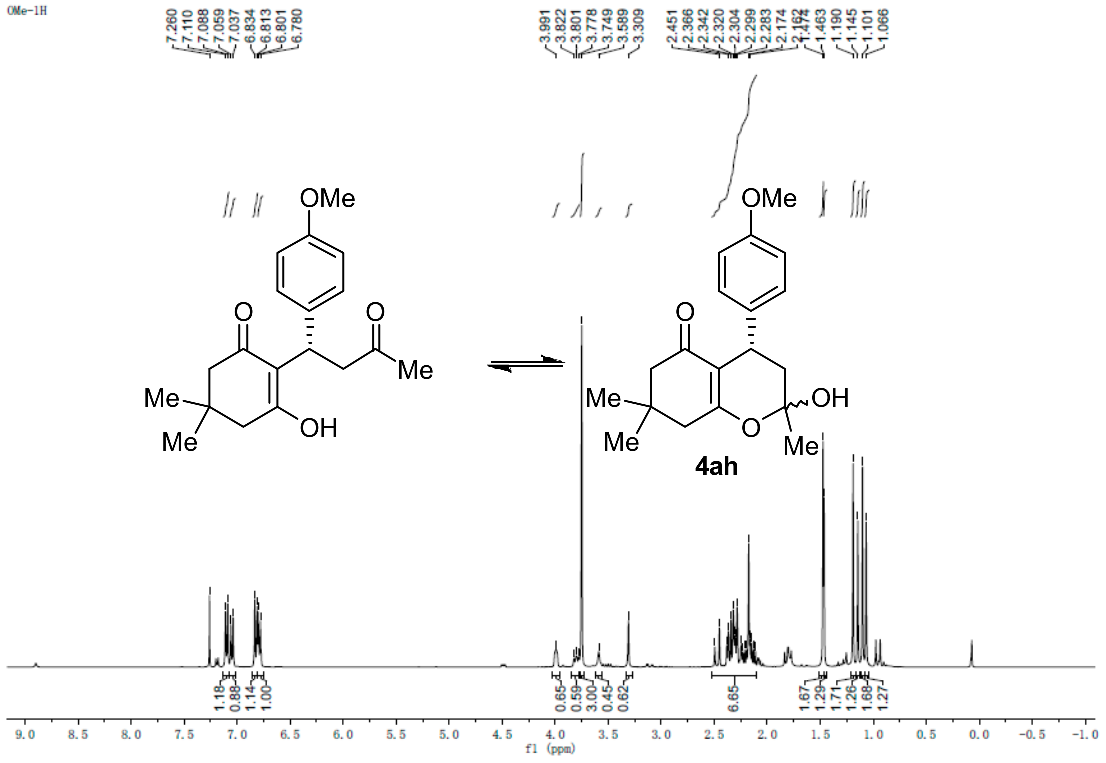

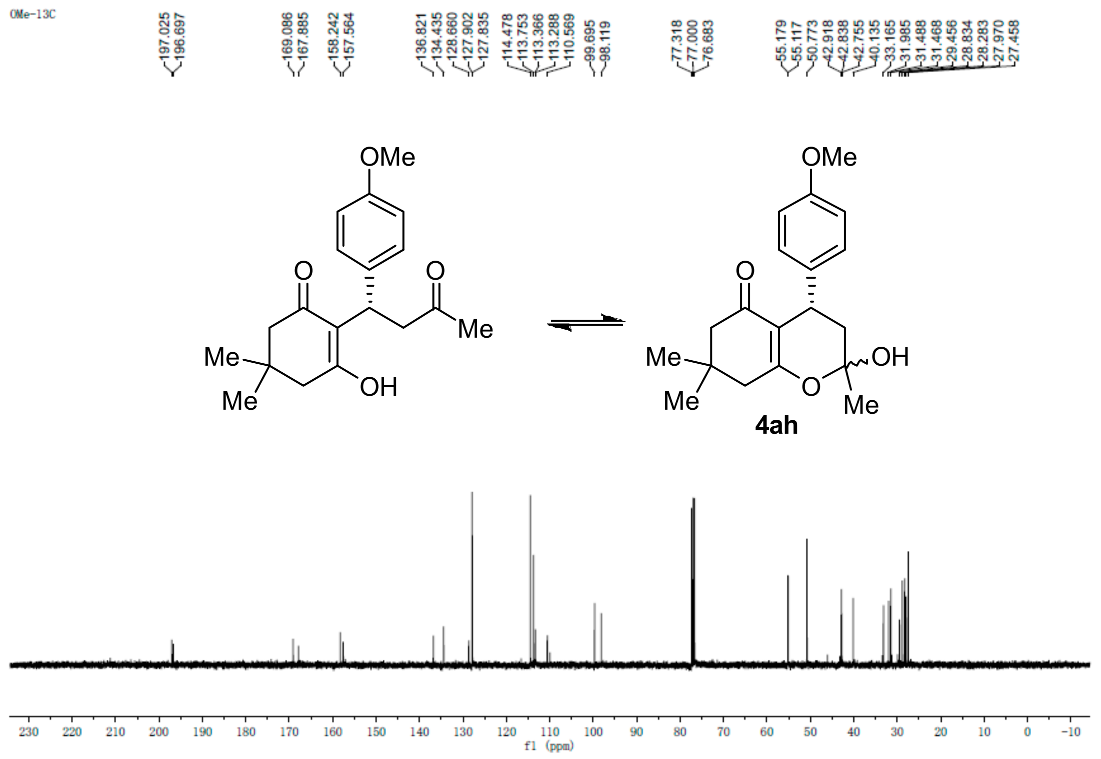

Chromatogram

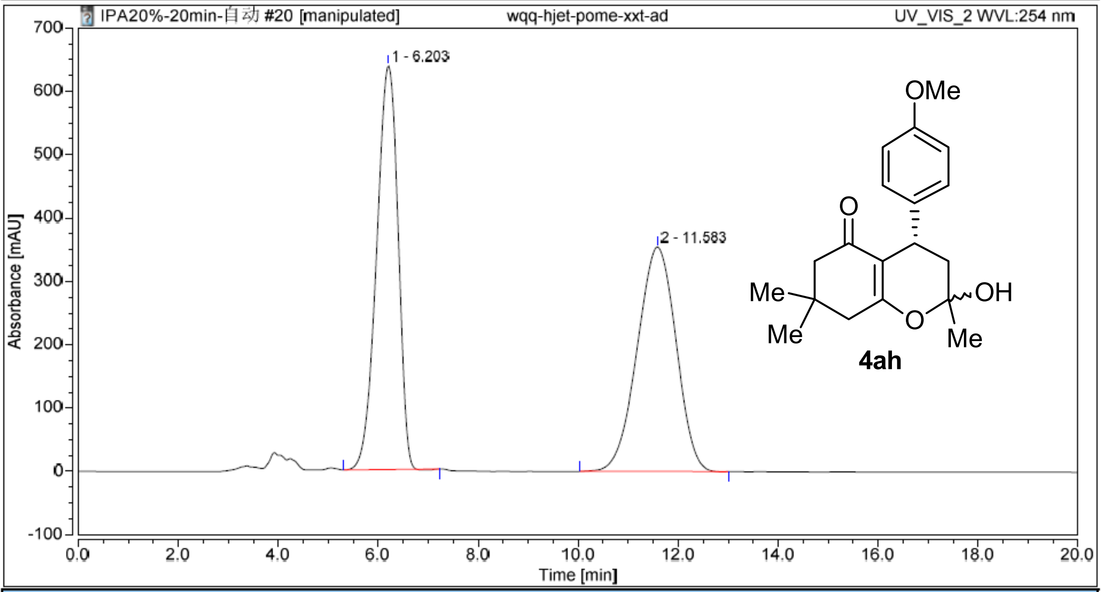

Integration Results

No.	Peak Name	Retention Time<br>min	Area<br>mAU*min	Height<br>mAU	Relative Area<br>%	Relative Height<br>%	Amount<br>n.a.
1		6.203	316.873	637.892	49.81	64.28	n.a.
2		11.583	319.328	354.509	50.19	35.72	n.a.
**Total:**			**636.201**	**992.401**	**100.00**	**100.00**	

Chromatogram

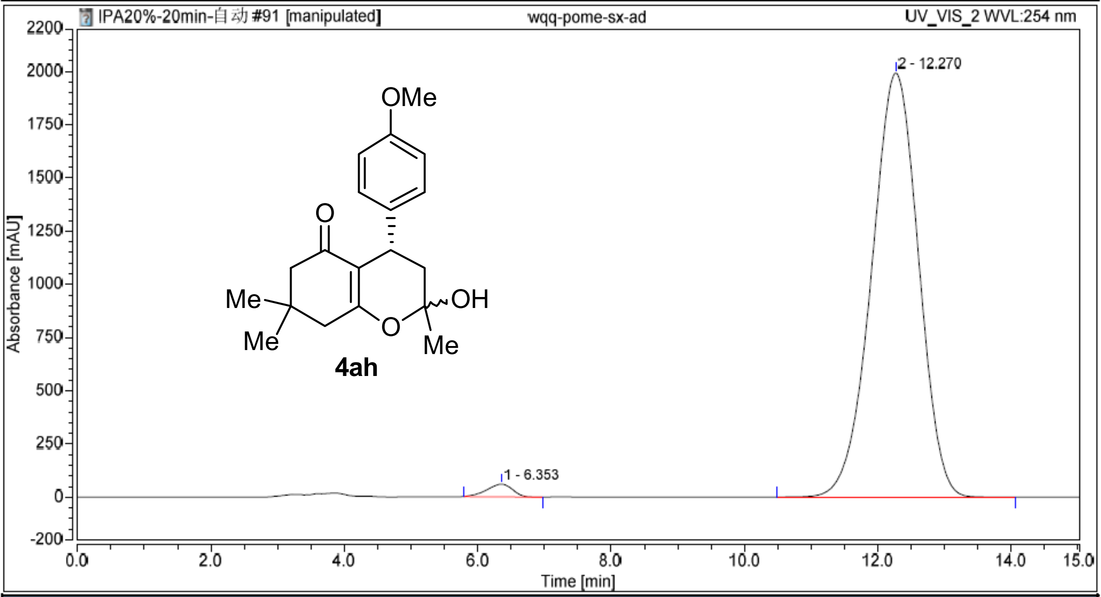

Integration Results

No.	Peak Name	Retention Time<br>min	Area<br>mAU*min	Height<br>mAU	Relative Area<br>%	Relative Height<br>%	Amount<br>n.a.
1		6.353	26.638	59.723	1.62	2.91	n.a.
2		12.270	1622.184	1992.391	98.38	97.09	n.a.
**Total:**			**1648.821**	**2052.114**	**100.00**	**100.00**	

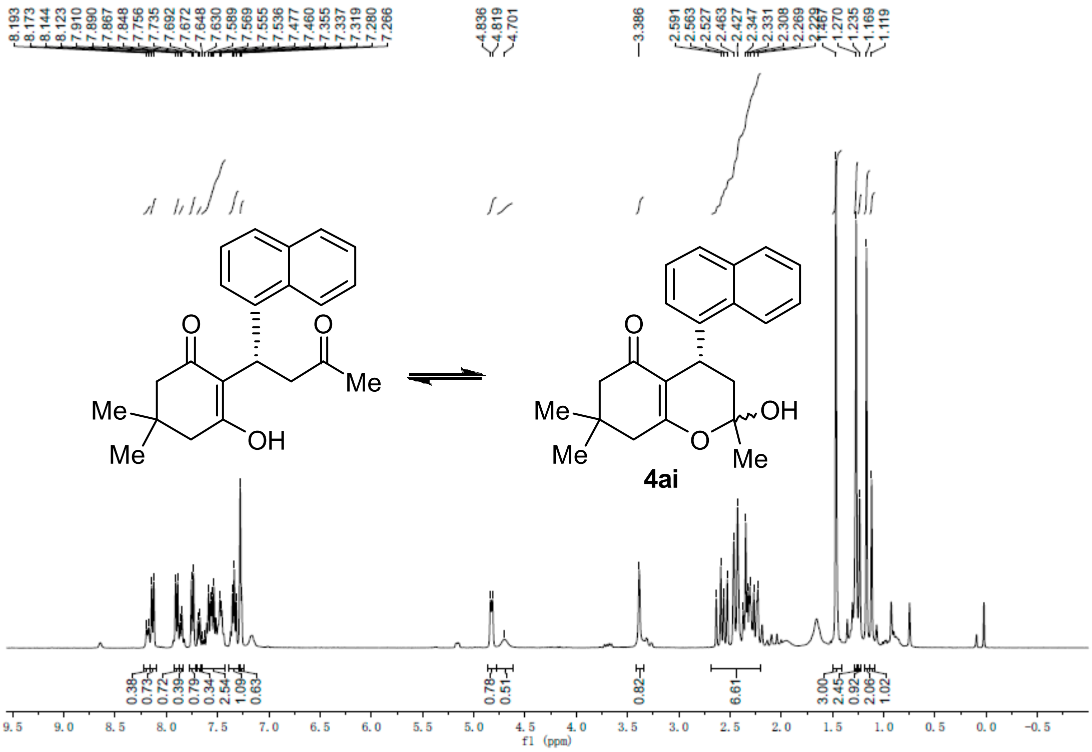

Me
Me
OH
O
O
Me
Me
Me
O
OH
O
Me
4ai
f1 (ppm)

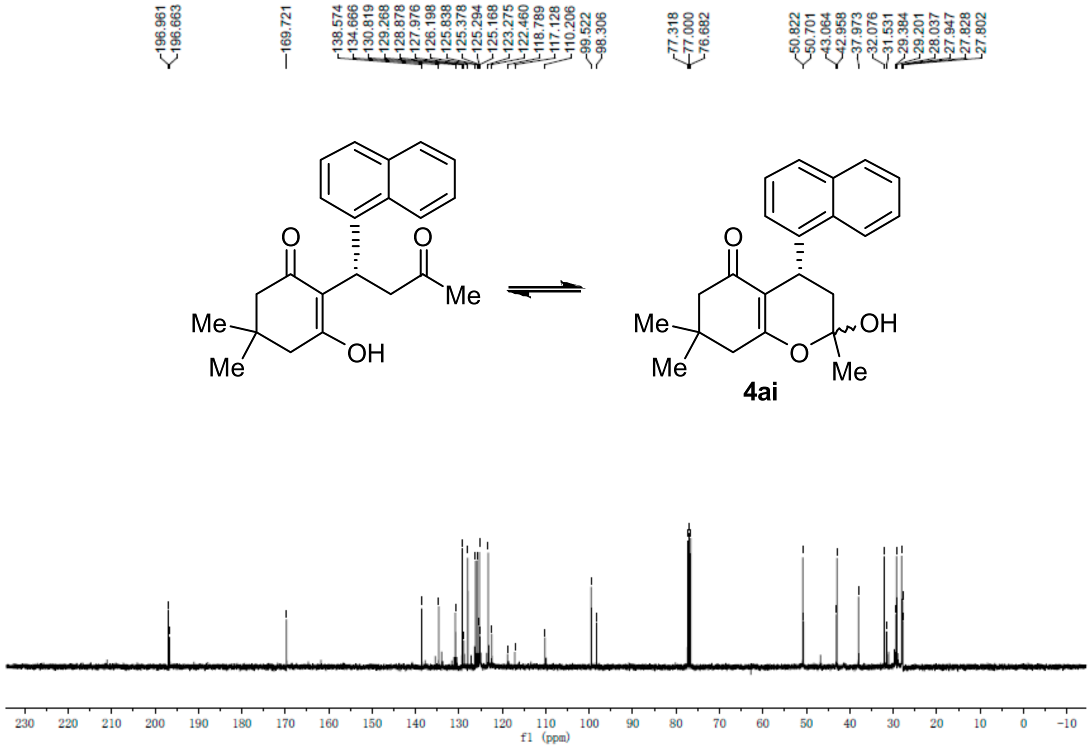

Me
Me
OH
O
O
Me
Me
Me
O
OH
O
Me
4ai
f1 (ppm)

Chromatogram

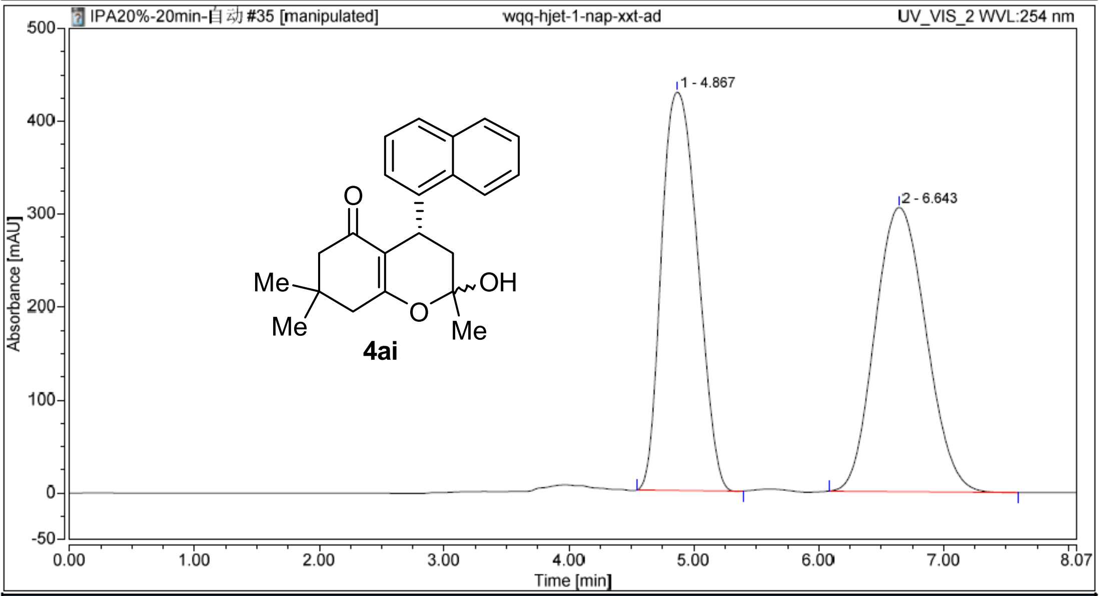

Integration Results

No.	Peak Name	Retention Time min	Area mAU*min	Height mAU	Relative Area %	Relative Height %	Amount n.a.
1		4.867	143.921	428.672	49.83	58.36	n.a.
2		6.643	144.892	305.805	50.17	41.64	n.a.
**Total:**			**288.813**	**734.477**	**100.00**	**100.00**	

Chromatogram

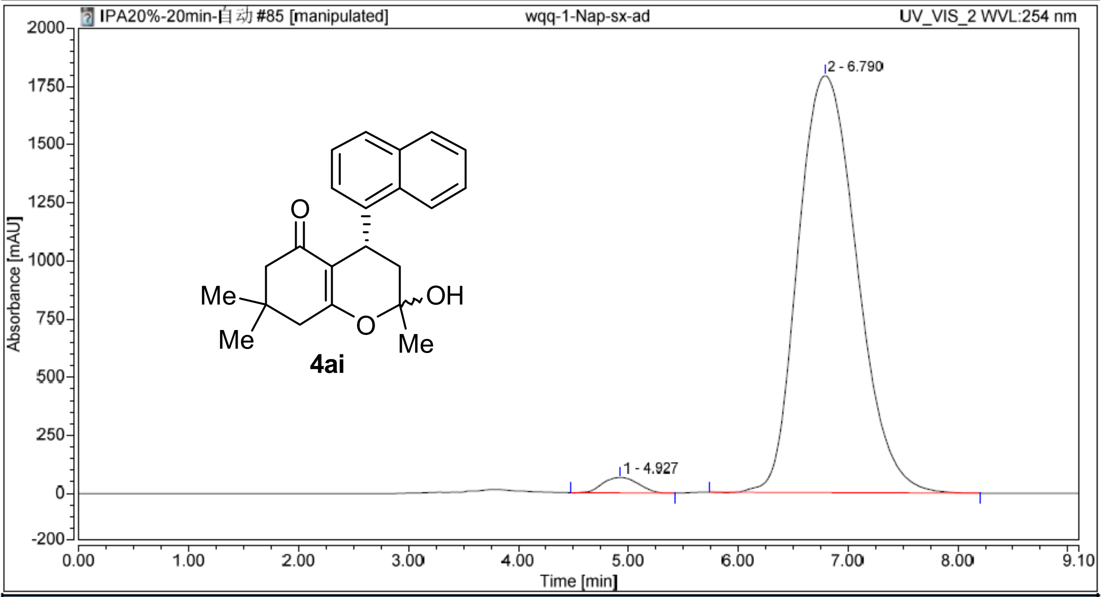

Integration Results

No.	Peak Name	Retention Time min	Area mAU*min	Height mAU	Relative Area %	Relative Height %	Amount n.a.
1		4.927	24.919	66.147	2.19	3.56	n.a.
2		6.790	1113.210	1792.027	97.81	96.44	n.a.
**Total:**			**1138.129**	**1858.175**	**100.00**	**100.00**	

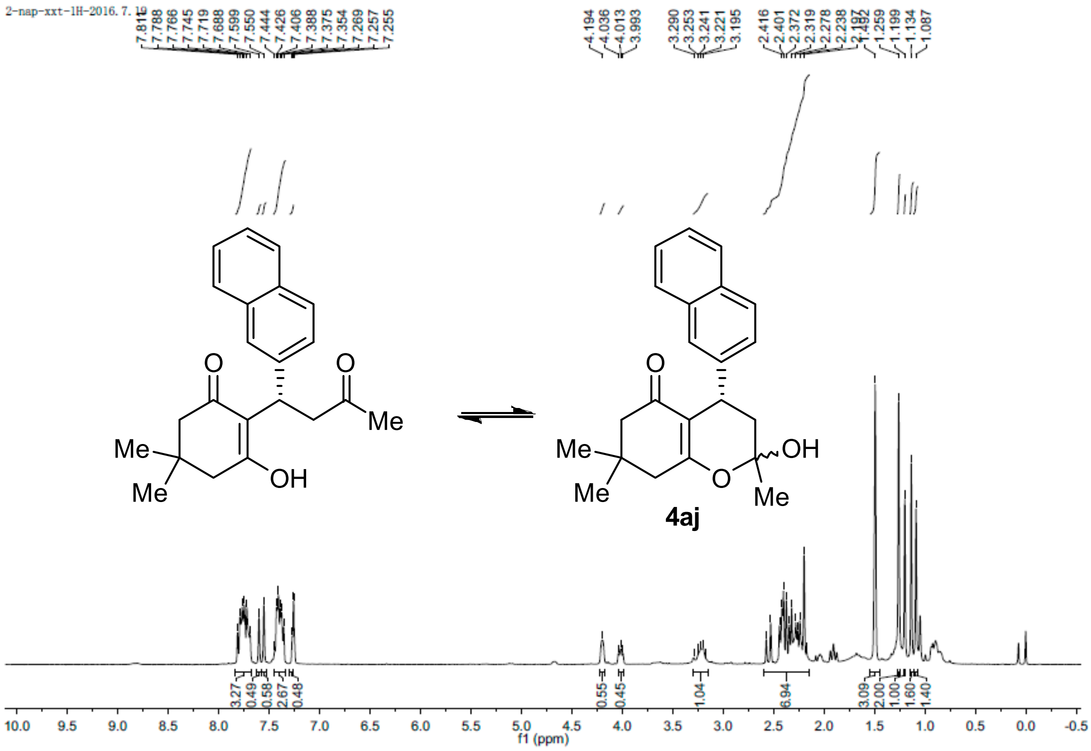
2-nap-xxt-1H-2016.7.1
Me
OH
4aj
f1 (ppm)

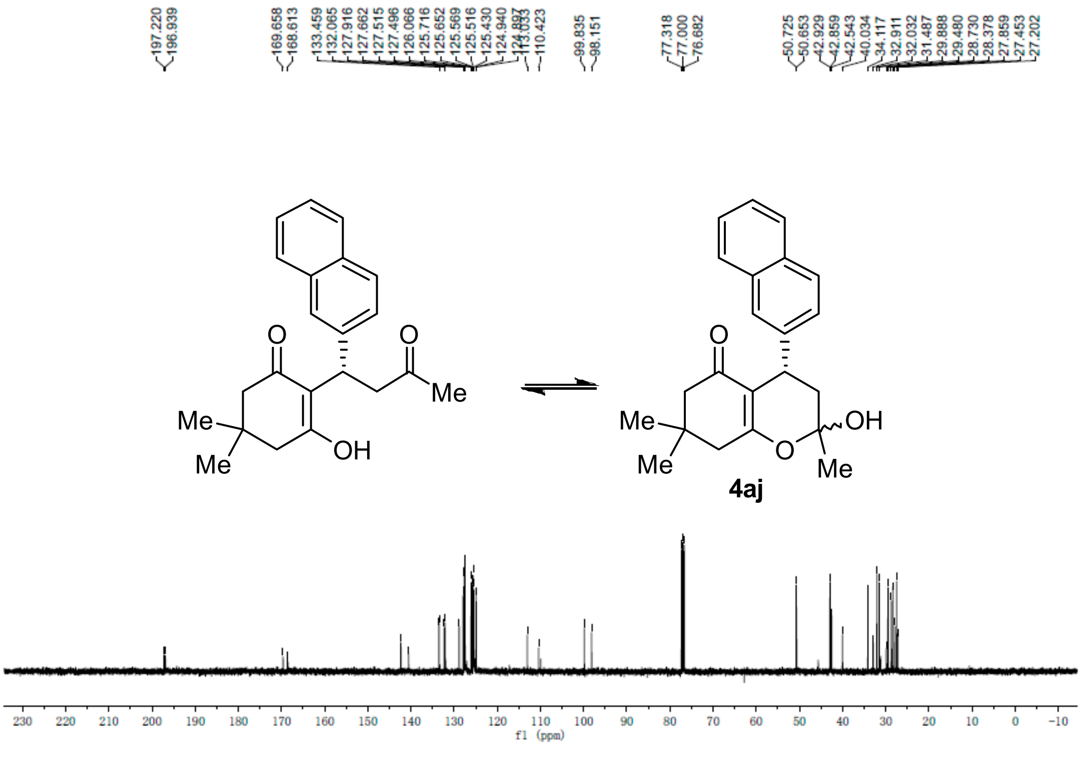
Me
OH
4aj
f1 (ppm)

Chromatogram

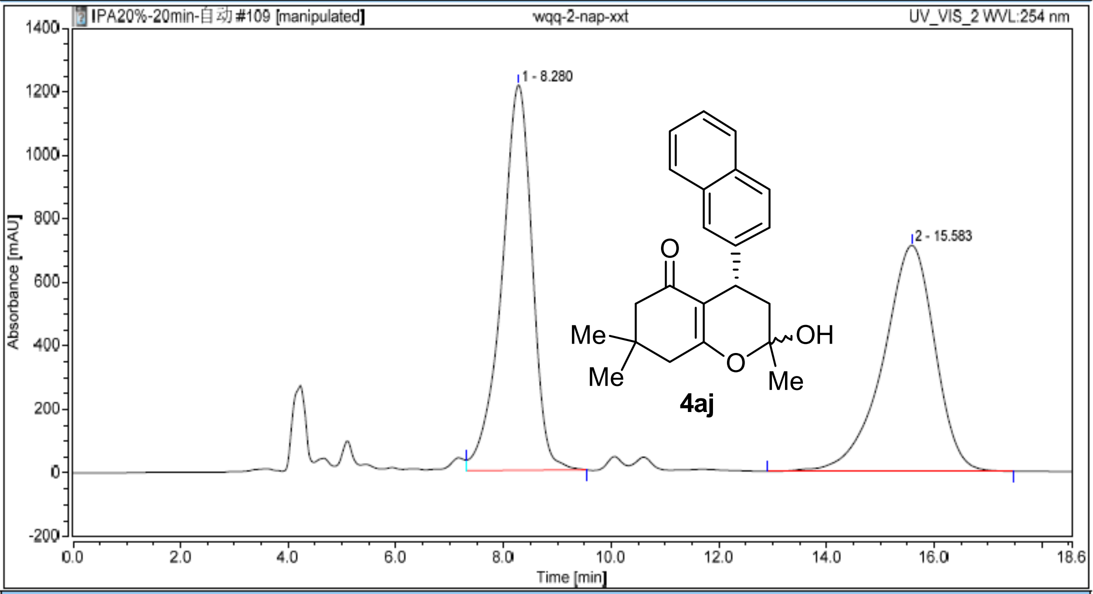

Integration Results

No.	Peak Name	Retention Time min	Area mAU*min	Height mAU	Relative Area %	Relative Height %	Amount n.a.
1		8.280	793.647	1213.141	50.01	63.05	n.a.
2		15.583	793.225	711.094	49.99	36.95	n.a.
Total:			1586.872	1924.234	100.00	100.00	

Chromatogram

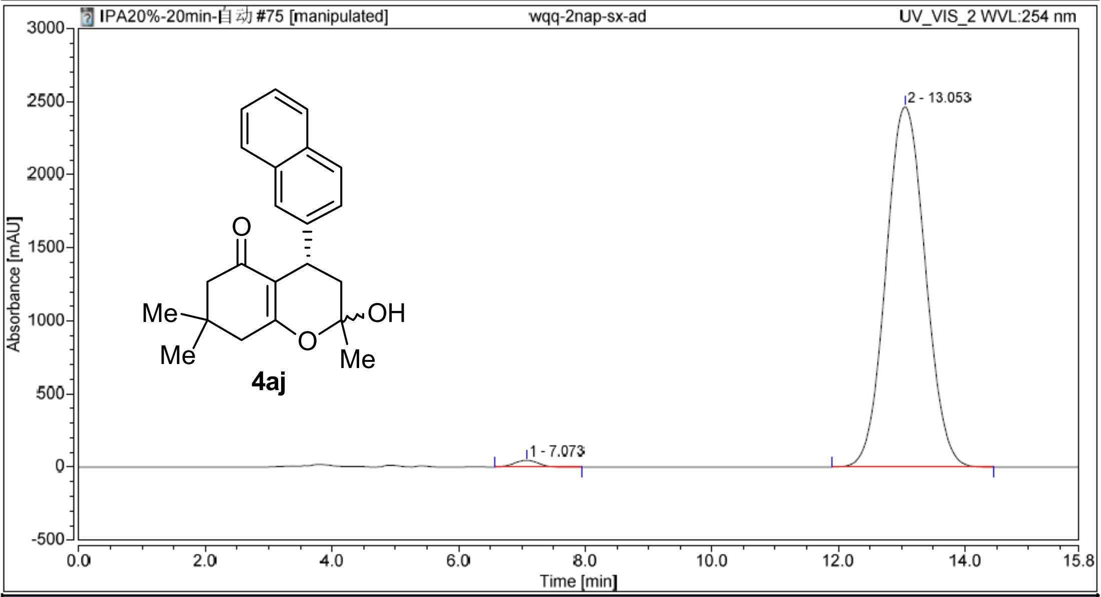

Integration Results

No.	Peak Name	Retention Time min	Area mAU*min	Height mAU	Relative Area %	Relative Height %	Amount n.a.
1		7.073	19.864	45.203	1.11	1.80	n.a.
2		13.053	1777.168	2464.231	98.89	98.20	n.a.
Total:			1797.031	2509.435	100.00	100.00	

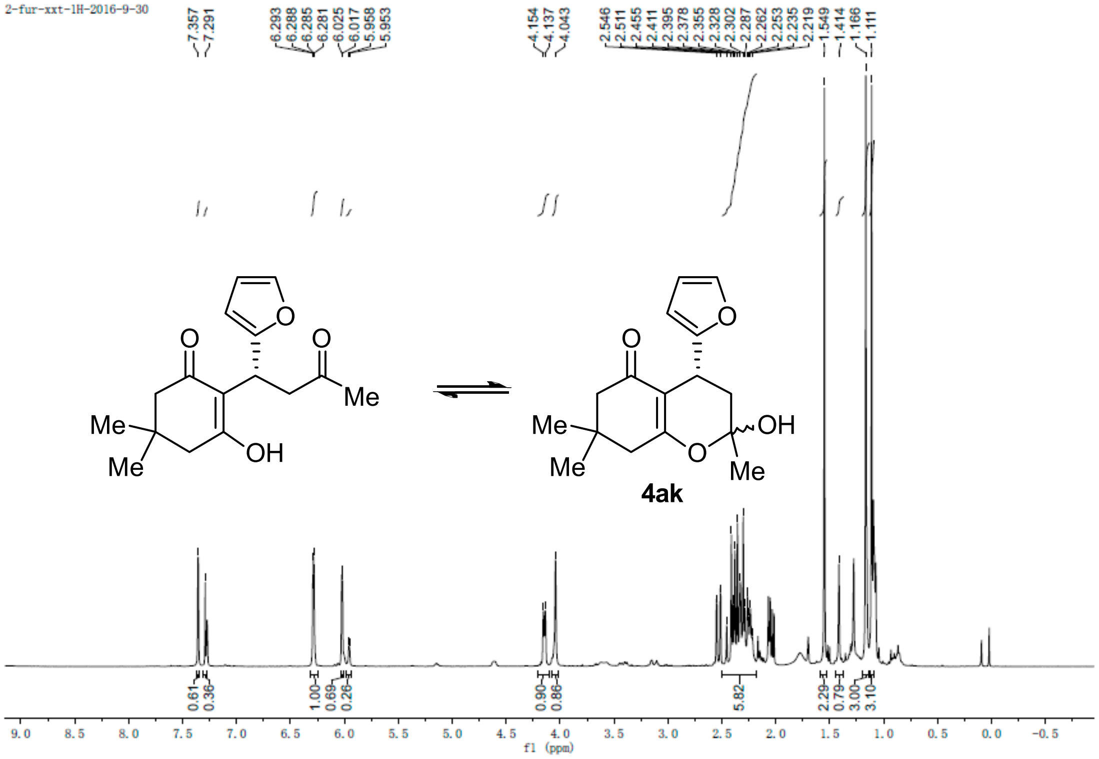
2-fur-xxt-1H-2016-9-30
7.357
7.291
6.293
6.288
6.285
6.281
6.025
6.017
5.958
5.953
4.154
4.137
4.043
2.546
2.511
2.455
2.411
2.395
2.378
2.355
2.328
2.302
2.287
2.262
2.253
2.235
2.219
1.549
1.414
1.166
1.111
O
O
O
Me
Me
Me
OH
O
O
O
Me
Me
OH
Me
4ak
0.61
0.36
1.00
0.69
0.26
0.90
0.86
5.82
2.29
0.79
3.00
3.10
f1 (ppm)

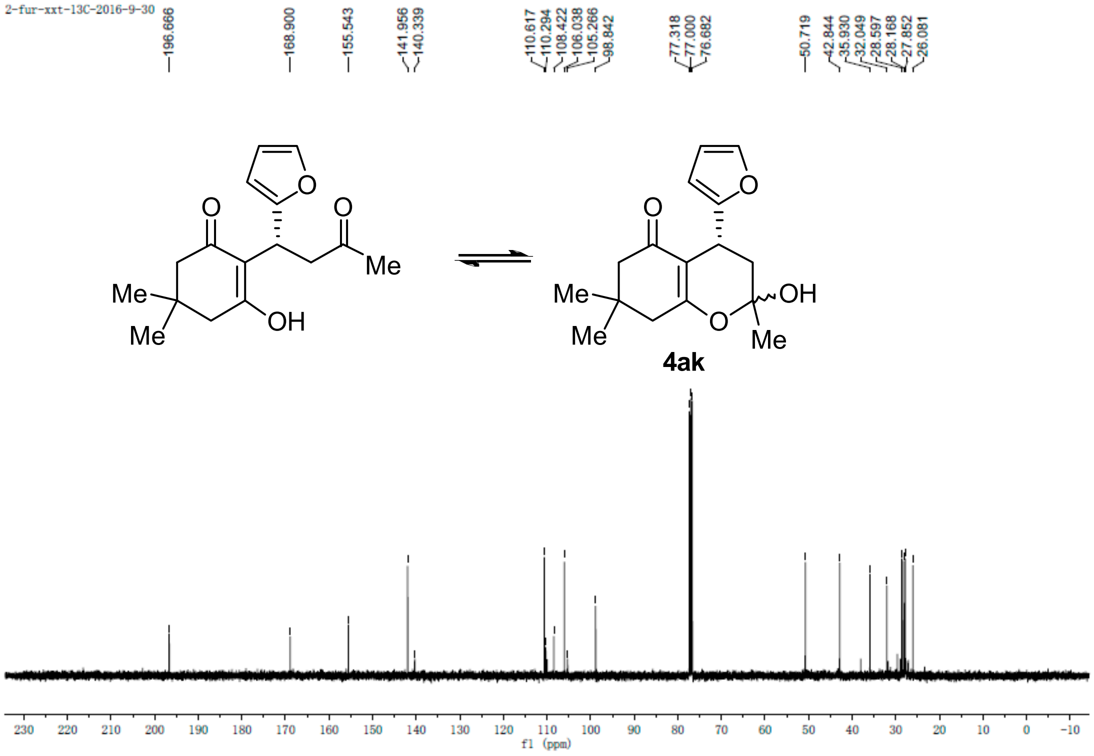
2-fur-xxt-13C-2016-9-30
196.666
168.900
155.543
141.956
140.339
110.617
110.294
108.422
106.038
105.266
98.842
77.318
77.000
76.682
50.719
42.844
35.930
32.049
28.597
28.168
27.852
26.081
O
O
O
Me
Me
Me
OH
O
O
O
Me
Me
OH
Me
4ak
f1 (ppm)

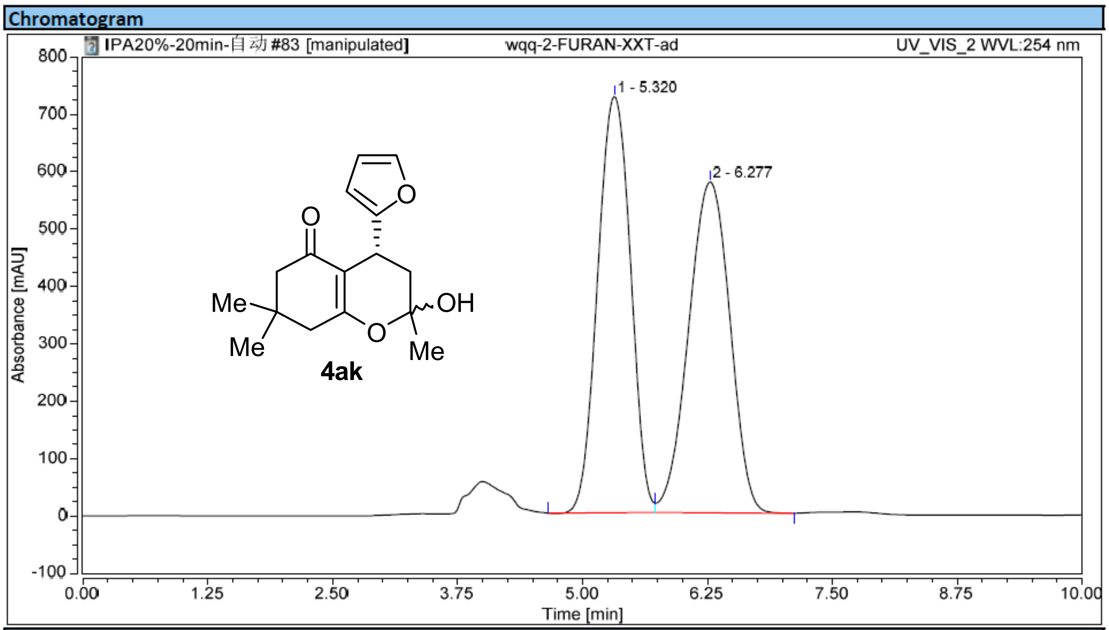

Integration Results							
No.	Peak Name	Retention Time<br>min	Area<br>mAU*min	Height<br>mAU	Relative Area<br>%	Relative Height<br>%	Amount<br>n.a.
1		5.320	276.320	725.621	50.01	55.71	n.a.
2		6.277	276.219	576.859	49.99	44.29	n.a.
**Total:**			**552.539**	**1302.480**	**100.00**	**100.00**	

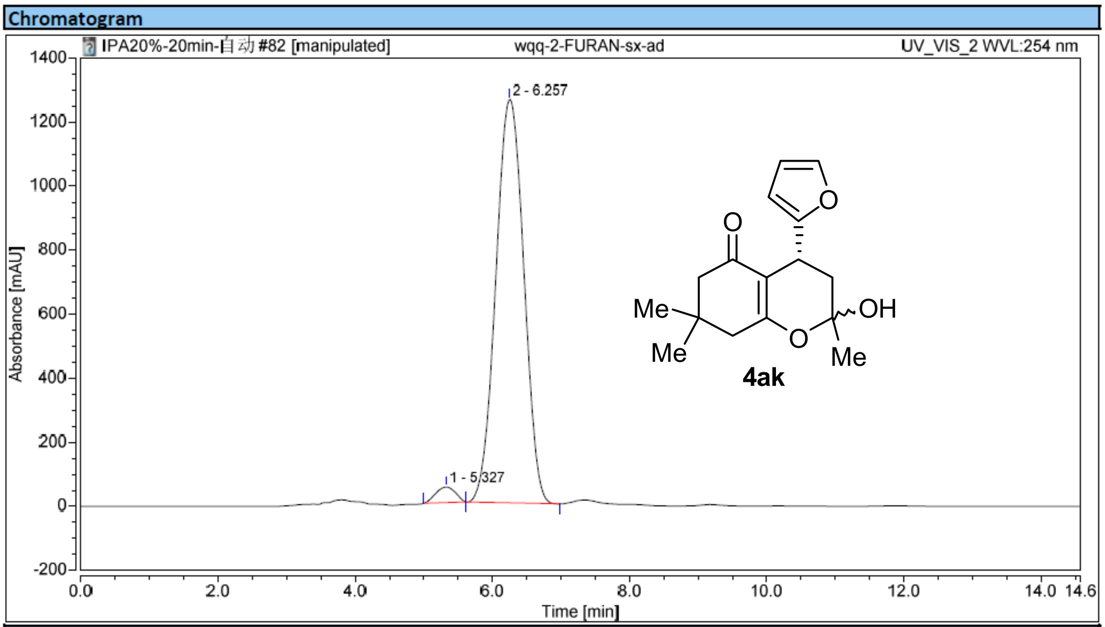

Integration Results							
No.	Peak Name	Retention Time<br>min	Area<br>mAU*min	Height<br>mAU	Relative Area<br>%	Relative Height<br>%	Amount<br>n.a.
1		5.327	16.196	48.459	2.65	3.70	n.a.
2		6.257	595.956	1259.864	97.35	96.30	n.a.
**Total:**			**612.152**	**1308.323**	**100.00**	**100.00**	

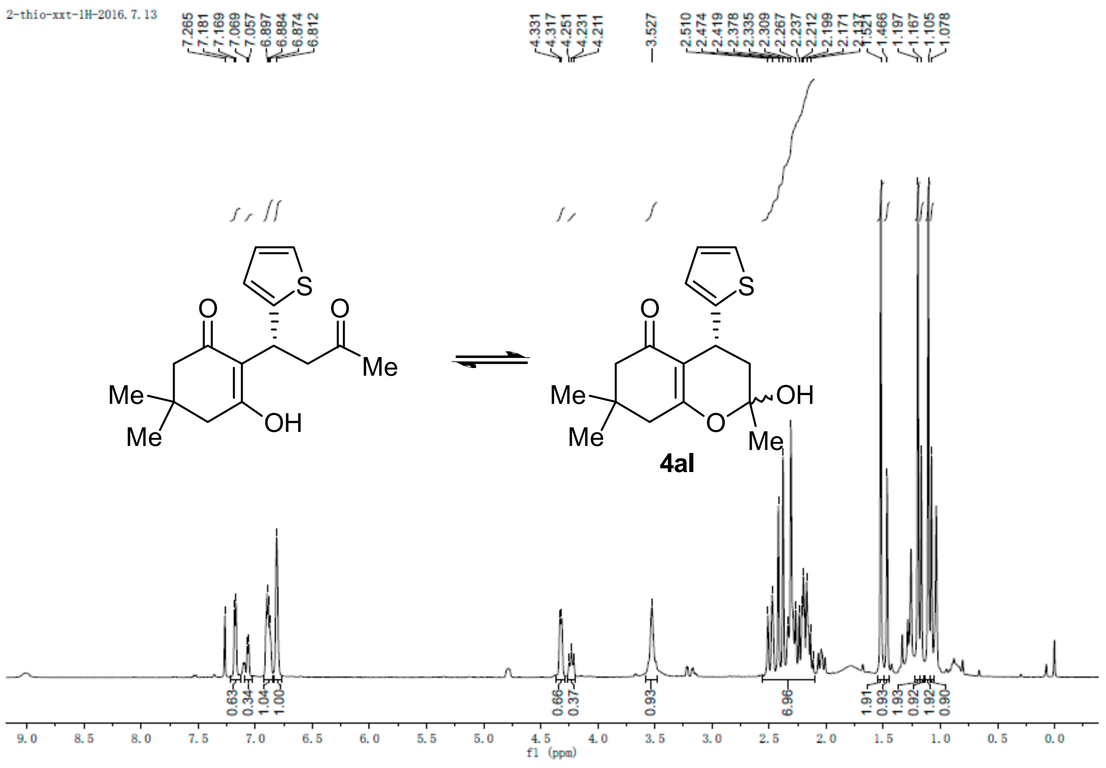
2-thio-xxt-1H-2016.7.13
4al
f1 (ppm)

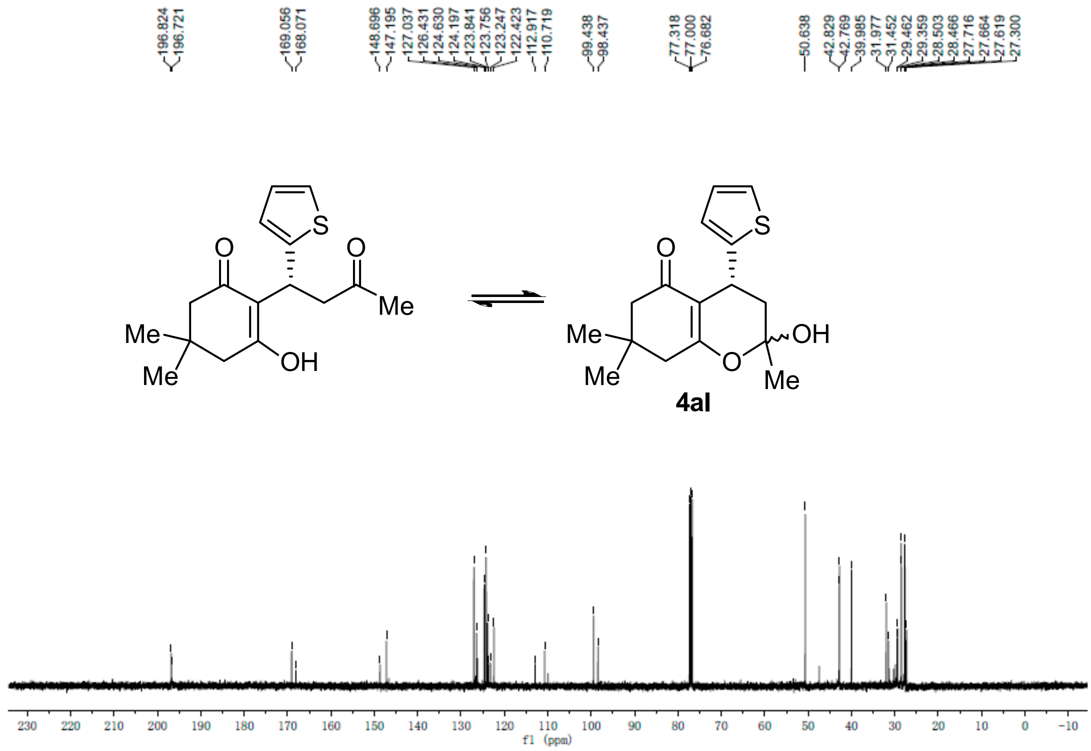
4al
f1 (ppm)

Chromatogram

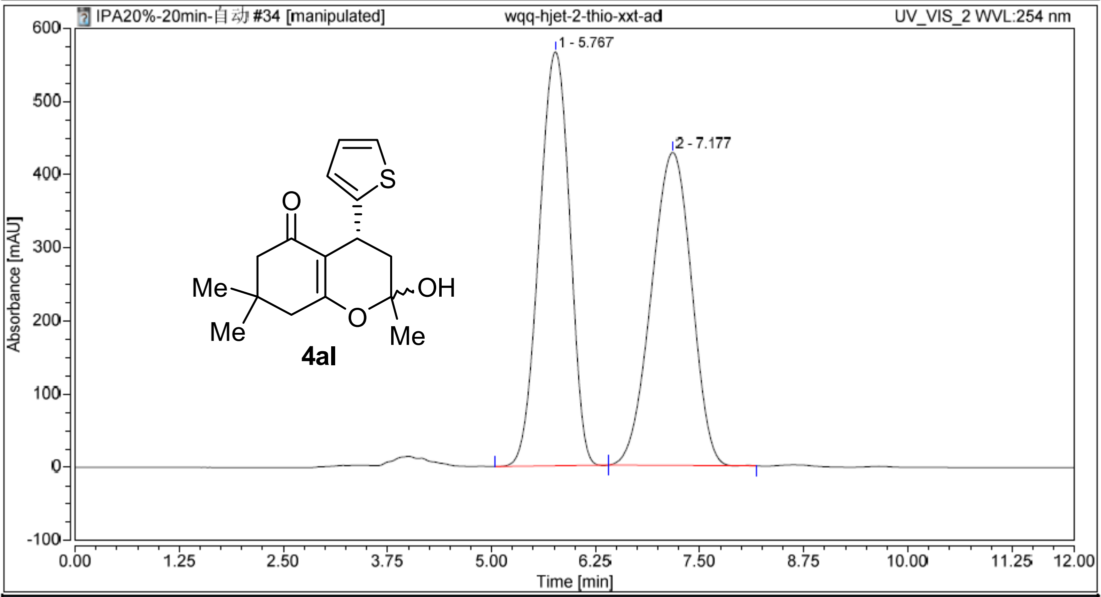

Integration Results

No.	Peak Name	Retention Time min	Area mAU*min	Height mAU	Relative Area %	Relative Height %	Amount n.a.
1		5.767	240.271	565.982	50.15	56.95	n.a.
2		7.177	238.831	427.759	49.85	43.05	n.a.
**Total:**			**479.102**	**993.741**	**100.00**	**100.00**	

Chromatogram

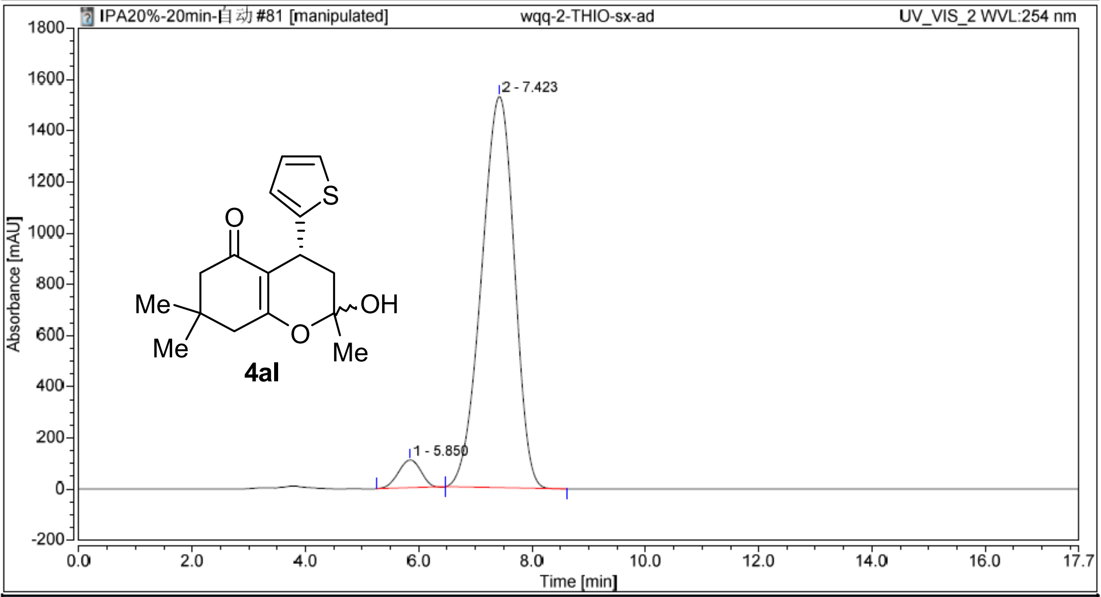

Integration Results

No.	Peak Name	Retention Time min	Area mAU*min	Height mAU	Relative Area %	Relative Height %	Amount n.a.
1		5.850	49.415	108.436	4.61	6.63	n.a.
2		7.423	1021.583	1527.918	95.39	93.37	n.a.
**Total:**			**1070.998**	**1636.354**	**100.00**	**100.00**	

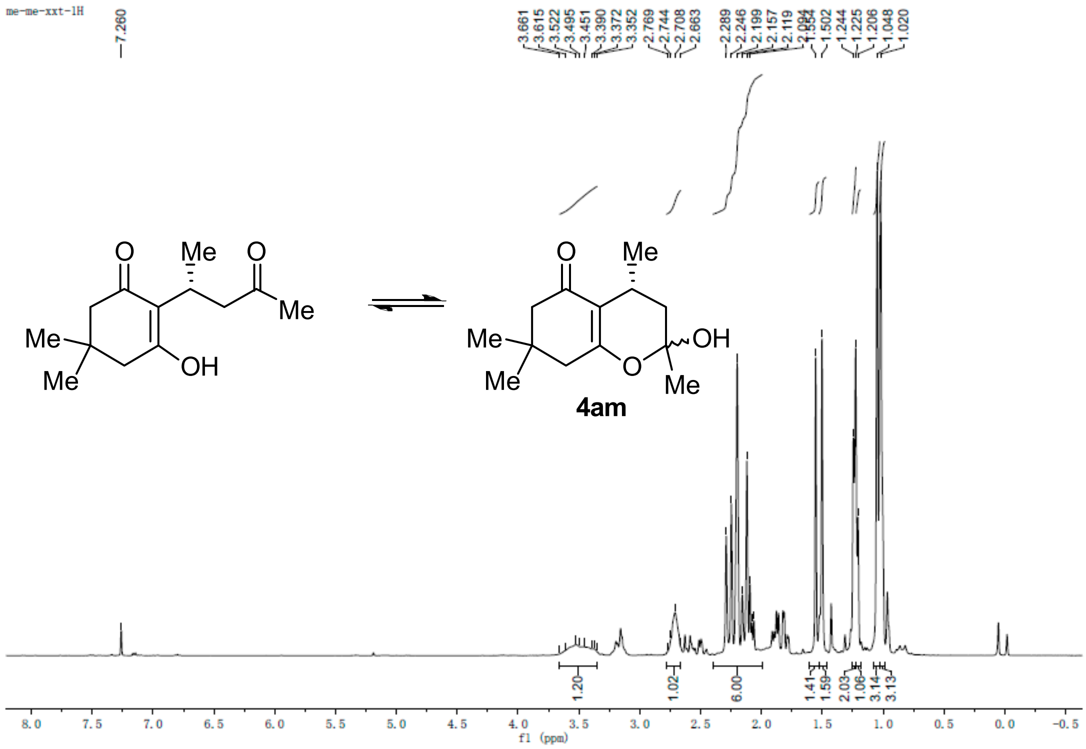

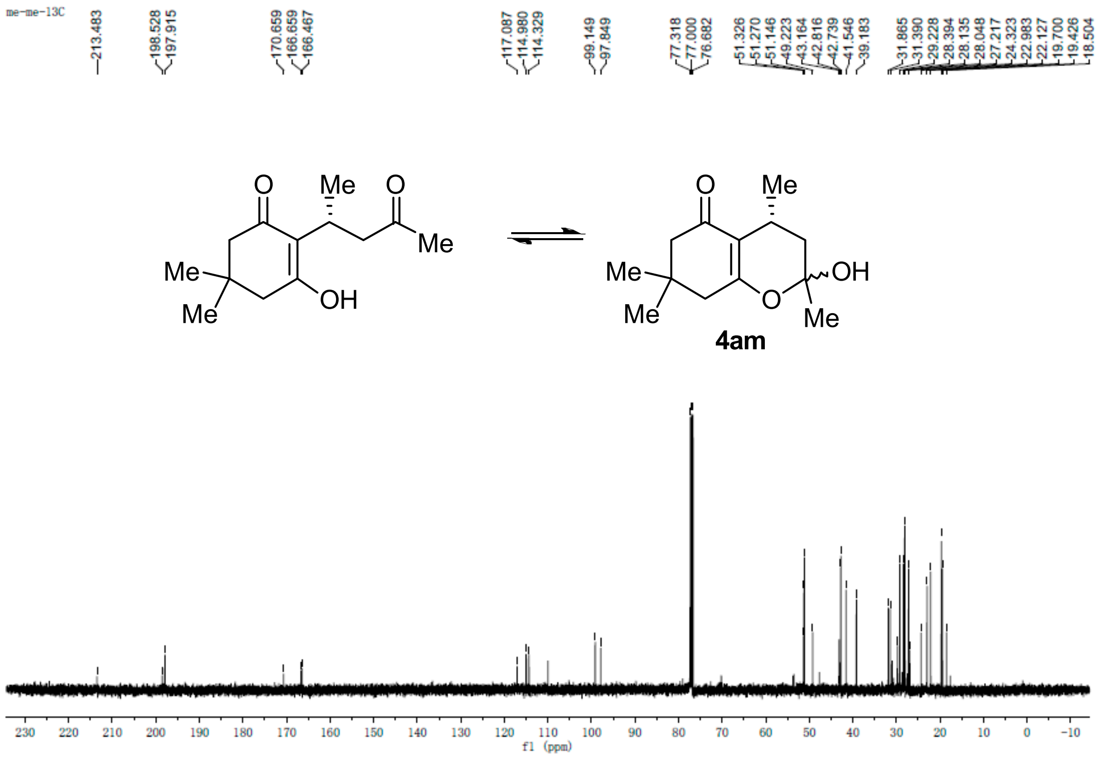

Chromatogram

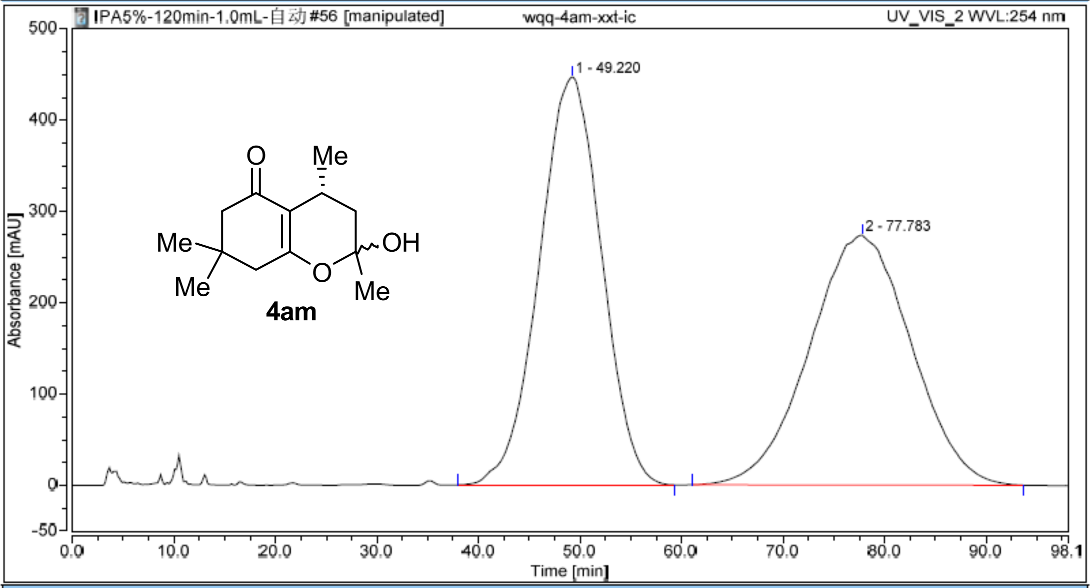

Integration Results

No.	Peak Name	Retention Time min	Area mAU*min	Height mAU	Relative Area %	Relative Height %	Amount n.a.
1		49.220	3182.503	446.892	50.05	62.01	n.a.
2		77.783	3175.854	273.750	49.95	37.99	n.a.
**Total:**			**6358.356**	**720.643**	**100.00**	**100.00**	

Chromatogram

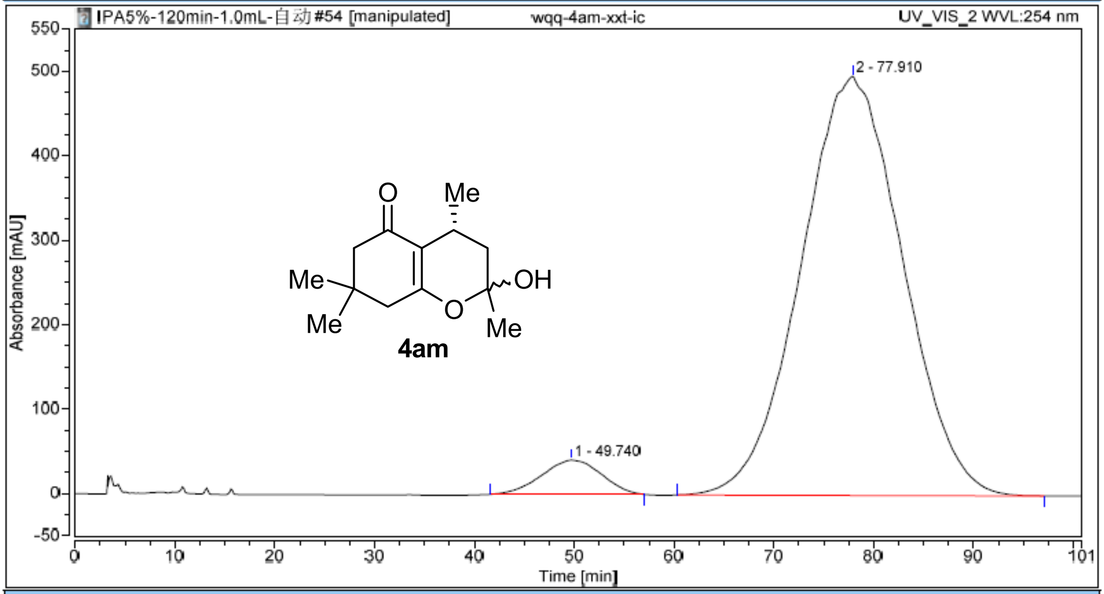

Integration Results

No.	Peak Name	Retention Time min	Area mAU*min	Height mAU	Relative Area %	Relative Height %	Amount n.a.
1		49.740	280.689	40.760	4.57	7.59	n.a.
2		77.910	5857.410	496.318	95.43	92.41	n.a.
**Total:**			**6138.100**	**537.078**	**100.00**	**100.00**	

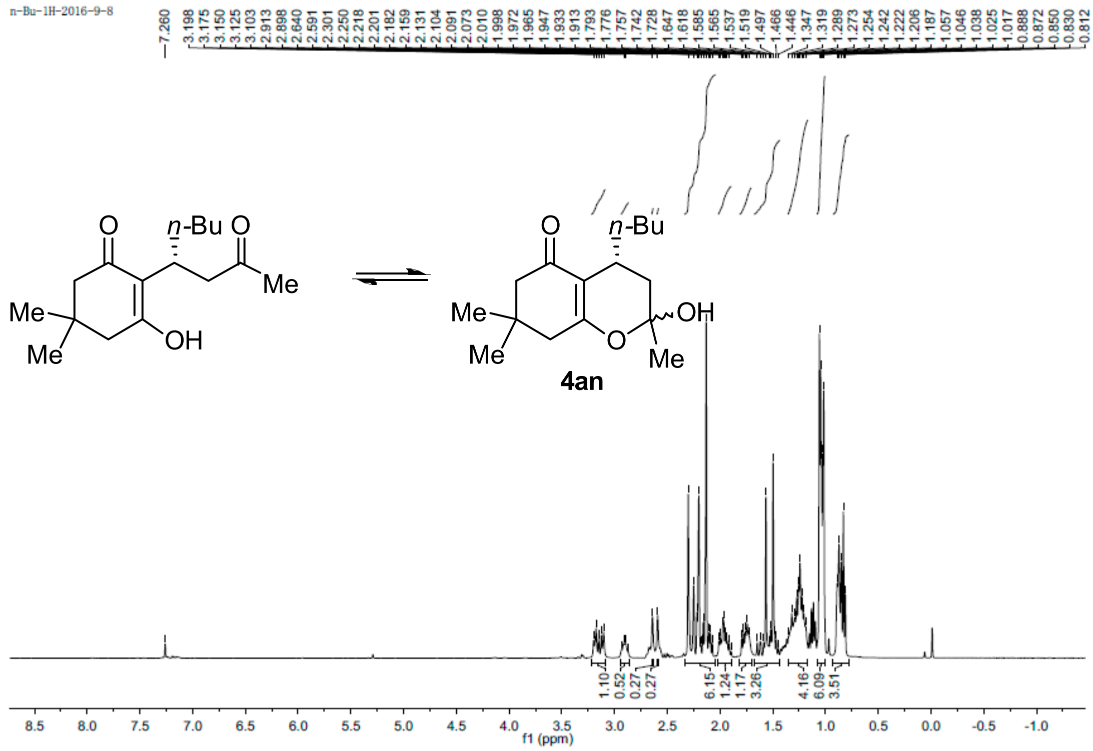
n-Bu-1H-2016-9-8
n-Bu
O
Me
OH
4an
f1 (ppm)

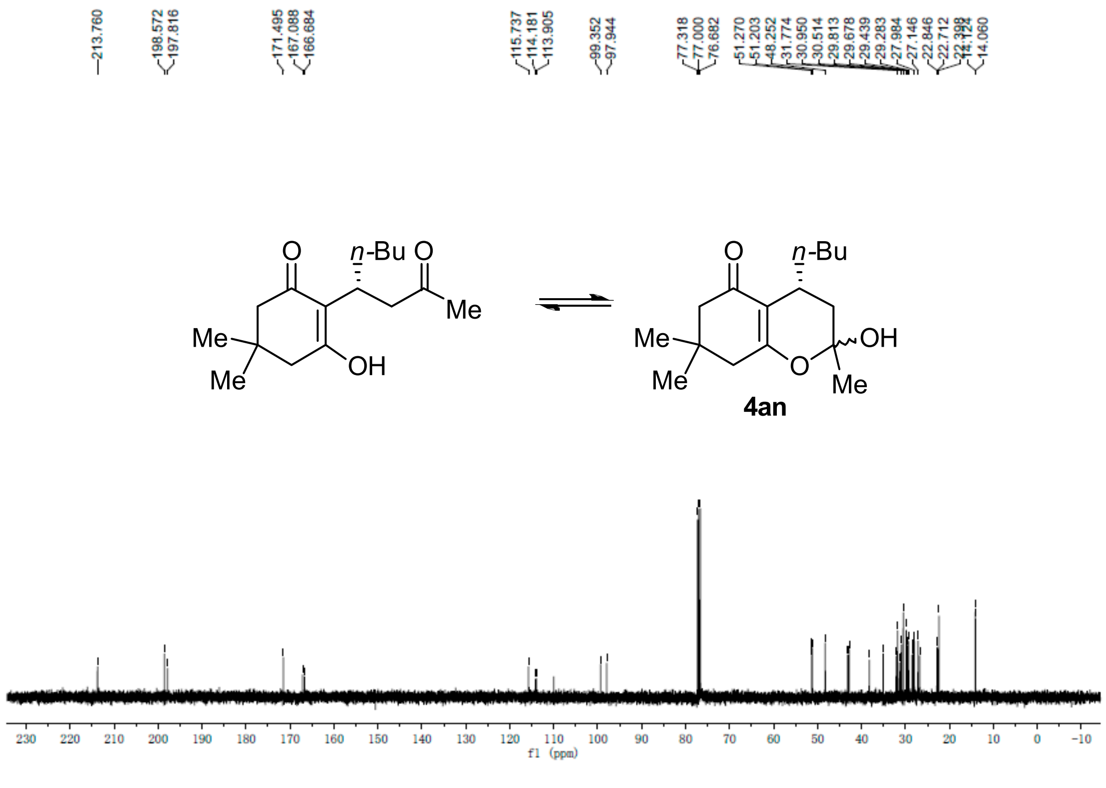
n-Bu
O
Me
OH
4an
f1 (ppm)

Chromatogram

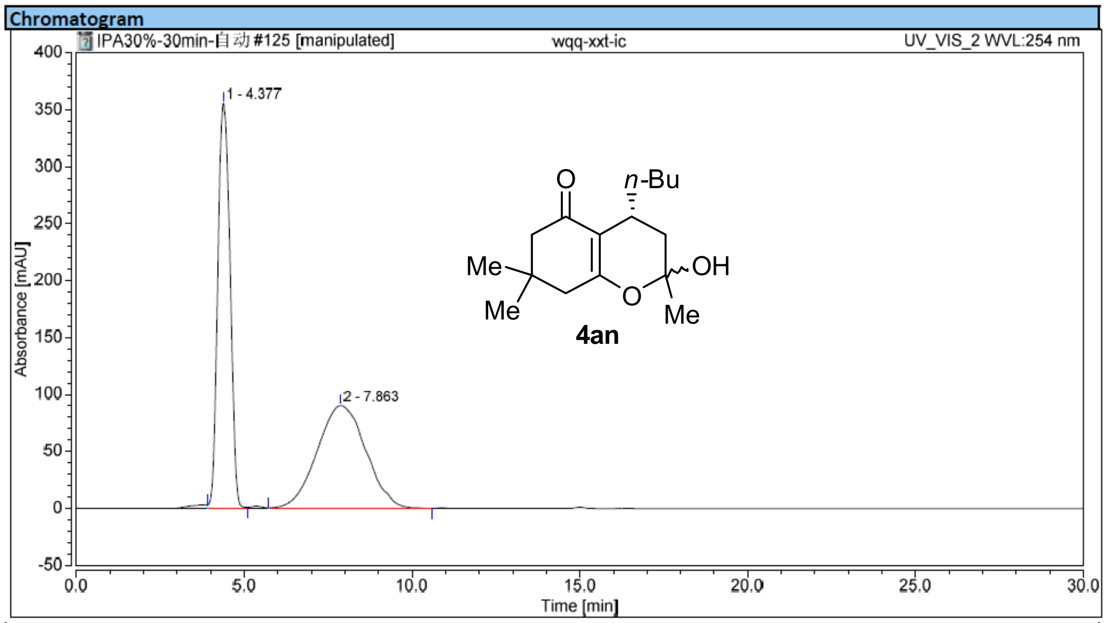

Integration Results

No.	Peak Name	Retention Time min	Area mAU*min	Height mAU	Relative Area %	Relative Height %	Amount n.a.
1		4.377	150.702	356.188	49.75	79.77	n.a.
2		7.863	152.209	90.305	50.25	20.23	n.a.
Total:			302.910	446.493	100.00	100.00	

Chromatogram

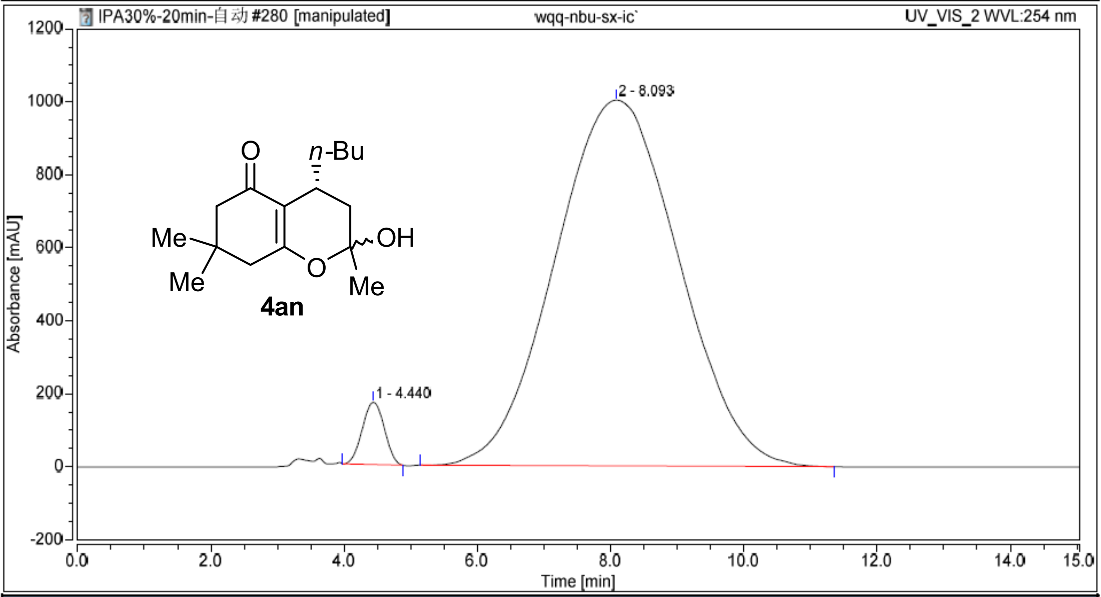

Integration Results

No.	Peak Name	Retention Time min	Area mAU*min	Height mAU	Relative Area %	Relative Height %	Amount n.a.
1		4.440	65.412	170.567	2.94	14.54	n.a.
2		8.093	2155.779	1002.202	97.06	85.46	n.a.
Total:			2221.191	1172.769	100.00	100.00	

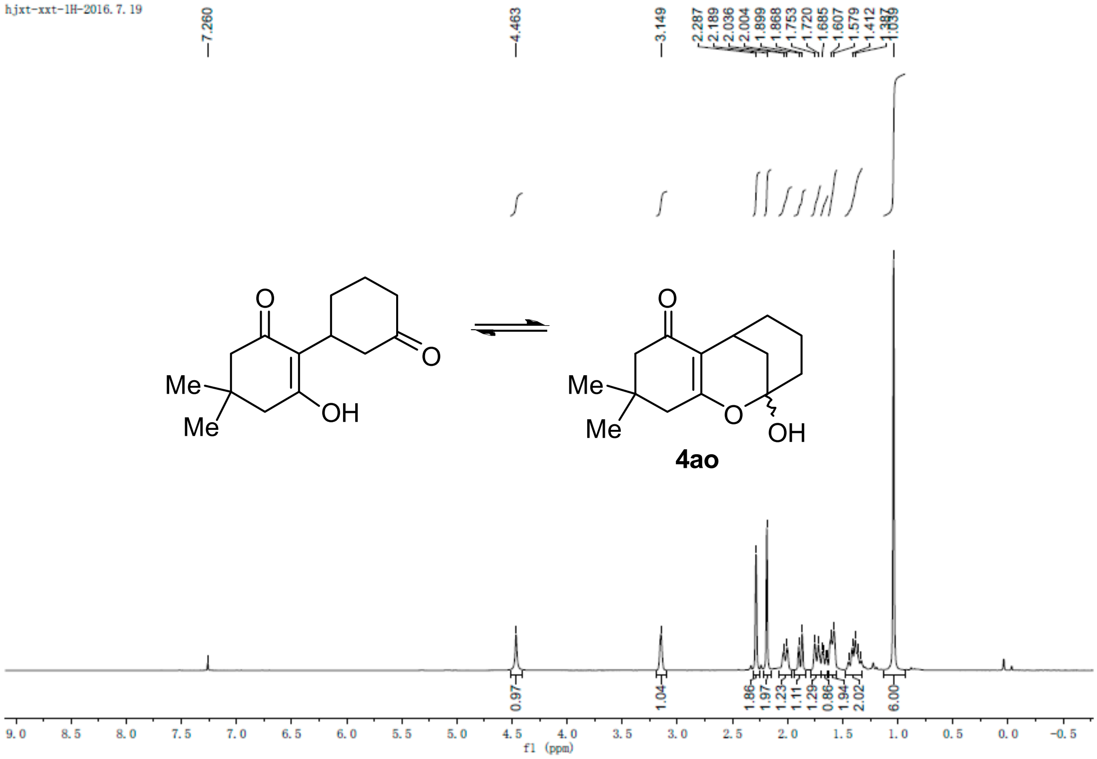
hjxt-xxt-1H-2016. 7. 19
7.260
4.463
3.149
2.287
2.189
2.036
2.004
1.899
1.868
1.753
1.720
1.685
1.607
1.579
1.412
Me
Me
OH
O
4ao
0.97
1.04
6.00
f1 (ppm)

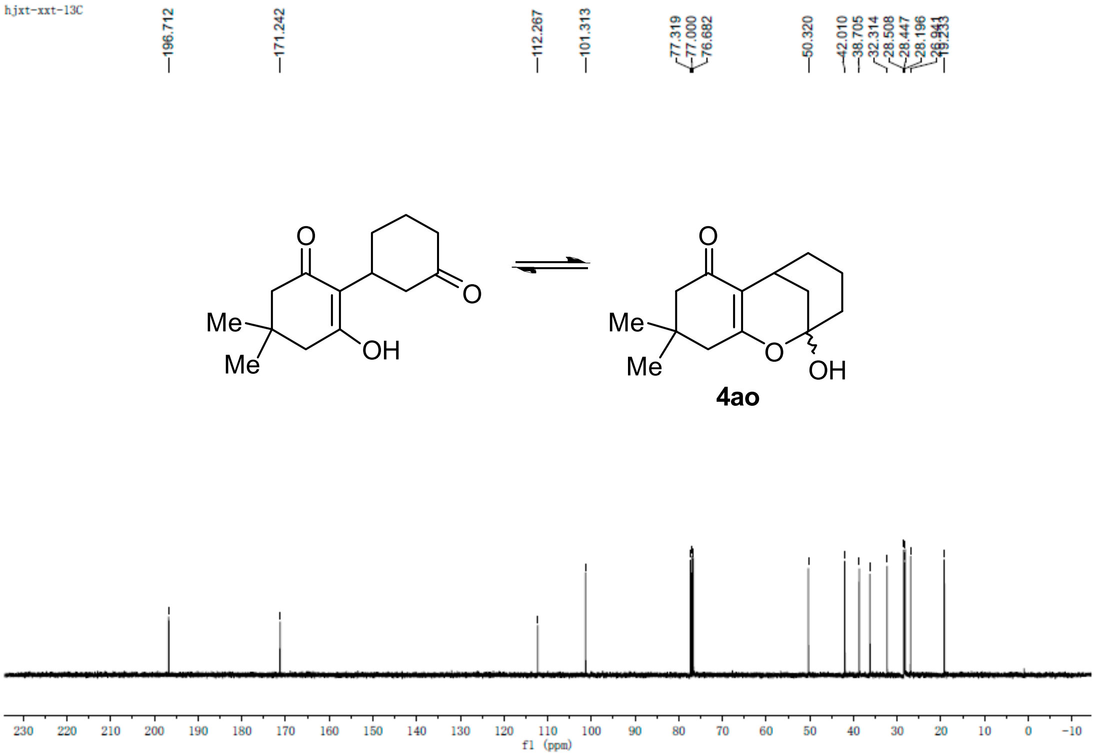
hjxt-xxt-13C
196.712
171.242
112.267
101.313
77.319
77.000
76.682
50.320
42.010
38.705
32.314
28.508
28.447
28.196
Me
Me
OH
O
4ao
f1 (ppm)

Chromatogram

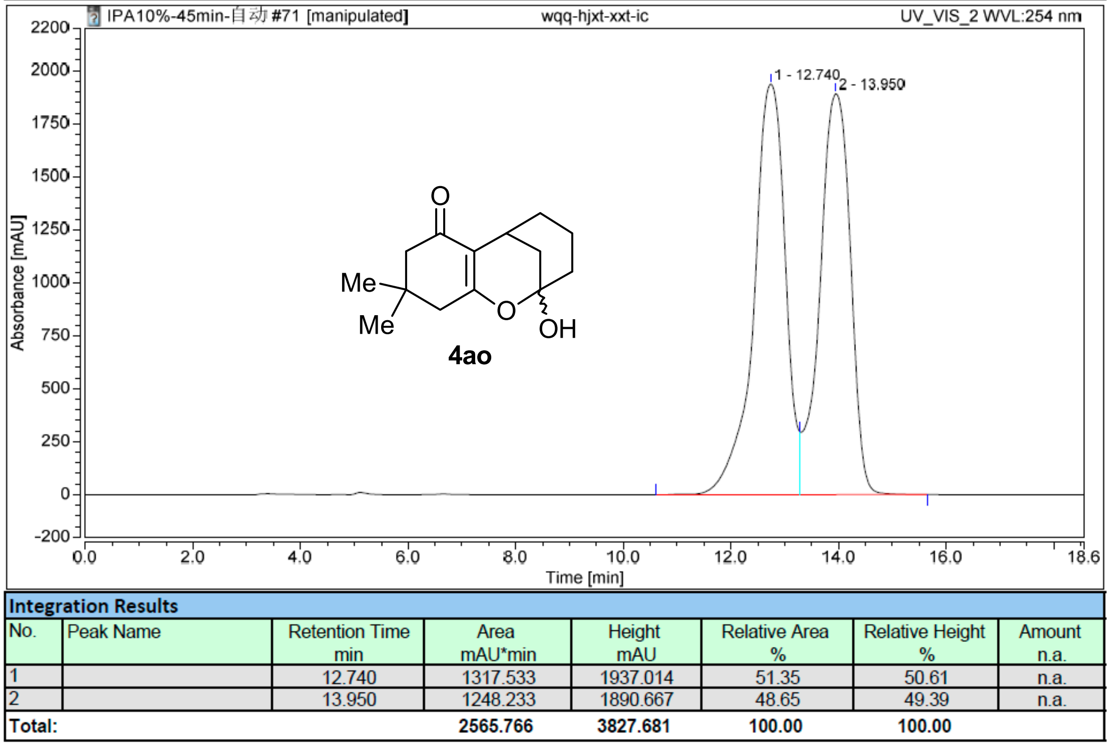

Integration Results

No.	Peak Name	Retention Time min	Area mAU*min	Height mAU	Relative Area %	Relative Height %	Amount n.a.
1		12.740	1317.533	1937.014	51.35	50.61	n.a.
2		13.950	1248.233	1890.667	48.65	49.39	n.a.
**Total:**			**2565.766**	**3827.681**	**100.00**	**100.00**	

Chromatogram

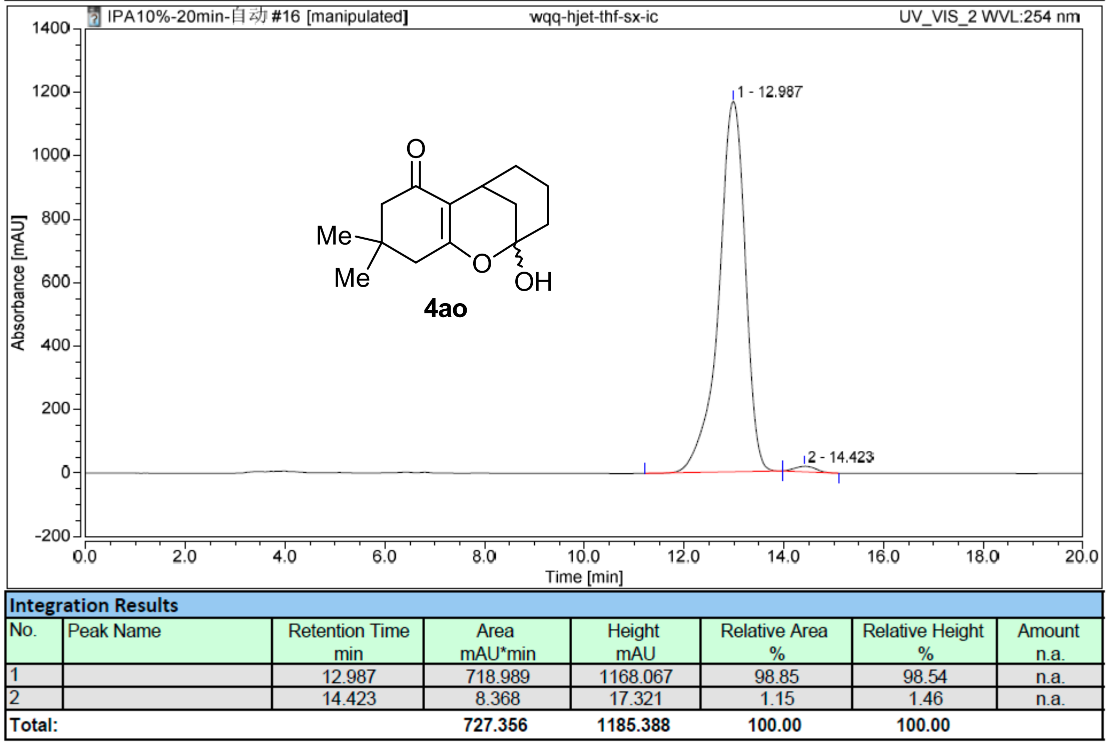

Integration Results

No.	Peak Name	Retention Time min	Area mAU*min	Height mAU	Relative Area %	Relative Height %	Amount n.a.
1		12.987	718.989	1168.067	98.85	98.54	n.a.
2		14.423	8.368	17.321	1.15	1.46	n.a.
**Total:**			**727.356**	**1185.388**	**100.00**	**100.00**	

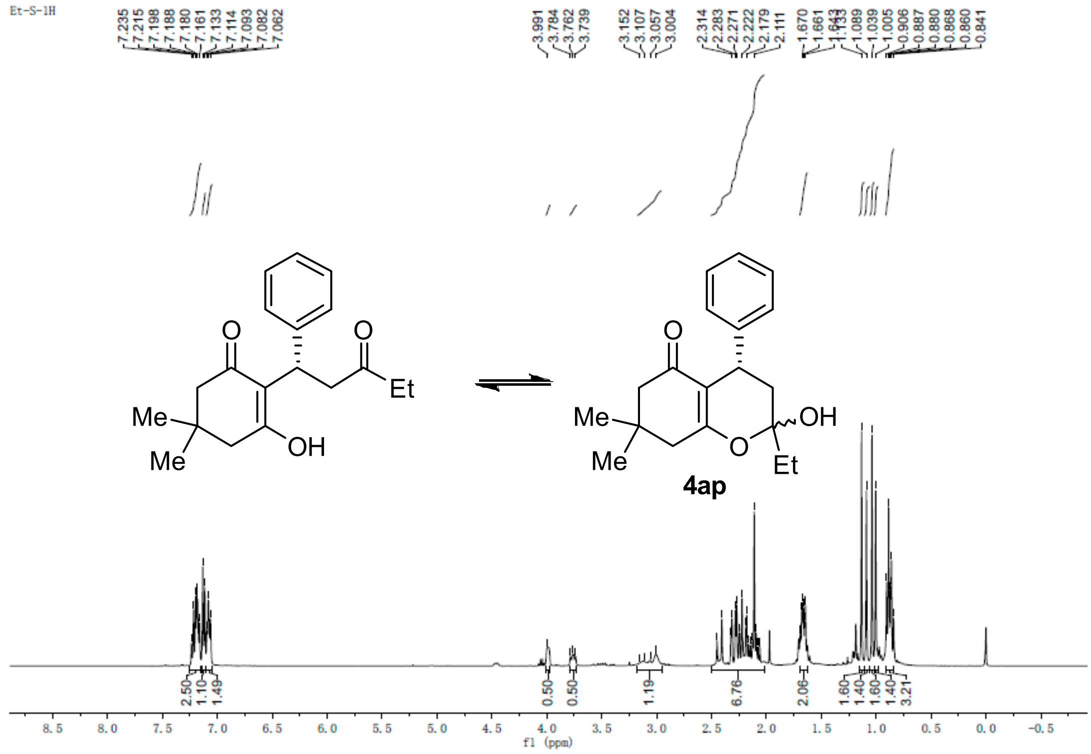
Et-S-1H
Me
Me
OH
O
O
Et
OH
O
Et
4ap
f1 (ppm)

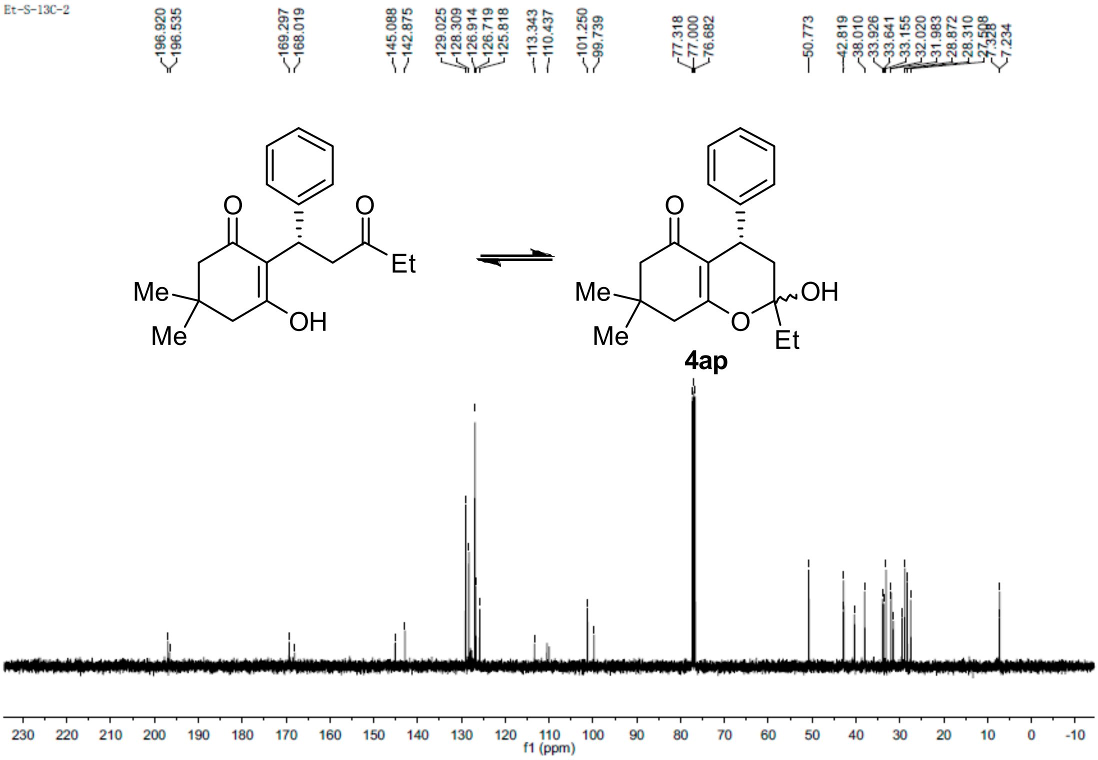
Et-S-13C-2
Me
Me
OH
O
O
Et
OH
O
Et
4ap
f1 (ppm)

Chromatogram

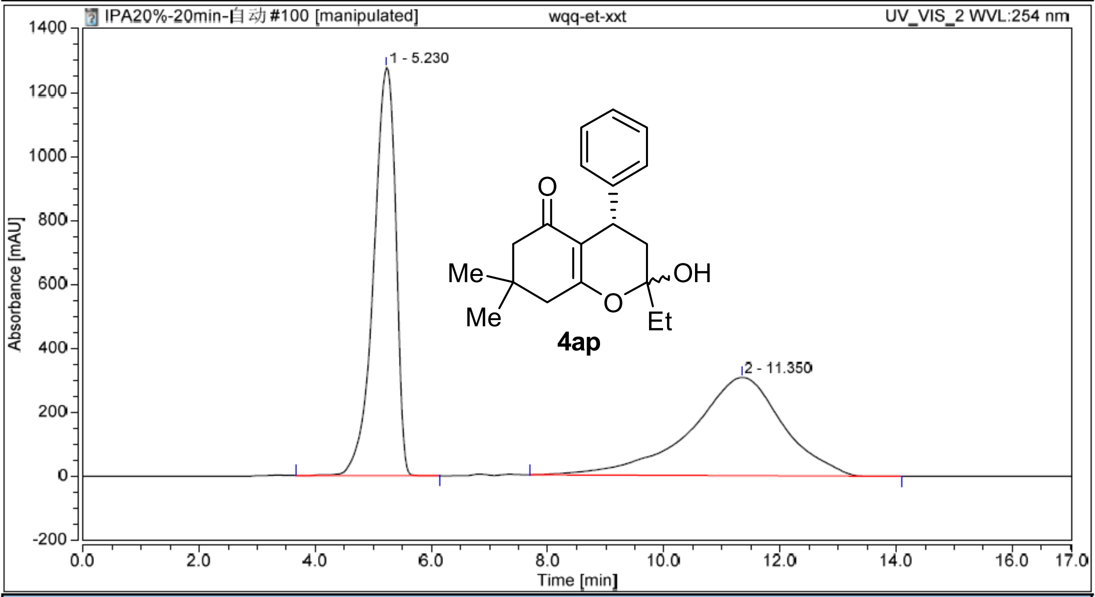

Integration Results

No.	Peak Name	Retention Time min	Area mAU*min	Height mAU	Relative Area %	Relative Height %	Amount n.a.
1		5.230	564.020	1276.641	50.02	80.61	n.a.
2		11.350	563.678	307.137	49.98	19.39	n.a.
**Total:**			**1127.698**	**1583.777**	**100.00**	**100.00**	

Chromatogram

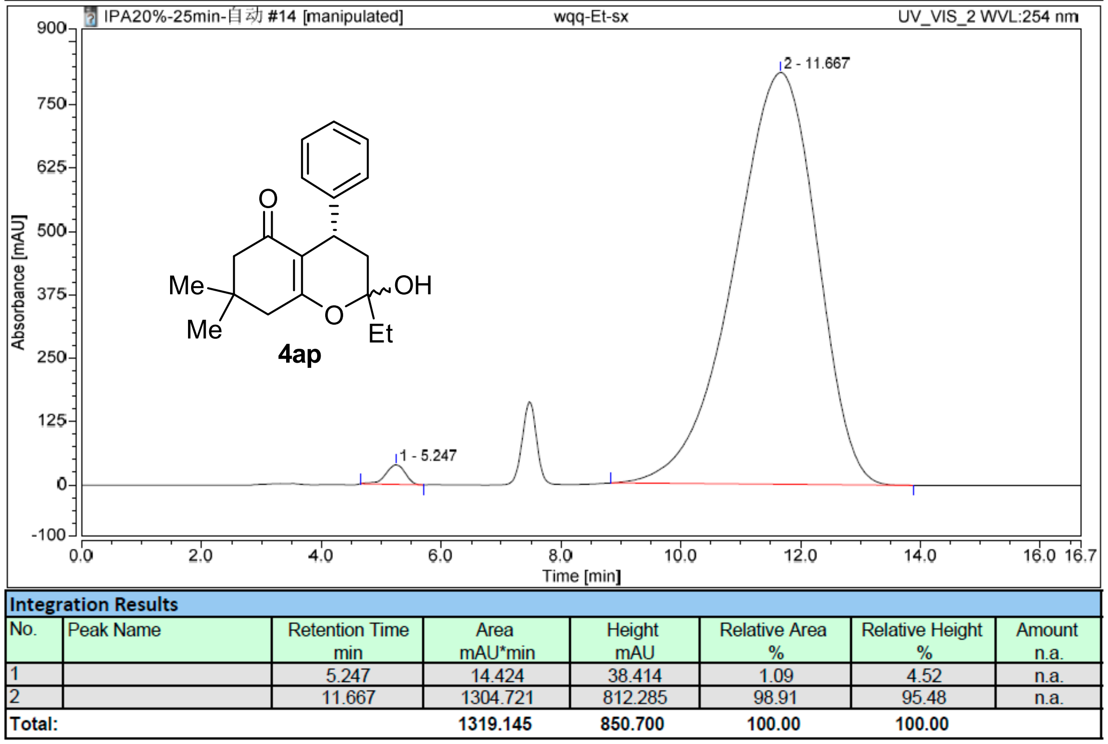

Integration Results

No.	Peak Name	Retention Time min	Area mAU*min	Height mAU	Relative Area %	Relative Height %	Amount n.a.
1		5.247	14.424	38.414	1.09	4.52	n.a.
2		11.667	1304.721	812.285	98.91	95.48	n.a.
**Total:**			**1319.145**	**850.700**	**100.00**	**100.00**	

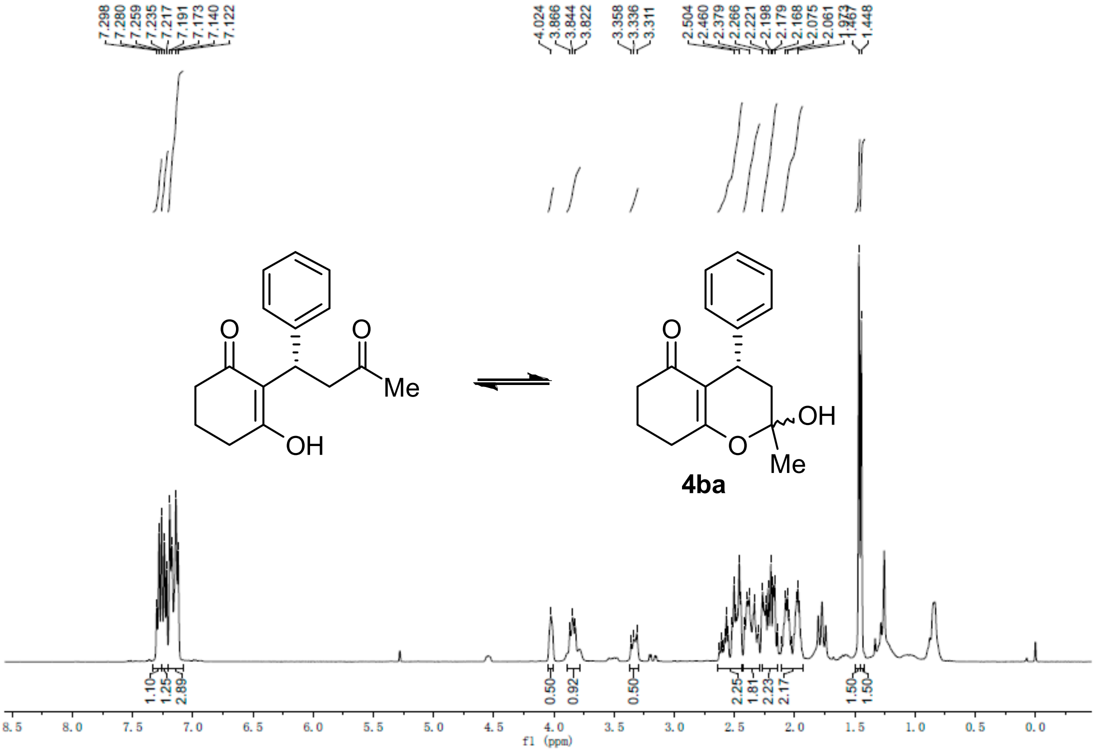
7.298 7.280 7.259 7.235 7.217 7.191 7.173 7.140 7.122
4.024 3.866 3.844 3.822
3.358 3.336 3.311
2.504 2.460 2.379 2.266 2.221 2.198 2.179 2.168 2.075 2.061 1.973 1.467 1.448
O
O
Me
OH
O
OH
O
Me
4ba
1.10 1.25 2.89
0.50 0.92
0.50
2.25 1.81 2.23 2.17
1.50 1.50
f1 (ppm)

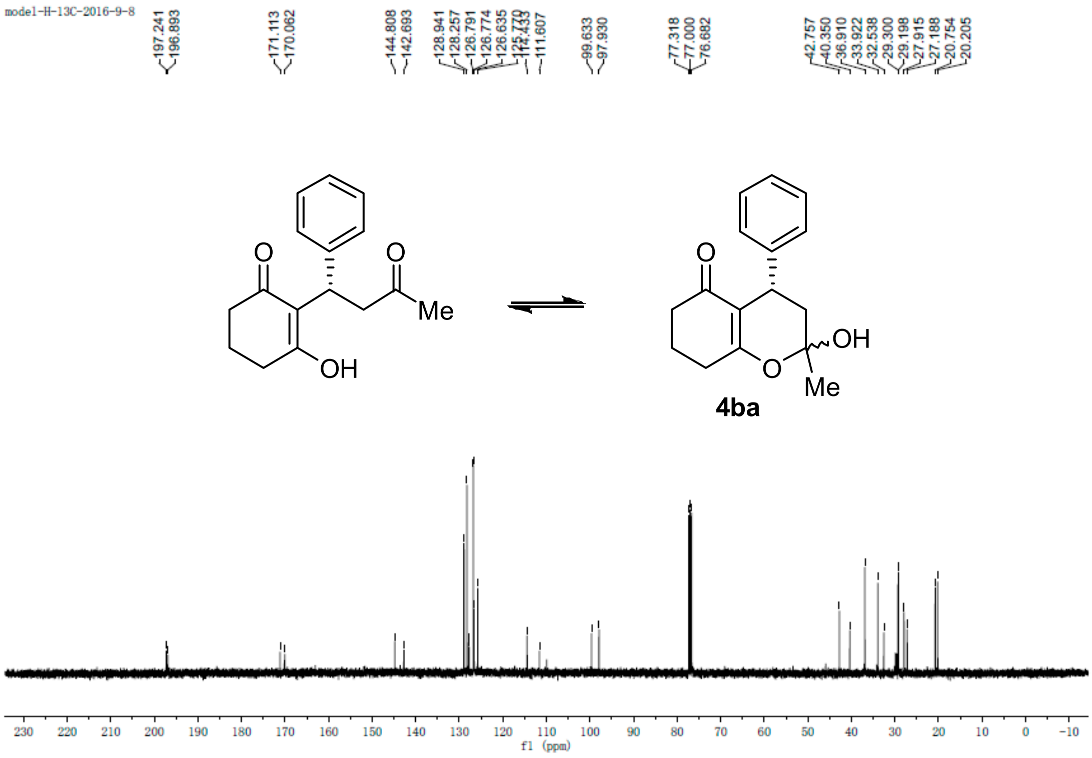
model-H-13C-2016-9-8
197.241 196.893
171.113 170.062
144.808 142.693
128.941 128.257 126.791 126.774 126.635 125.770
111.607
99.633 97.930
77.318 77.000 76.682
42.757 40.350 36.910 33.922 32.538 29.300 29.198 27.915 27.188 20.754 20.205
O
O
Me
OH
O
OH
O
Me
4ba
f1 (ppm)

Chromatogram

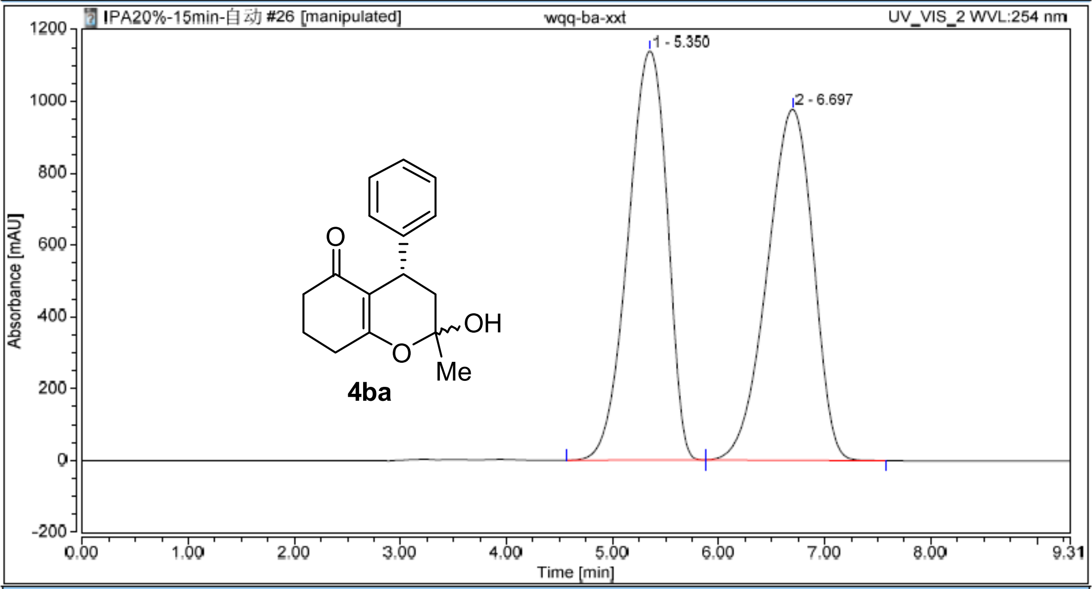

Integration Results

No.	Peak Name	Retention Time<br>min	Area<br>mAU*min	Height<br>mAU	Relative Area<br>%	Relative Height<br>%	Amount<br>n.a.
1		5.350	487.664	1138.403	49.81	53.82	n.a.
2		6.697	491.333	976.722	50.19	46.18	n.a.
Total:			**978.997**	**2115.125**	**100.00**	**100.00**	

Chromatogram

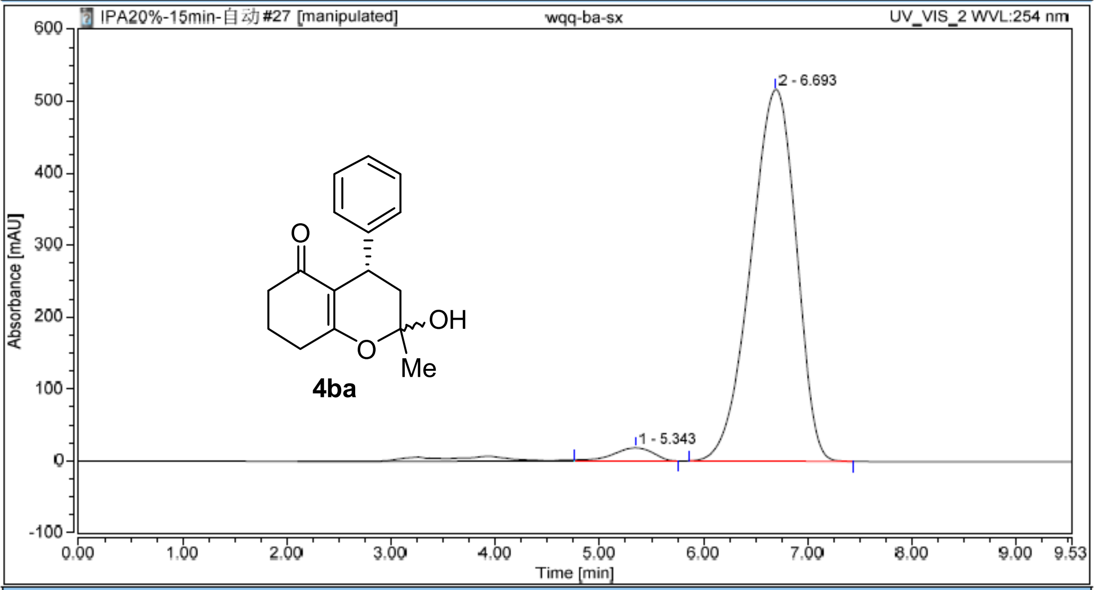

Integration Results

No.	Peak Name	Retention Time<br>min	Area<br>mAU*min	Height<br>mAU	Relative Area<br>%	Relative Height<br>%	Amount<br>n.a.
1		5.343	8.266	18.328	3.07	3.43	n.a.
2		6.693	260.680	516.682	96.93	96.57	n.a.
Total:			**268.947**	**535.011**	**100.00**	**100.00**	

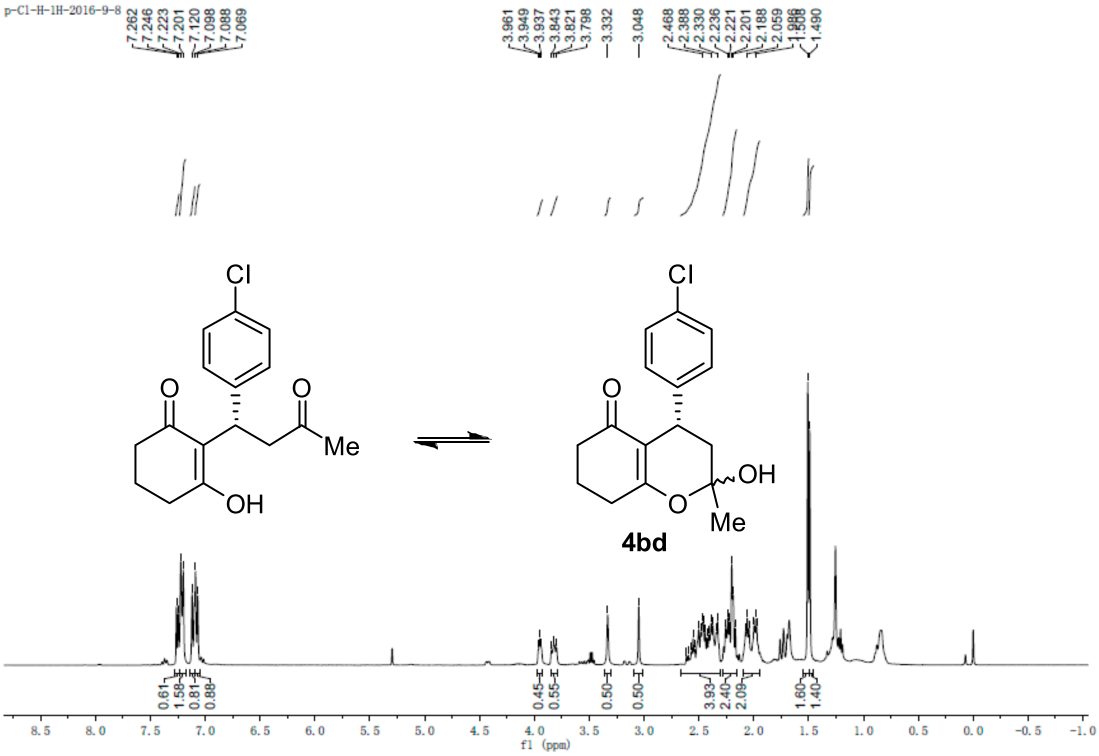
p-Cl-H-1H-2016-9-8
7.262 7.246 7.223 7.201 7.120 7.098 7.088 7.069
3.961 3.949 3.937 3.843 3.821 3.798
3.332
3.048
2.468 2.388 2.330 2.236 2.221 2.201 2.188 2.059
1.490
Cl
O
O
Me
OH
OH
Me
4bd
0.61 1.58 0.81 0.88
0.45 0.55
0.50
0.50
3.93 2.40 2.09
1.60 1.40
f1 (ppm)

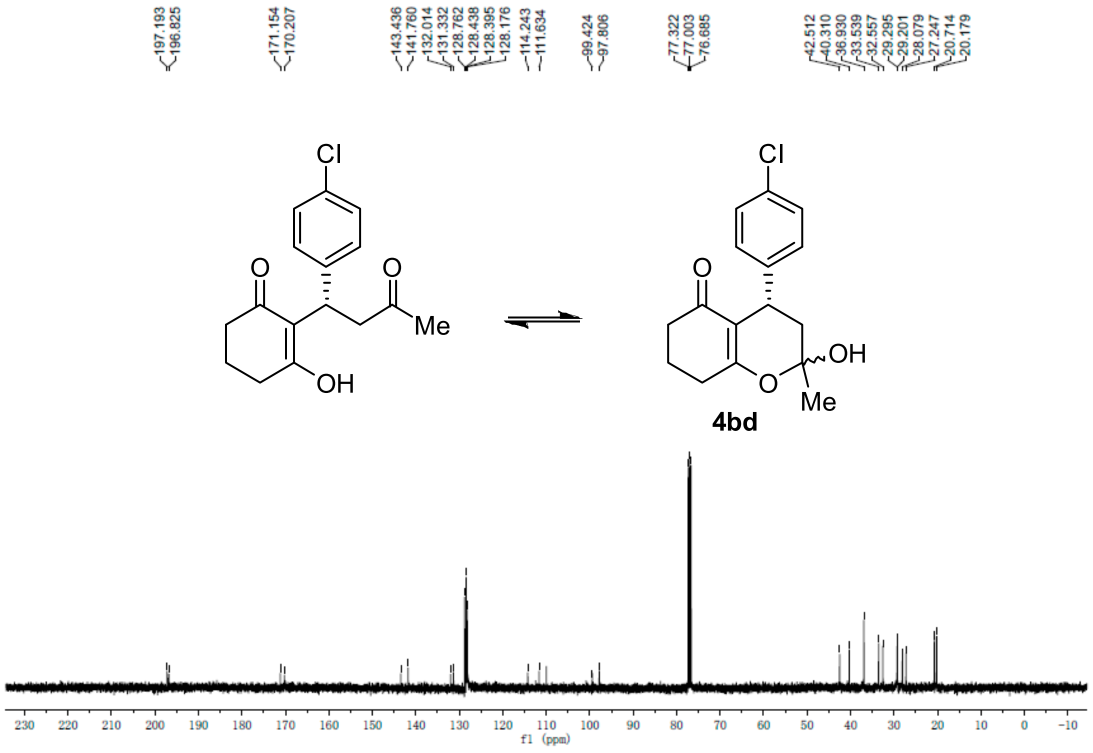
197.193 196.825
171.154 170.207
143.436 141.760 132.014 131.332 128.762 128.438 128.395 128.176
114.243 111.634
99.424 97.806
77.322 77.003 76.685
42.512 40.310 36.930 33.539 32.557 29.295 29.201 28.079 27.247 20.714 20.179
Cl
O
O
Me
OH
OH
Me
4bd
f1 (ppm)

Chromatogram

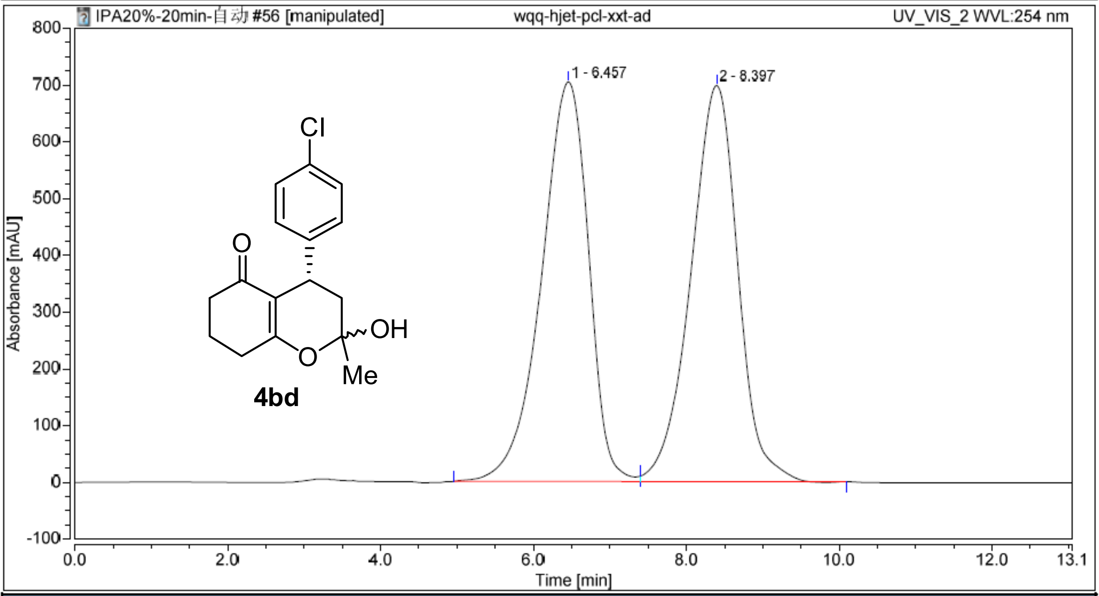

Integration Results

No.	Peak Name	Retention Time min	Area mAU*min	Height mAU	Relative Area %	Relative Height %	Amount n.a.
1		6.457	501.839	703.805	50.24	50.22	n.a.
2		8.397	496.974	697.668	49.76	49.78	n.a.
**Total:**			**998.813**	**1401.472**	**100.00**	**100.00**	

Chromatogram

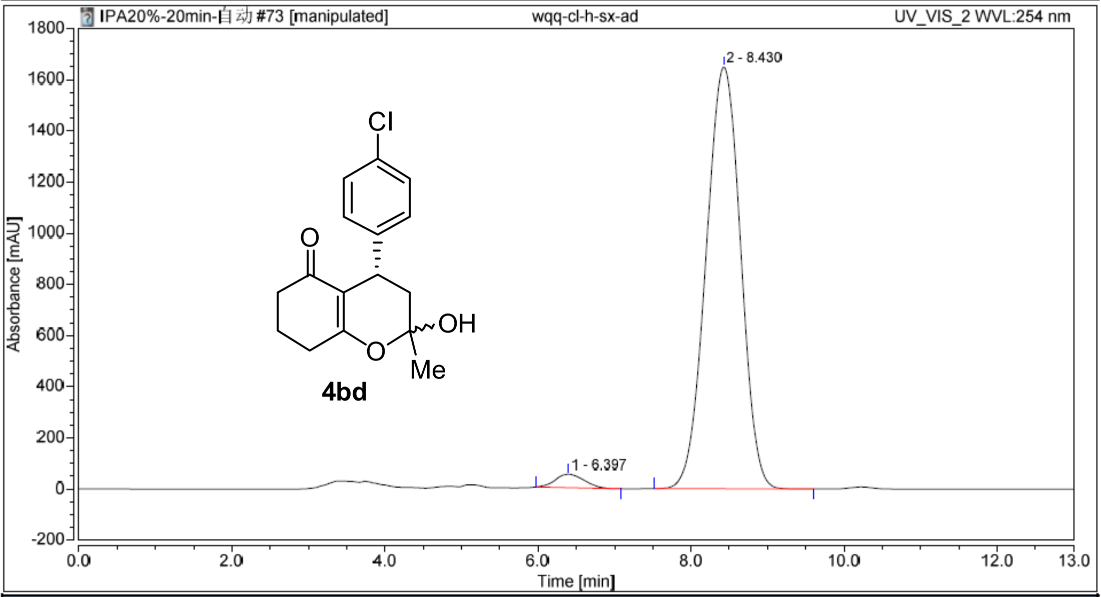

Integration Results

No.	Peak Name	Retention Time min	Area mAU*min	Height mAU	Relative Area %	Relative Height %	Amount n.a.
1		6.397	22.776	52.055	2.57	3.06	n.a.
2		8.430	862.634	1649.122	97.43	96.94	n.a.
**Total:**			**885.410**	**1701.176**	**100.00**	**100.00**	

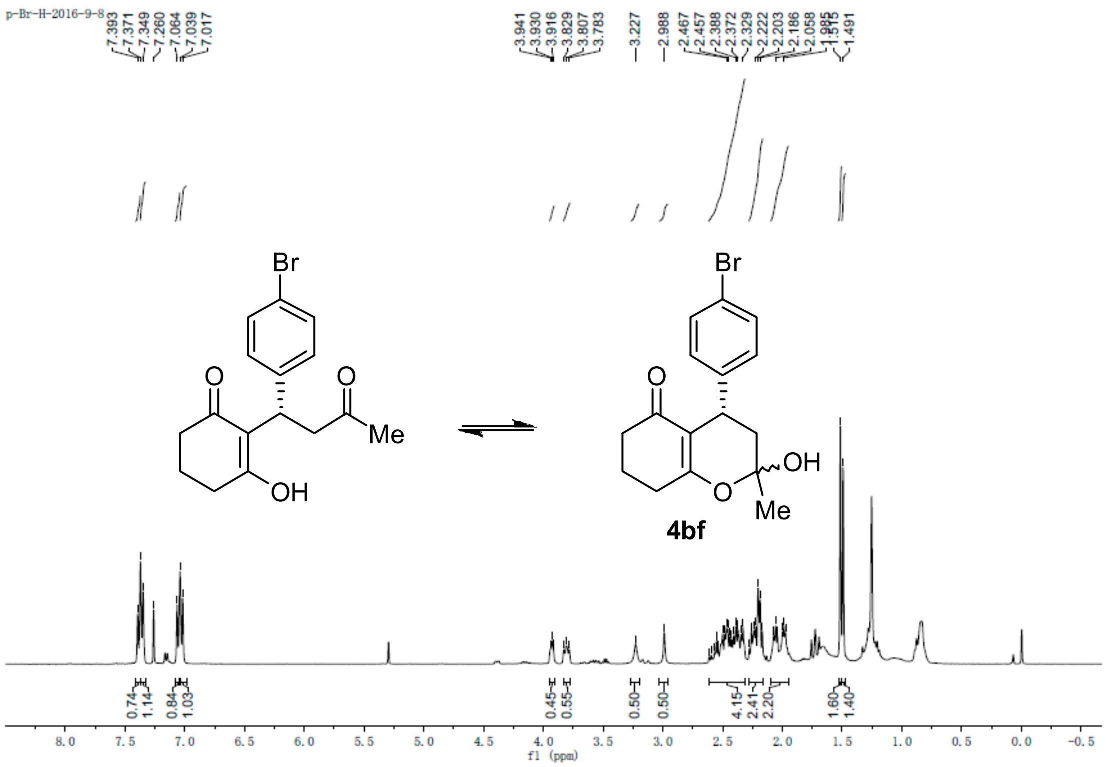
p-Br-H-2016-9-8
7.393
7.371
7.349
7.260
7.064
7.039
7.017
3.941
3.930
3.916
3.829
3.807
3.783
3.227
2.988
2.467
2.457
2.388
2.372
2.329
2.222
2.203
2.186
2.058
1.491
Br
O
O
Me
OH
OH
Me
4bf
0.74
1.14
0.84
1.03
0.45
0.55
0.50
0.50
4.15
2.41
2.20
1.60
1.40
8.0
7.5
7.0
6.5
6.0
5.5
5.0
4.5
4.0
3.5
3.0
2.5
2.0
1.5
1.0
0.5
0.0
-0.5
f1 (ppm)

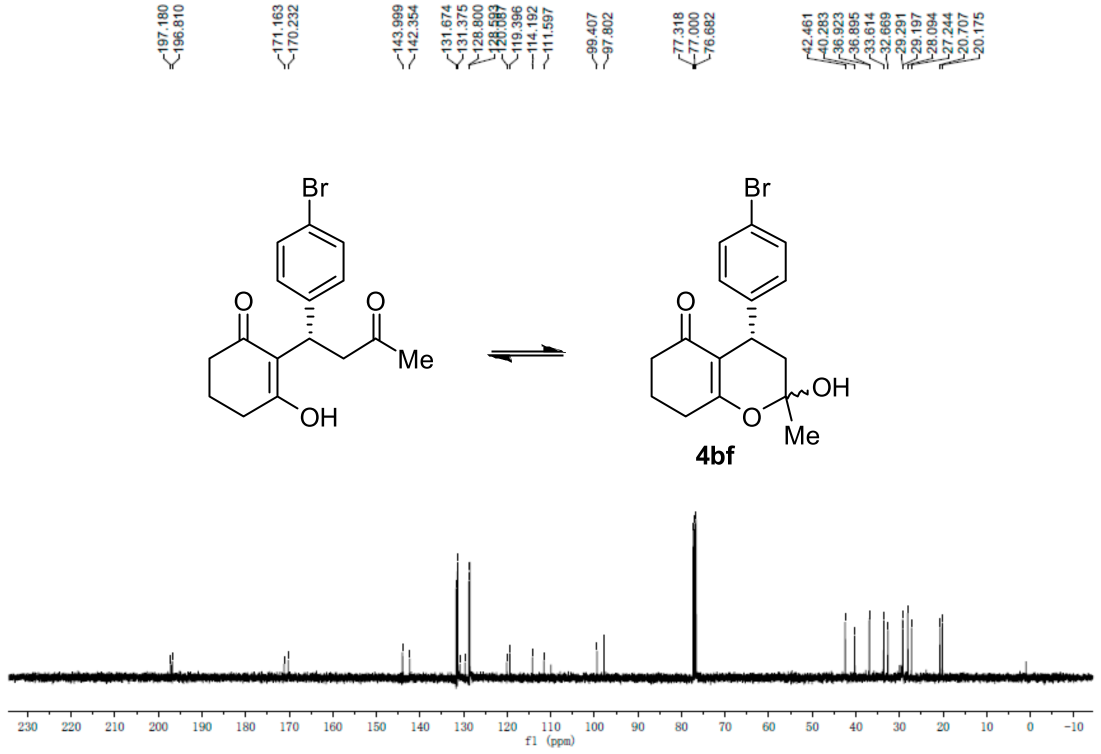
197.180
196.810
171.163
170.232
143.999
142.354
131.674
131.375
128.800
119.396
114.192
111.597
99.407
97.802
77.318
77.000
76.682
42.461
40.283
36.923
36.895
33.614
32.669
29.291
29.197
28.094
27.244
20.707
20.175
Br
O
O
Me
OH
OH
Me
4bf
230
220
210
200
190
180
170
160
150
140
130
120
110
100
90
80
70
60
50
40
30
20
10
0
-10
f1 (ppm)

Chromatogram

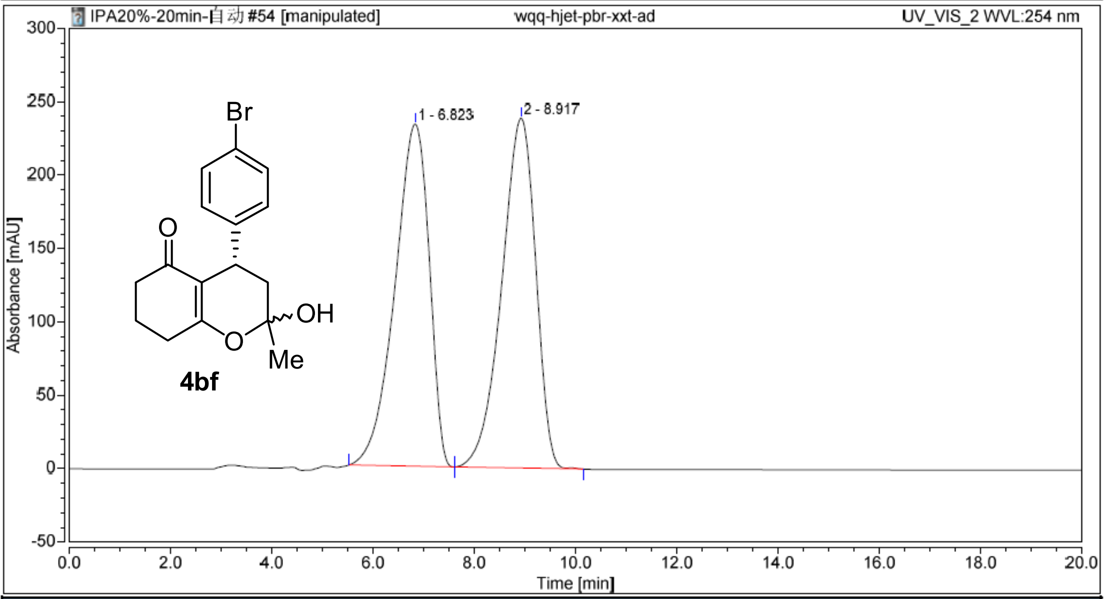

Integration Results

No.	Peak Name	Retention Time min	Area mAU*min	Height mAU	Relative Area %	Relative Height %	Amount n.a.
1		6.823	182.779	233.155	49.70	49.44	n.a.
2		8.917	184.960	238.416	50.30	50.56	n.a.
**Total:**			**367.740**	**471.571**	**100.00**	**100.00**	

Chromatogram

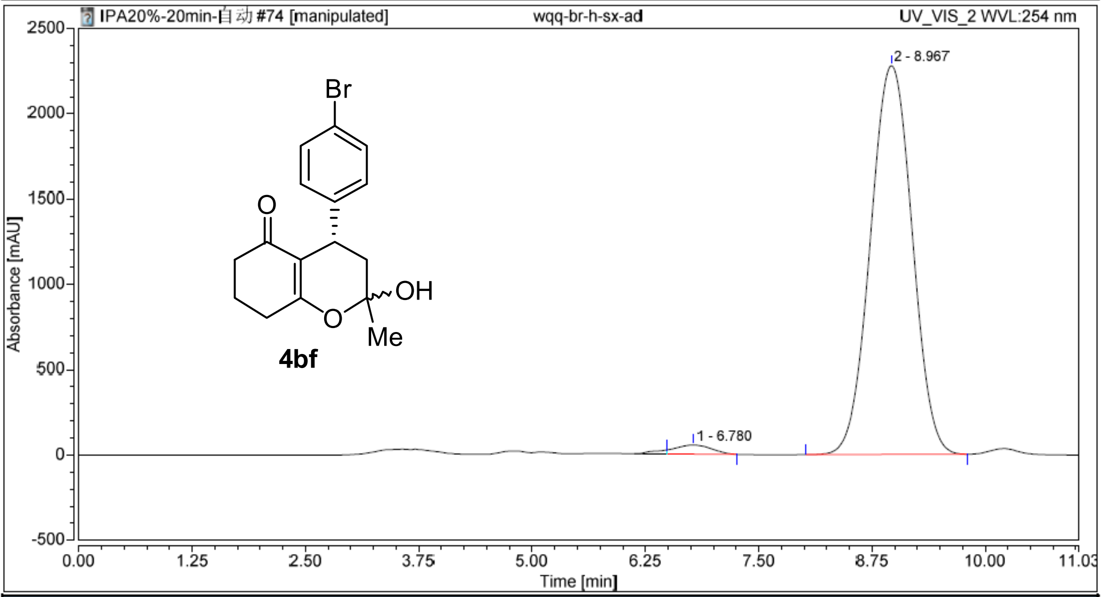

Integration Results

No.	Peak Name	Retention Time min	Area mAU*min	Height mAU	Relative Area %	Relative Height %	Amount n.a.
1		6.780	24.403	53.278	1.97	2.29	n.a.
2		8.967	1214.027	2277.581	98.03	97.71	n.a.
**Total:**			**1238.429**	**2330.859**	**100.00**	**100.00**	

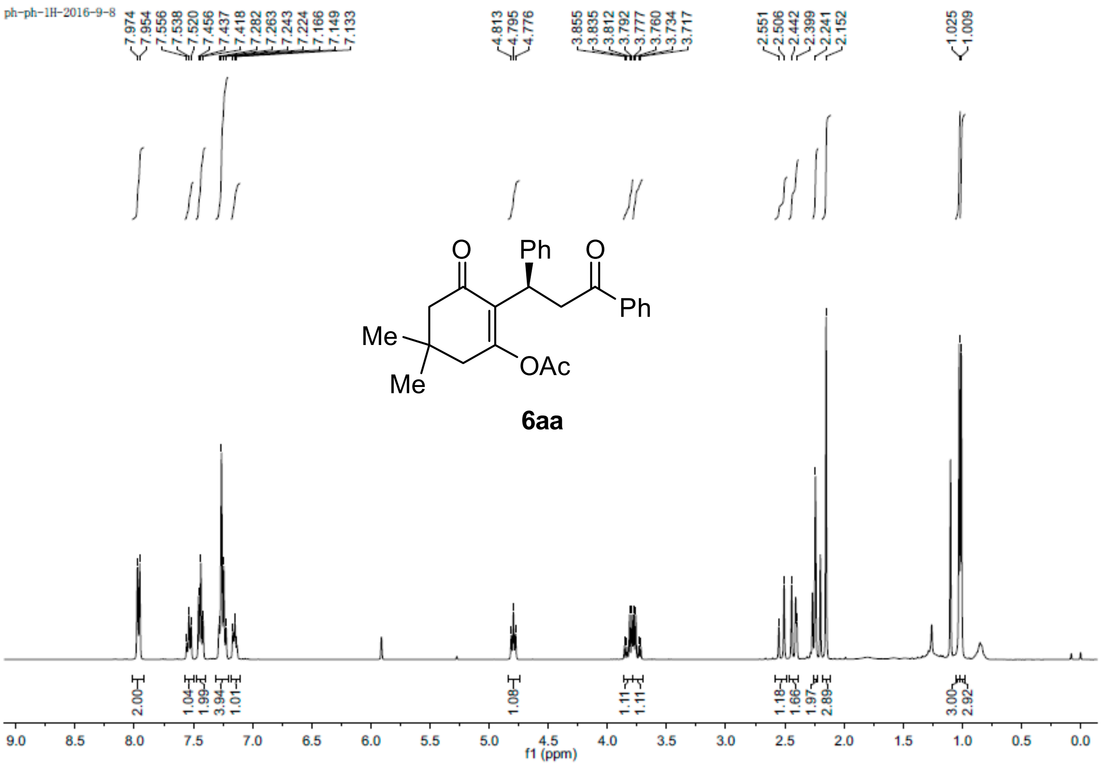
ph-ph-1H-2016-9-8
6aa
f1 (ppm)

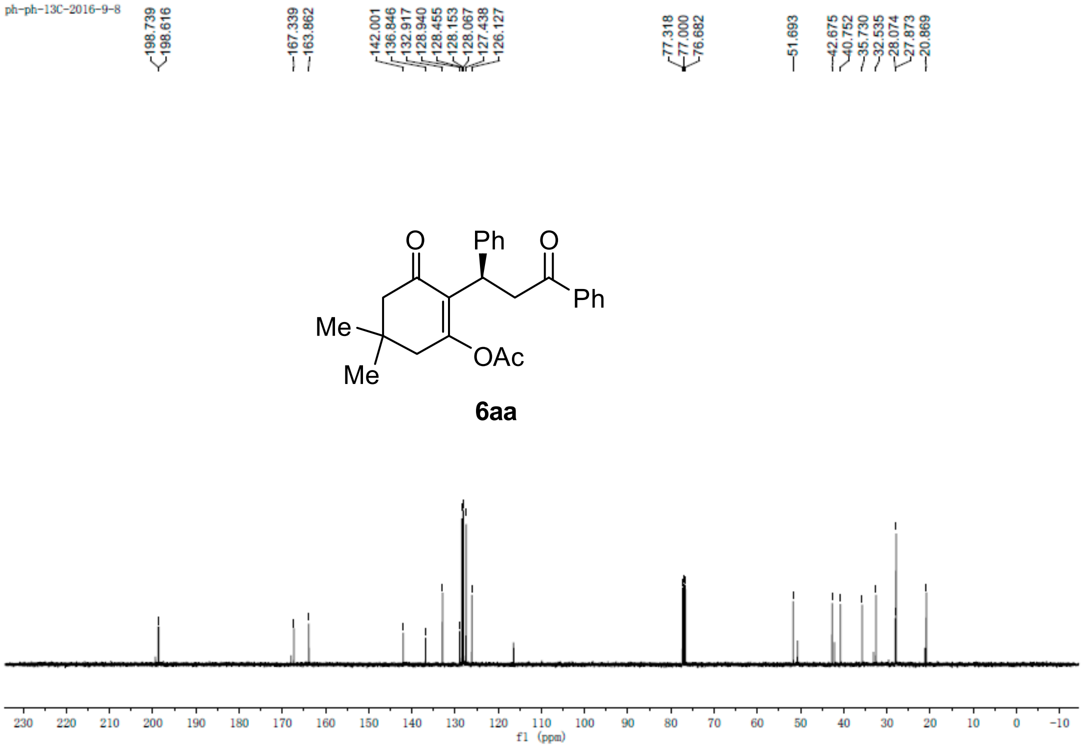
ph-ph-13C-2016-9-8
6aa
f1 (ppm)

Chromatogram

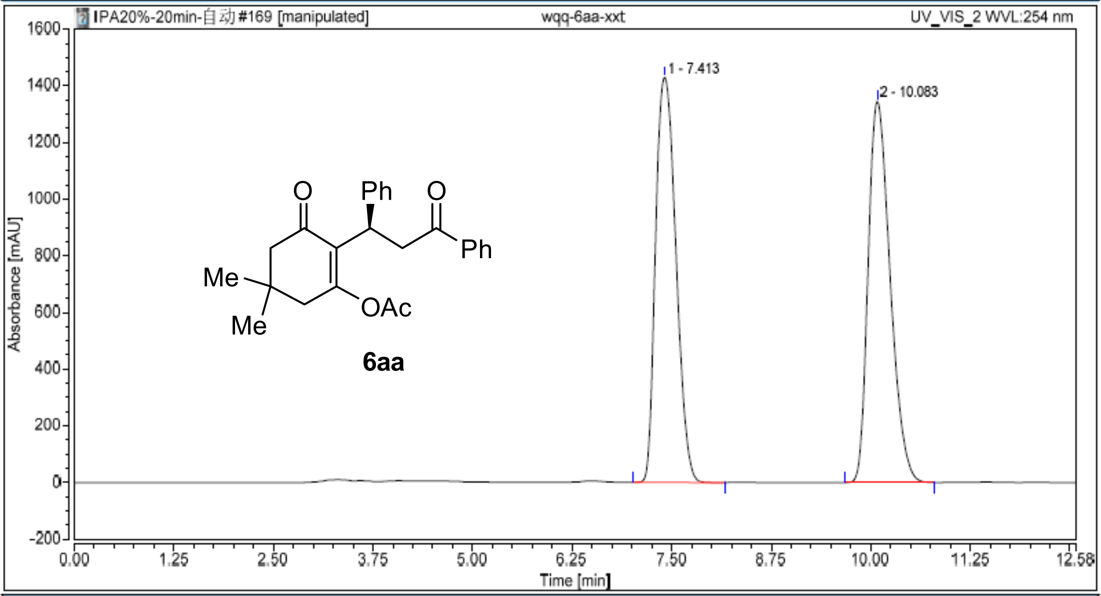

Integration Results

No.	Peak Name	Retention Time min	Area mAU*min	Height mAU	Relative Area %	Relative Height %	Amount n.a.
1		7.413	422.493	1429.808	49.54	51.52	n.a.
2		10.083	430.411	1345.309	50.46	48.48	n.a.
Total:			**852.904**	**2775.116**	**100.00**	**100.00**	

Chromatogram

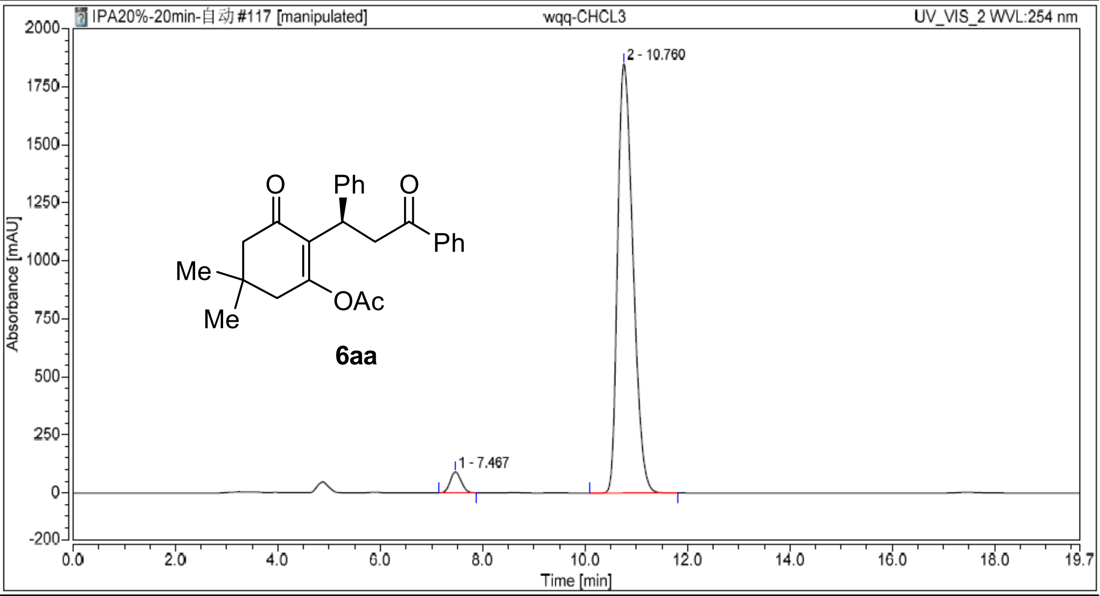

Integration Results

No.	Peak Name	Retention Time min	Area mAU*min	Height mAU	Relative Area %	Relative Height %	Amount n.a.
1		7.467	22.380	89.971	3.37	4.64	n.a.
2		10.760	642.517	1847.551	96.63	95.36	n.a.
Total:			**664.897**	**1937.522**	**100.00**	**100.00**	

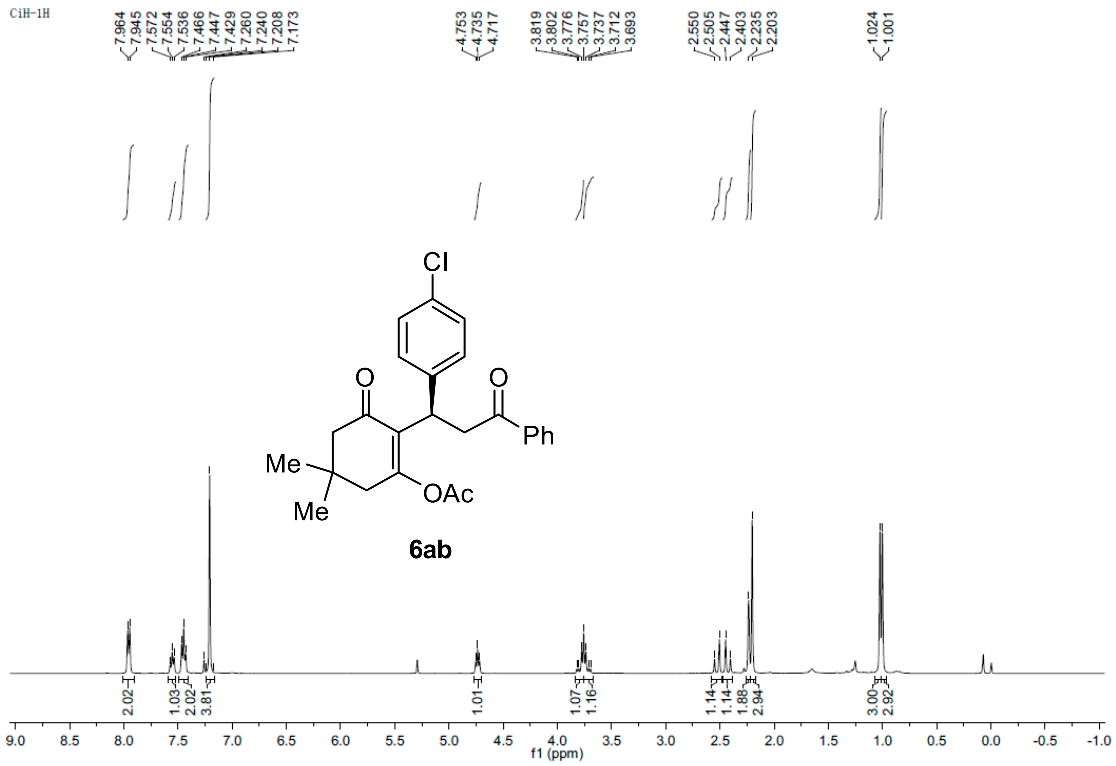
CiH-1H
7.964
7.945
7.572
7.554
7.536
7.466
7.447
7.429
7.260
7.240
7.208
7.173
4.753
4.735
4.717
3.819
3.802
3.776
3.757
3.737
3.712
3.693
2.550
2.505
2.447
2.403
2.235
2.203
1.024
1.001
Cl
O
O
Ph
Me
Me
OAc
6ab
2.02
1.03
2.02
3.81
1.01
1.07
1.16
1.14
1.14
1.88
2.94
3.00
2.92
f1 (ppm)

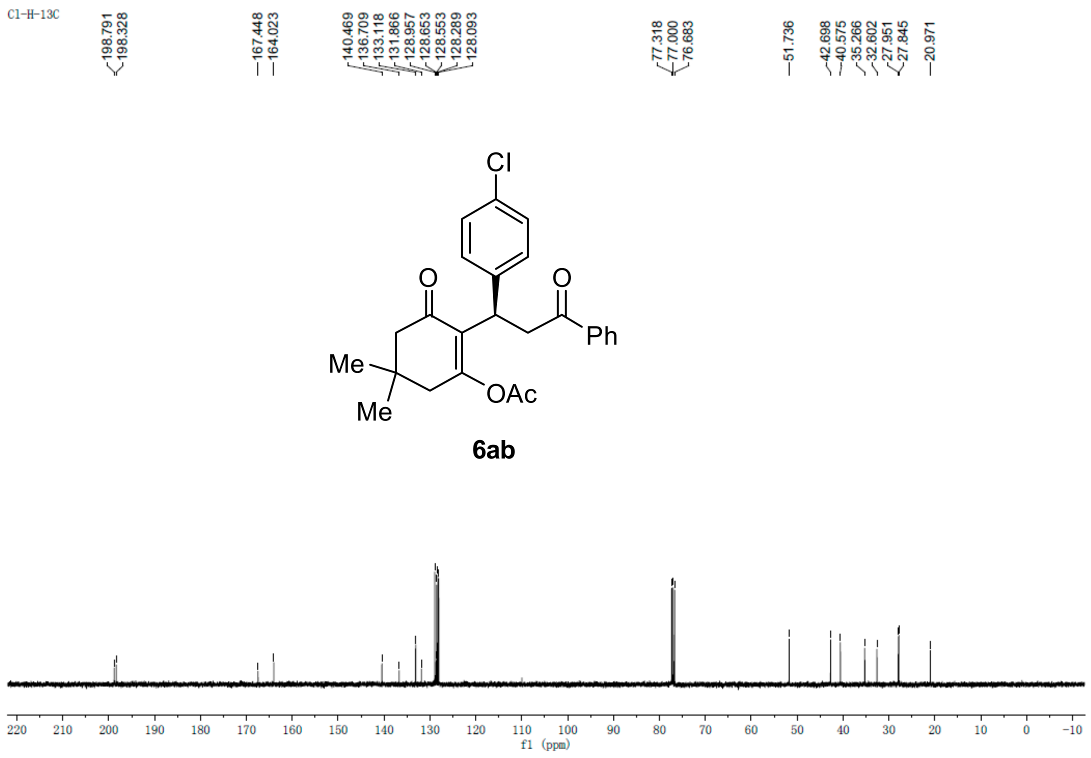
Cl-H-13C
198.791
198.328
167.448
164.023
140.469
136.709
133.118
131.866
128.957
128.653
128.553
128.289
128.093
77.318
77.000
76.683
51.736
42.698
40.575
35.266
32.602
27.951
27.845
20.971
Cl
O
O
Ph
Me
Me
OAc
6ab
f1 (ppm)

Chromatogram

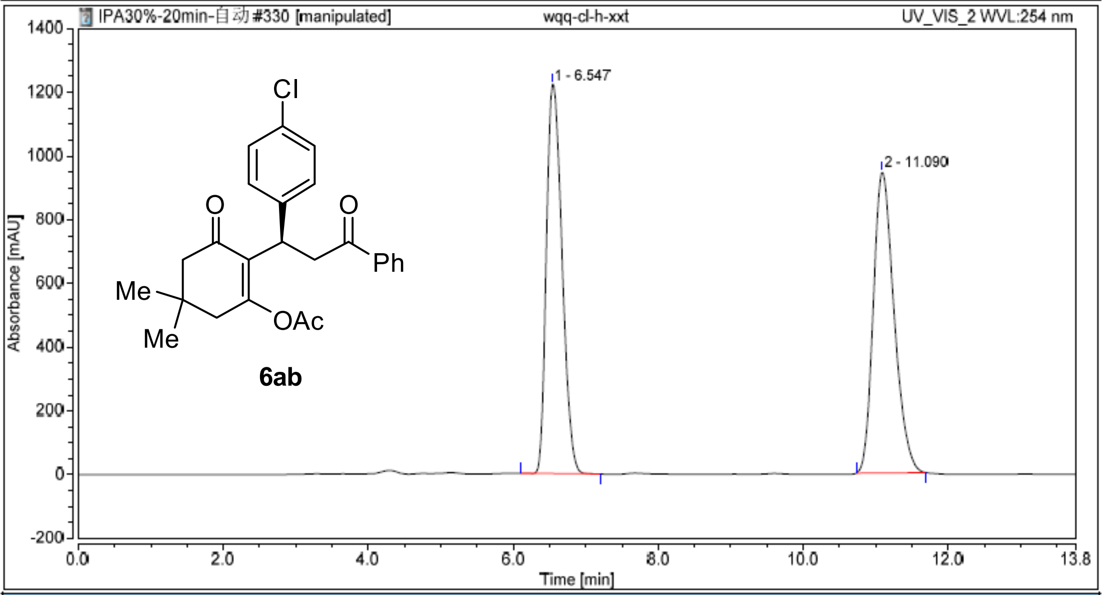

Integration Results

No.	Peak Name	Retention Time min	Area mAU*min	Height mAU	Relative Area %	Relative Height %	Amount n.a.
1		6.547	304.607	1222.204	49.26	56.39	n.a.
2		11.090	313.699	945.072	50.74	43.61	n.a.
Total:			**618.306**	**2167.276**	**100.00**	**100.00**	

Chromatogram

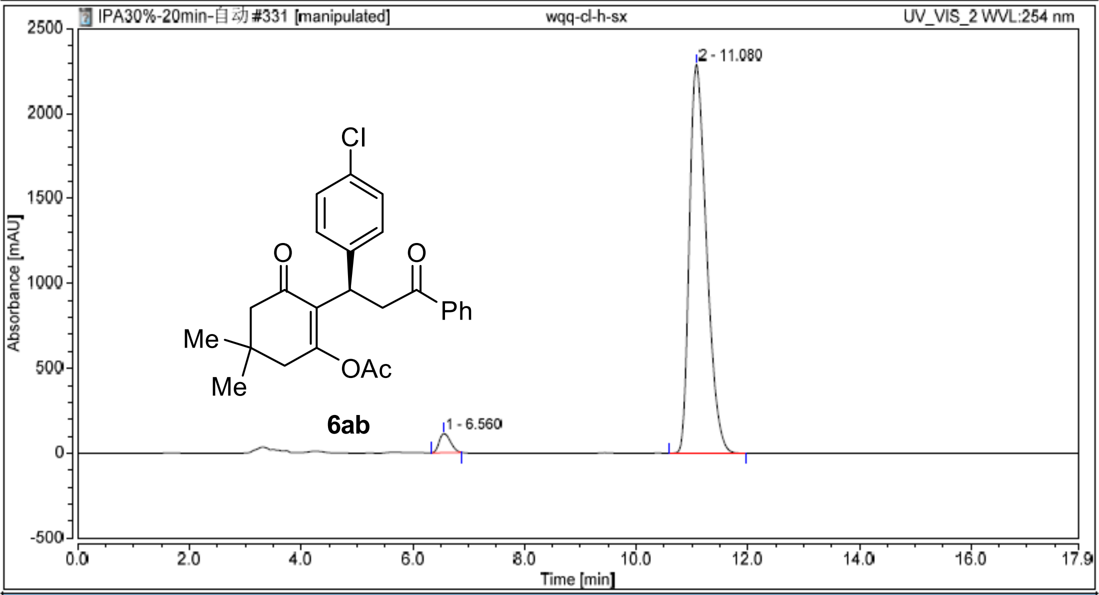

Integration Results

No.	Peak Name	Retention Time min	Area mAU*min	Height mAU	Relative Area %	Relative Height %	Amount n.a.
1		6.560	26.286	112.446	3.13	4.69	n.a.
2		11.080	813.437	2287.430	96.87	95.31	n.a.
Total:			**839.724**	**2399.875**	**100.00**	**100.00**	

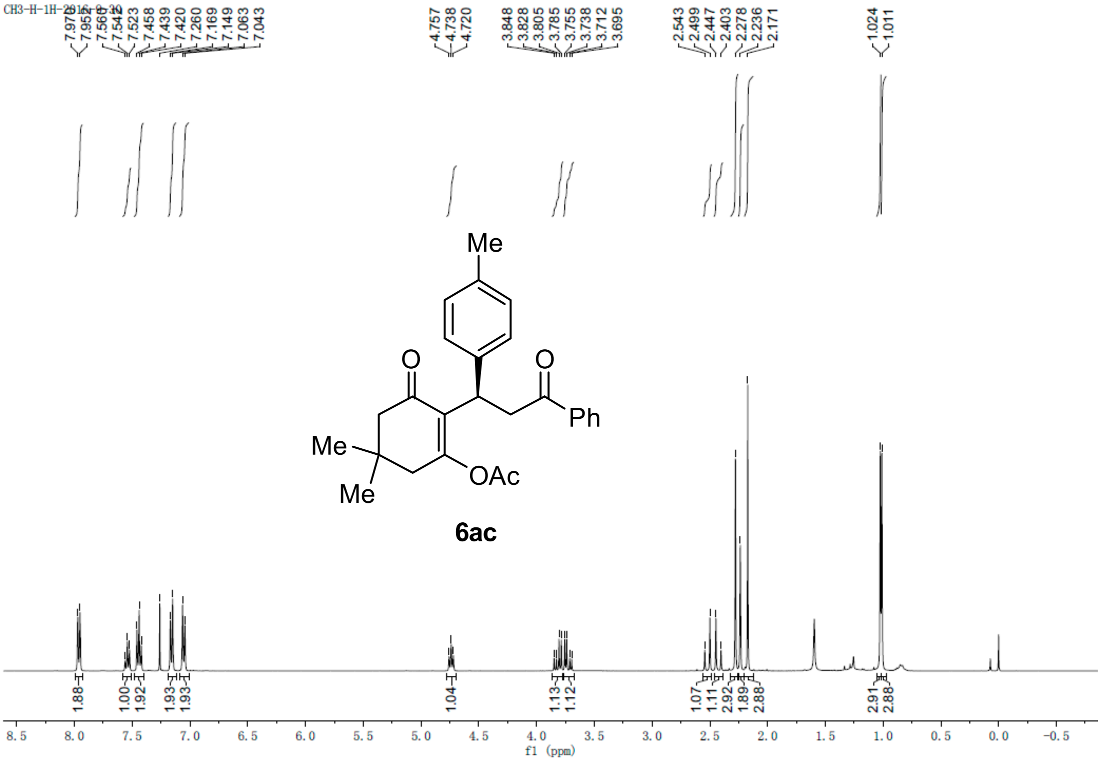
CH3-H-1H-2016-9-30
7.971
7.958
7.560
7.542
7.523
7.458
7.439
7.420
7.260
7.169
7.149
7.063
7.043
4.757
4.738
4.720
3.848
3.828
3.805
3.785
3.755
3.738
3.712
3.695
2.543
2.499
2.447
2.403
2.278
2.236
2.171
1.024
1.011
Me
O
O
Ph
Me
OAc
Me
6ac
1.88
1.00
1.92
1.93
1.93
1.04
1.13
1.12
1.07
1.11
2.92
1.89
2.88
2.91
2.88
f1 (ppm)

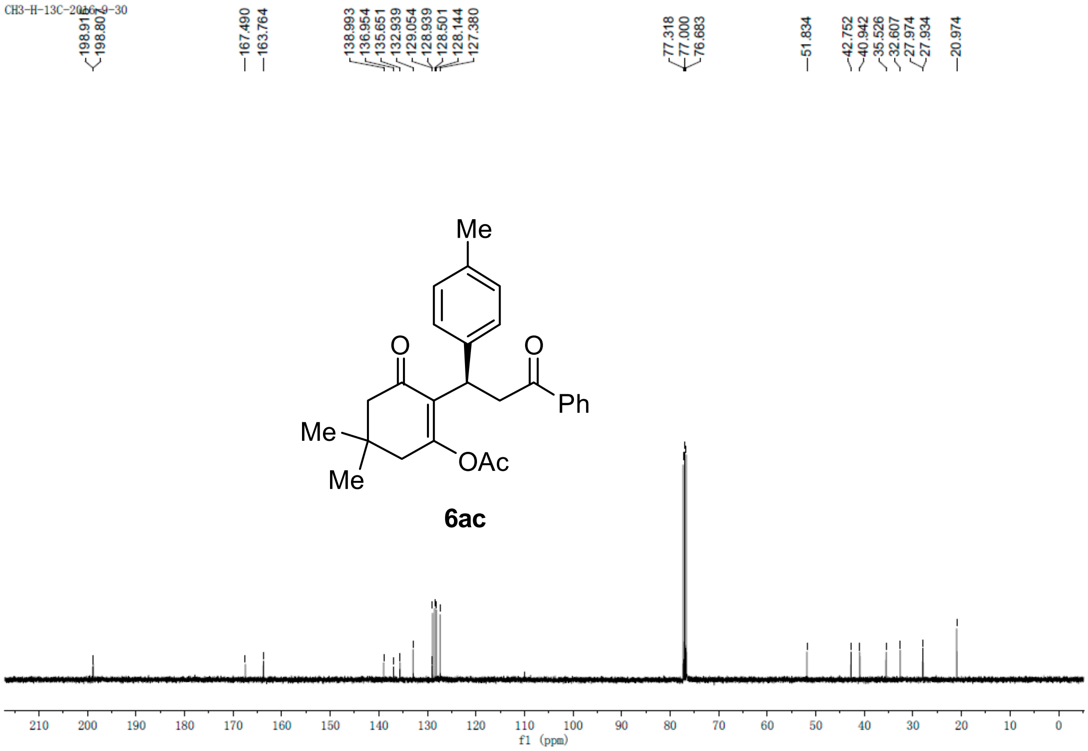
CH3-H-13C-2016-9-30
198.915
198.807
167.490
163.764
138.993
136.954
135.651
132.939
129.054
128.939
128.501
128.144
127.380
77.318
77.000
76.683
51.834
42.752
40.942
35.526
32.607
27.974
27.934
20.974
Me
O
O
Ph
Me
OAc
Me
6ac
f1 (ppm)

Chromatogram

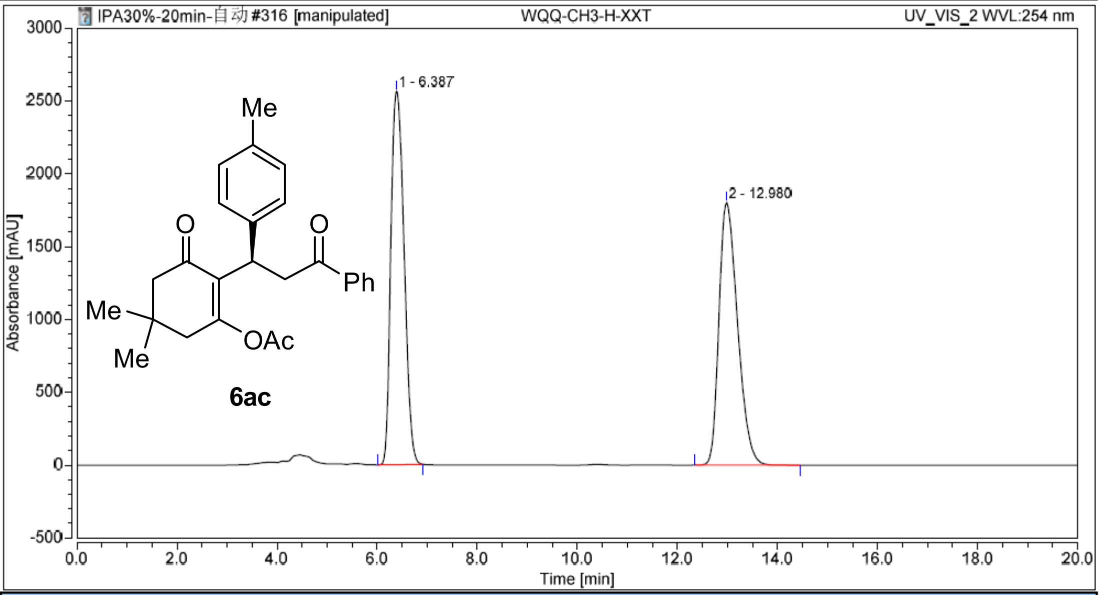

Integration Results

No.	Peak Name	Retention Time<br>min	Area<br>mAU*min	Height<br>mAU	Relative Area<br>%	Relative Height<br>%	Amount<br>n.a.
1		6.387	781.505	2564.376	50.25	58.72	n.a.
2		12.980	773.582	1802.842	49.75	41.28	n.a.
**Total:**			**1555.088**	**4367.218**	**100.00**	**100.00**	

Chromatogram

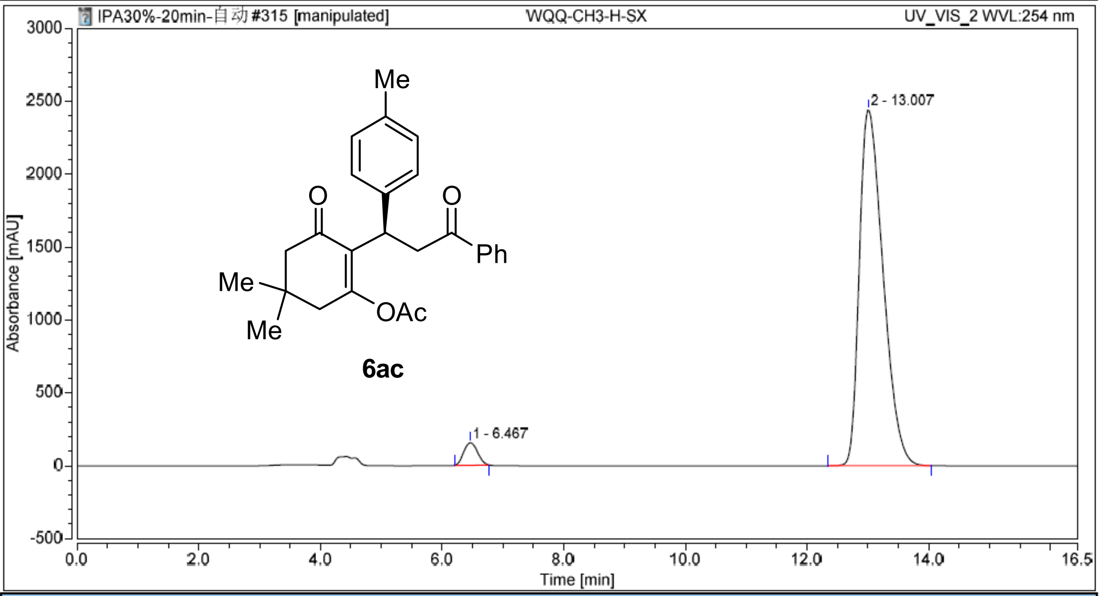

Integration Results

No.	Peak Name	Retention Time<br>min	Area<br>mAU*min	Height<br>mAU	Relative Area<br>%	Relative Height<br>%	Amount<br>n.a.
1		6.467	40.762	154.775	3.57	5.96	n.a.
2		13.007	1102.012	2442.184	96.43	94.04	n.a.
**Total:**			**1142.774**	**2596.959**	**100.00**	**100.00**	

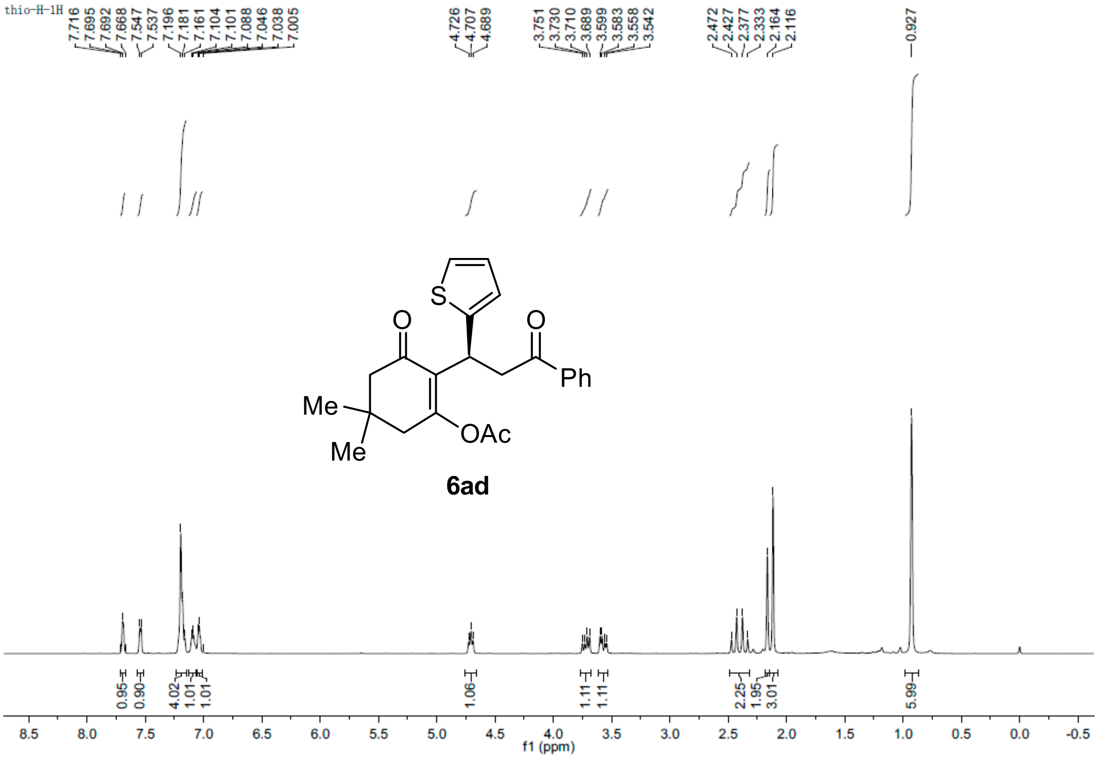
thio-H-1H
7.716 7.695 7.692 7.668 7.547 7.537 7.196 7.181 7.161 7.104 7.101 7.088 7.046 7.038 7.005
4.726 4.707 4.689
3.751 3.730 3.710 3.689 3.599 3.583 3.558 3.542
2.472 2.427 2.377 2.333 2.164 2.116
0.927
O
S
O
Ph
Me
Me
OAc
6ad
0.95 0.90 4.02 1.01 1.01 1.06 1.11 1.11 2.25 1.95 3.01 5.99
f1 (ppm)

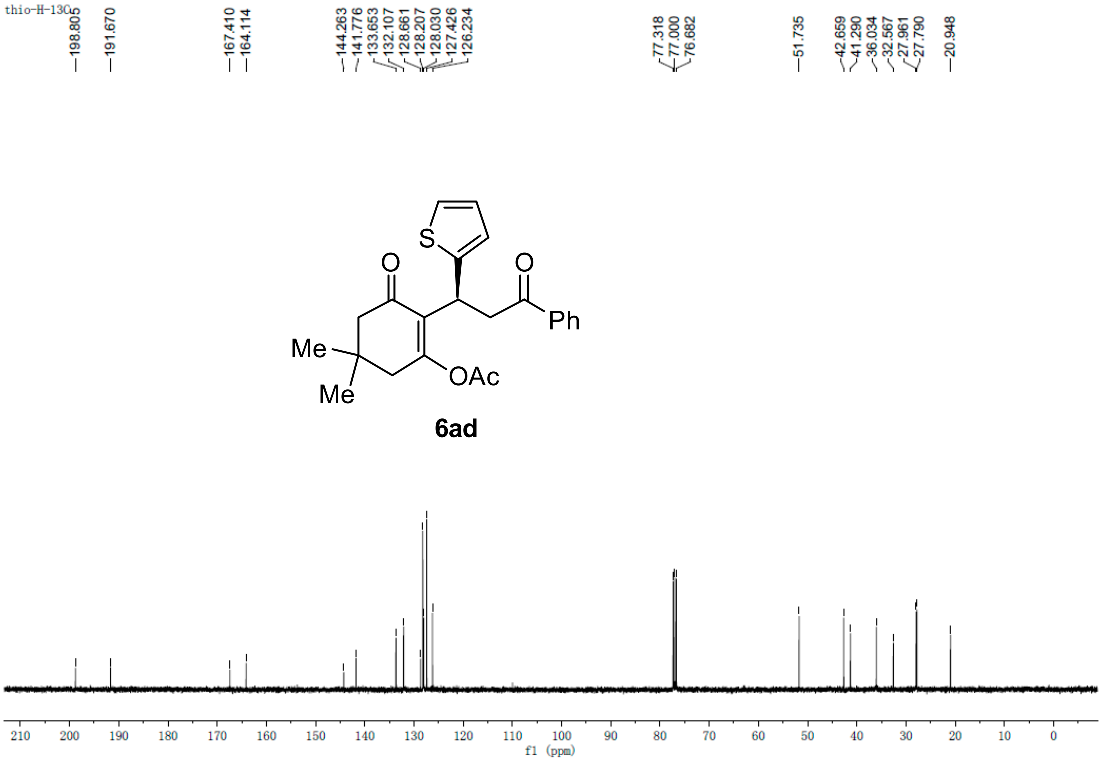
thio-H-13C
198.805
191.670
167.410 164.114
144.263 141.776 133.653 132.107 128.661 128.207 128.030 127.426 126.234
77.318 77.000 76.682
51.735
42.659 41.290 36.034 32.567 27.961 27.790
20.948
O
S
O
Ph
Me
Me
OAc
6ad
f1 (ppm)

Chromatogram

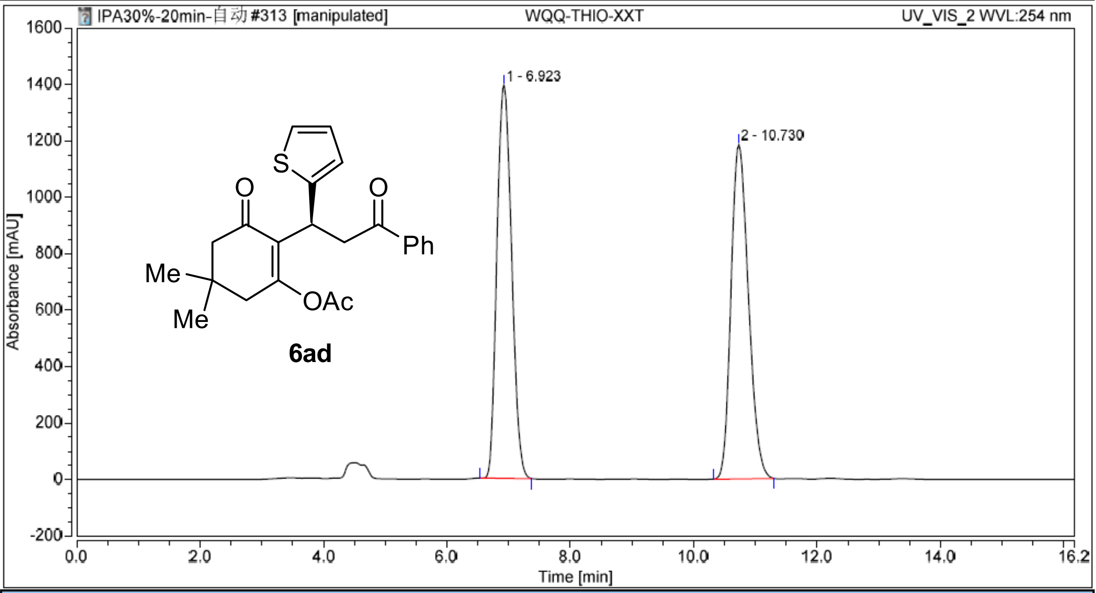

Integration Results

No.	Peak Name	Retention Time min	Area mAU*min	Height mAU	Relative Area %	Relative Height %	Amount n.a.
1		6.923	384.939	1393.187	49.84	54.04	n.a.
2		10.730	387.445	1185.010	50.16	45.96	n.a.
**Total:**			**772.384**	**2578.197**	**100.00**	**100.00**	

Chromatogram

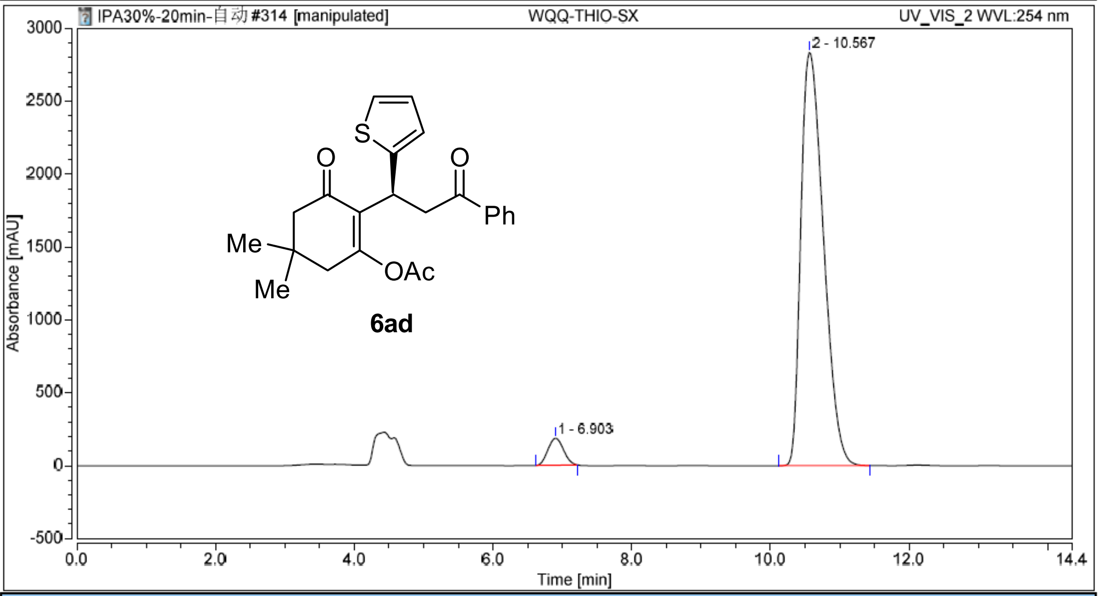

Integration Results

No.	Peak Name	Retention Time min	Area mAU*min	Height mAU	Relative Area %	Relative Height %	Amount n.a.
1		6.903	47.766	185.015	4.28	6.13	n.a.
2		10.567	1069.042	2835.280	95.72	93.87	n.a.
**Total:**			**1116.808**	**3020.294**	**100.00**	**100.00**	

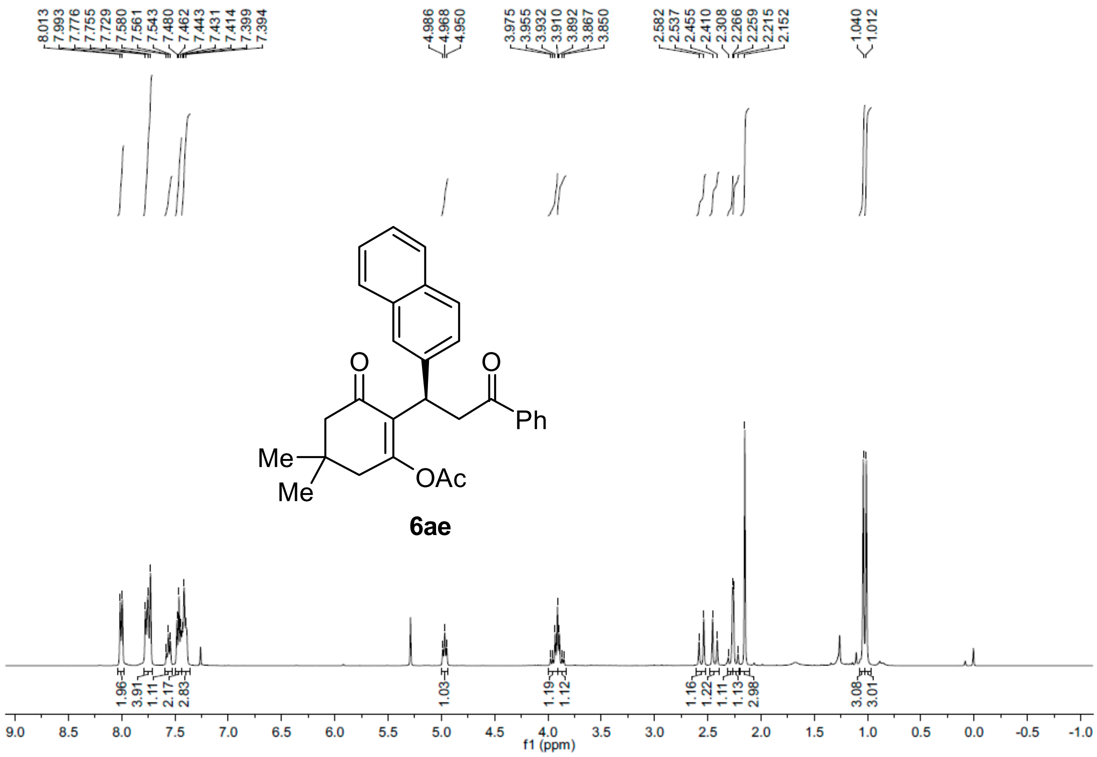

6ae

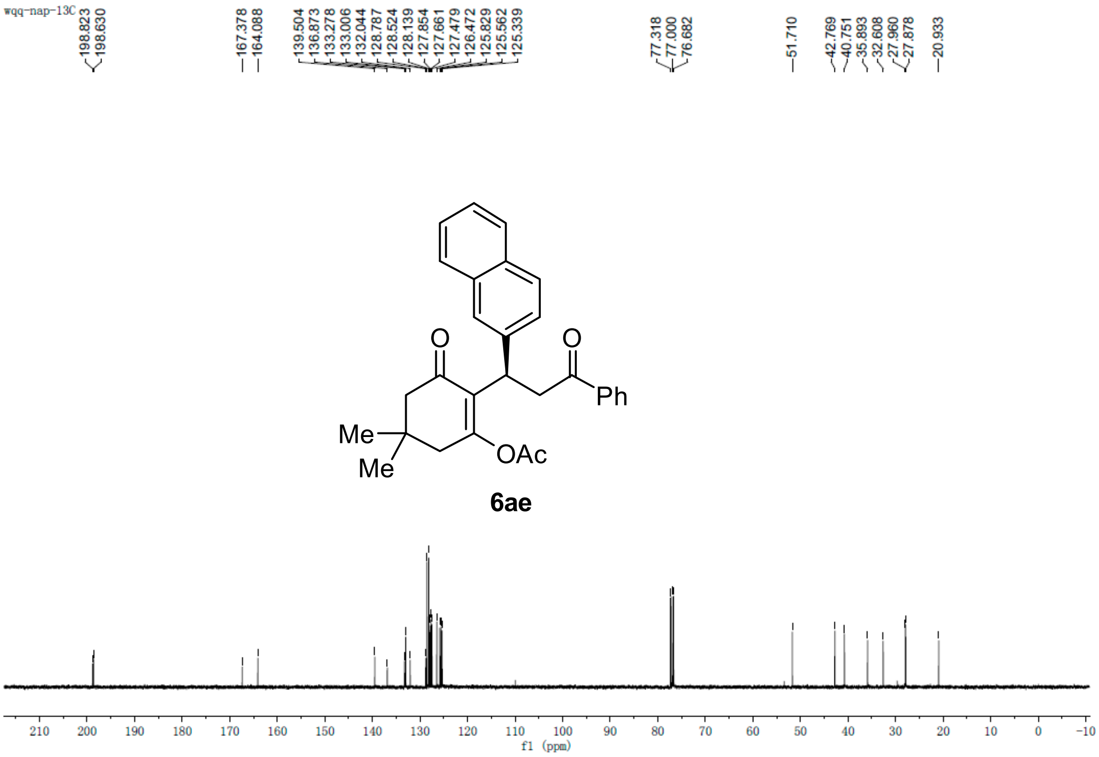

6ae

Chromatogram

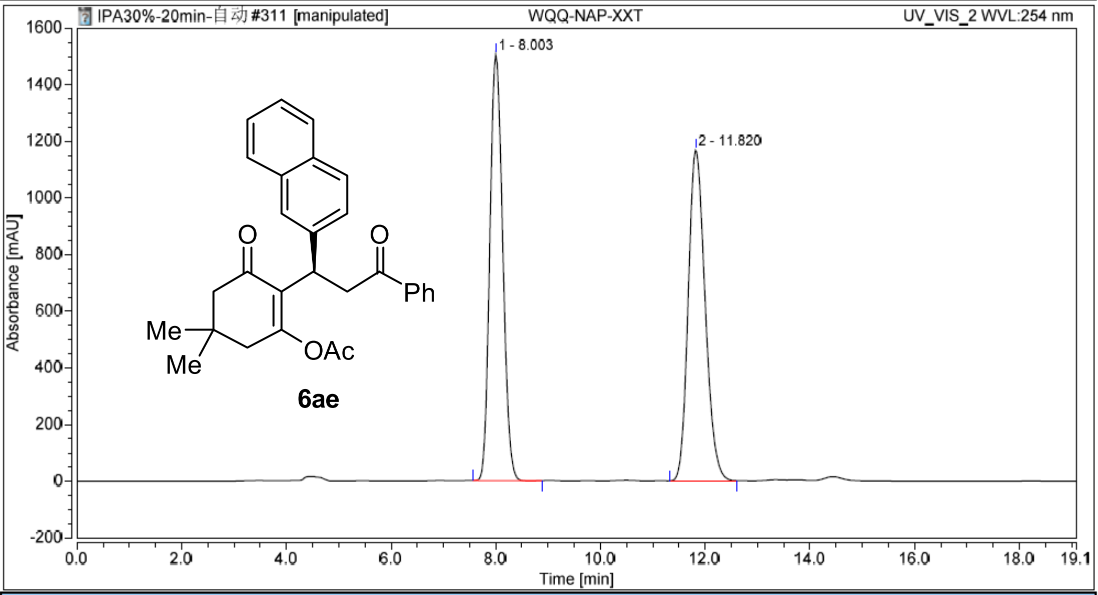

Integration Results

No.	Peak Name	Retention Time min	Area mAU*min	Height mAU	Relative Area %	Relative Height %	Amount n.a.
1		8.003	427.487	1504.954	49.42	56.27	n.a.
2		11.820	437.549	1169.473	50.58	43.73	n.a.
**Total:**			**865.036**	**2674.426**	**100.00**	**100.00**	

Chromatogram

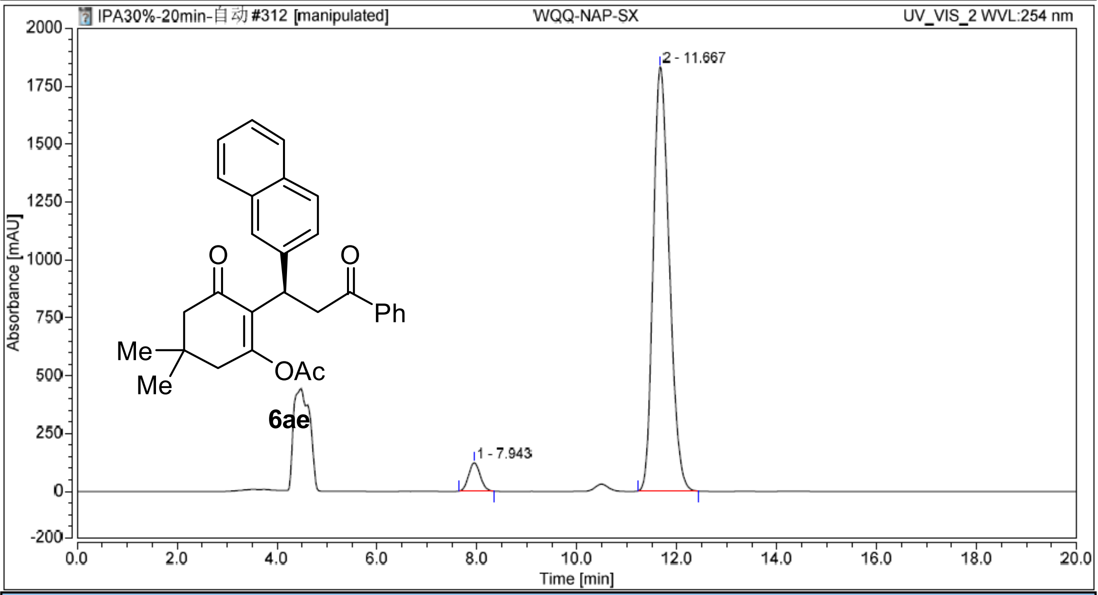

Integration Results

No.	Peak Name	Retention Time min	Area mAU*min	Height mAU	Relative Area %	Relative Height %	Amount n.a.
1		7.943	32.975	122.205	4.61	6.26	n.a.
2		11.667	682.286	1830.850	95.39	93.74	n.a.
**Total:**			**715.261**	**1953.056**	**100.00**	**100.00**	

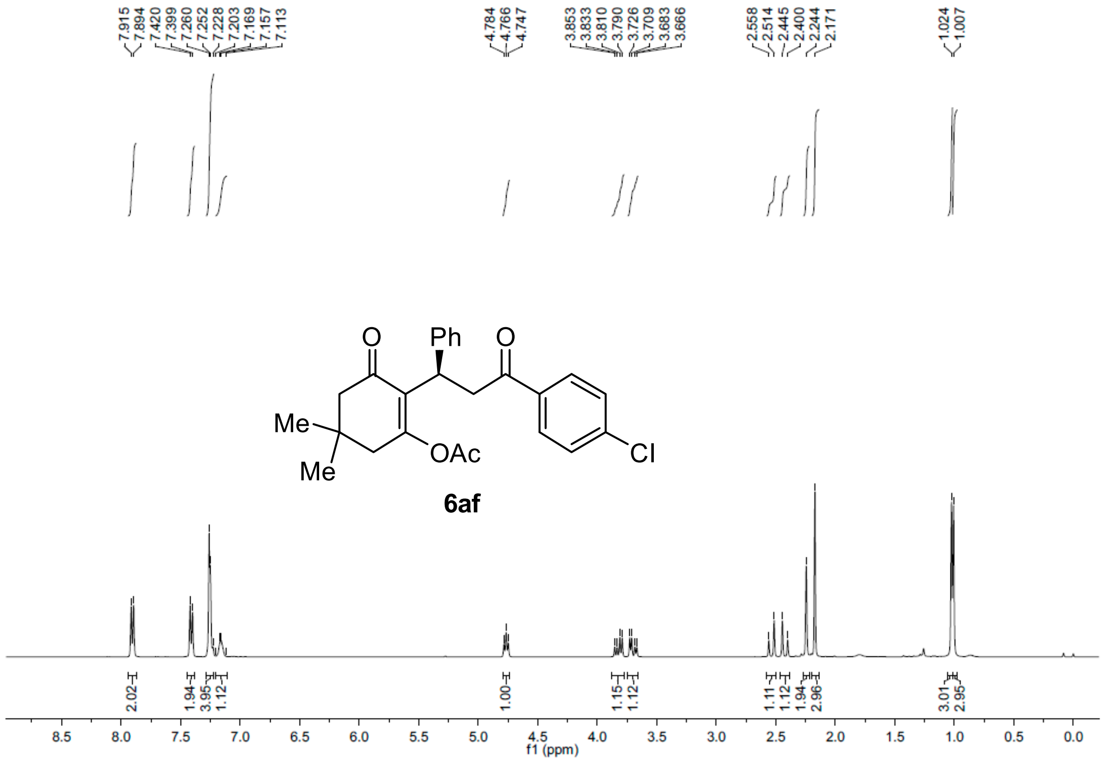

6af

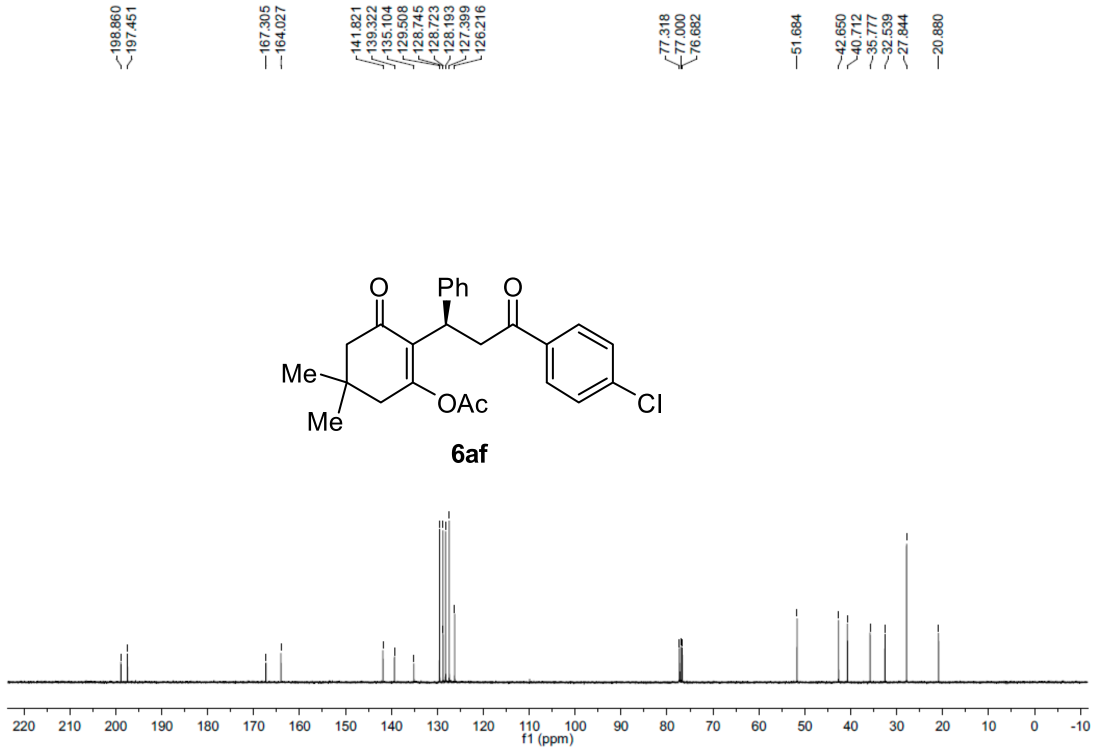

6af

Chromatogram

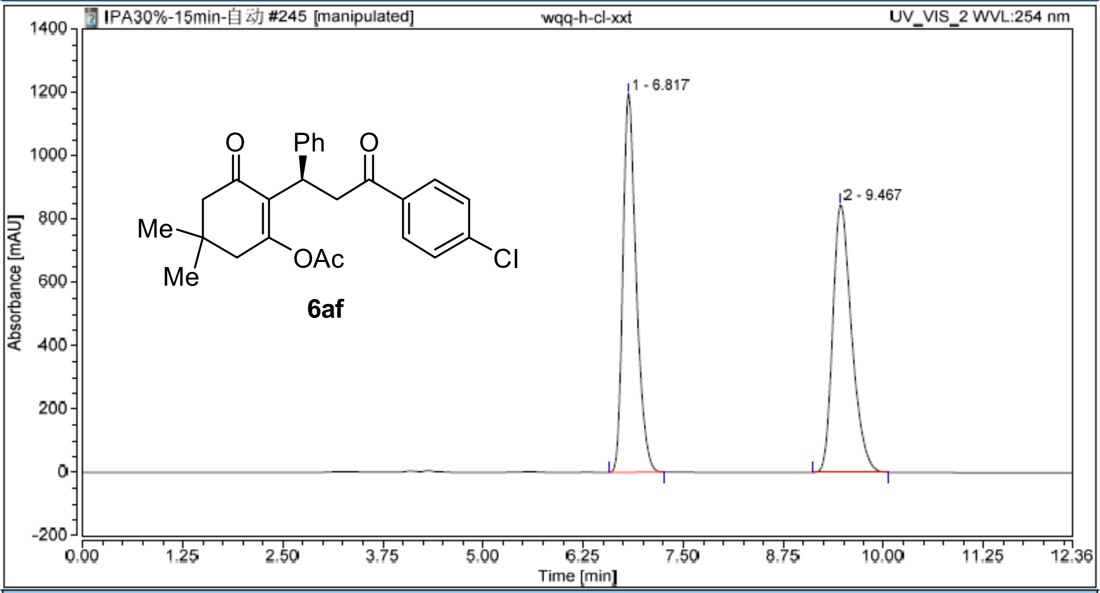

Integration Results

No.	Peak Name	Retention Time min	Area mAU*min	Height mAU	Relative Area %	Relative Height %	Amount n.a.
1		6.817	230.318	1196.055	49.94	58.54	n.a.
2		9.467	230.875	847.232	50.06	41.46	n.a.
Total:			**461.193**	**2043.286**	**100.00**	**100.00**	

Chromatogram

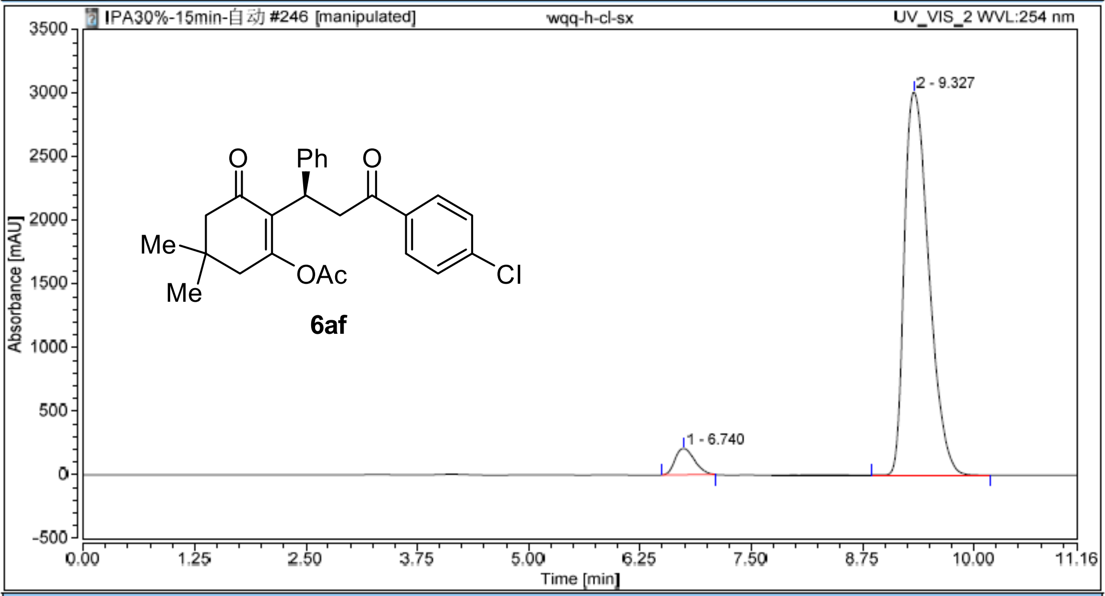

Integration Results

No.	Peak Name	Retention Time min	Area mAU*min	Height mAU	Relative Area %	Relative Height %	Amount n.a.
1		6.740	52.233	207.658	5.16	6.45	n.a.
2		9.327	959.221	3011.244	94.84	93.55	n.a.
Total:			**1011.454**	**3218.902**	**100.00**	**100.00**	

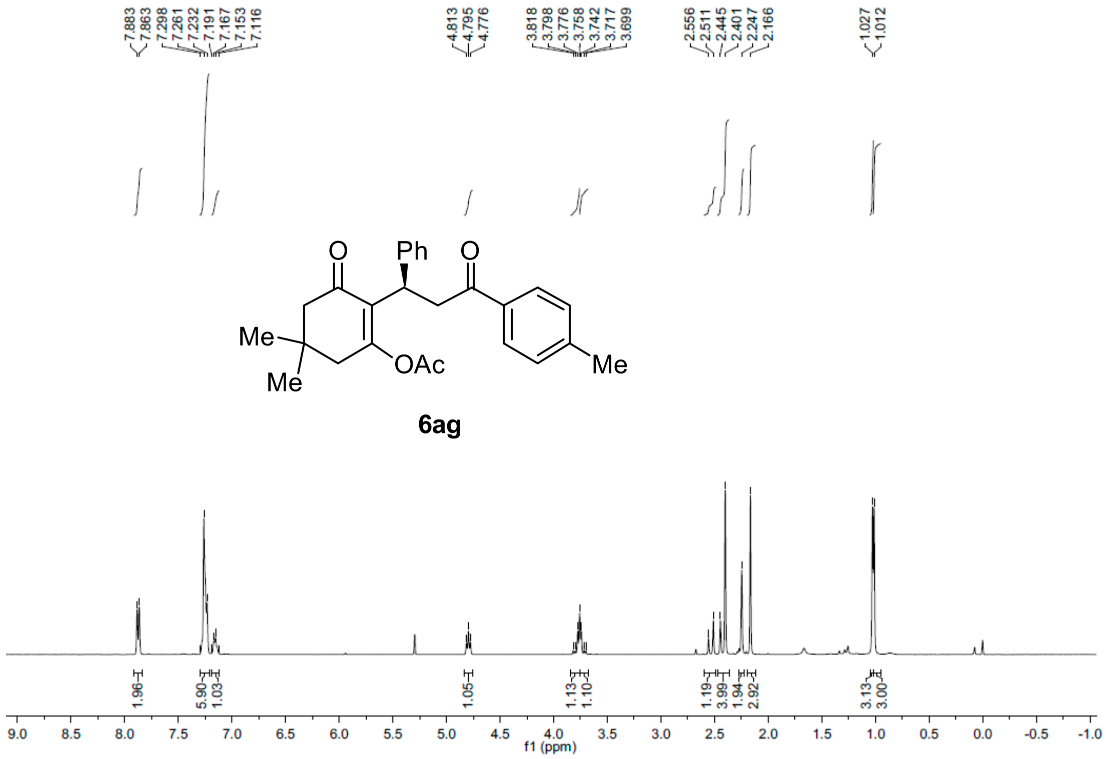
7.883
7.863
7.298
7.261
7.232
7.191
7.167
7.153
7.116
4.813
4.795
4.776
3.818
3.798
3.776
3.758
3.742
3.717
3.699
2.556
2.511
2.445
2.401
2.247
2.166
1.027
1.012
O
Ph
O
Me
Me
OAc
Me
6ag
1.96
5.90
1.03
1.05
1.13
1.10
1.19
3.99
1.94
2.92
3.13
3.00
f1 (ppm)

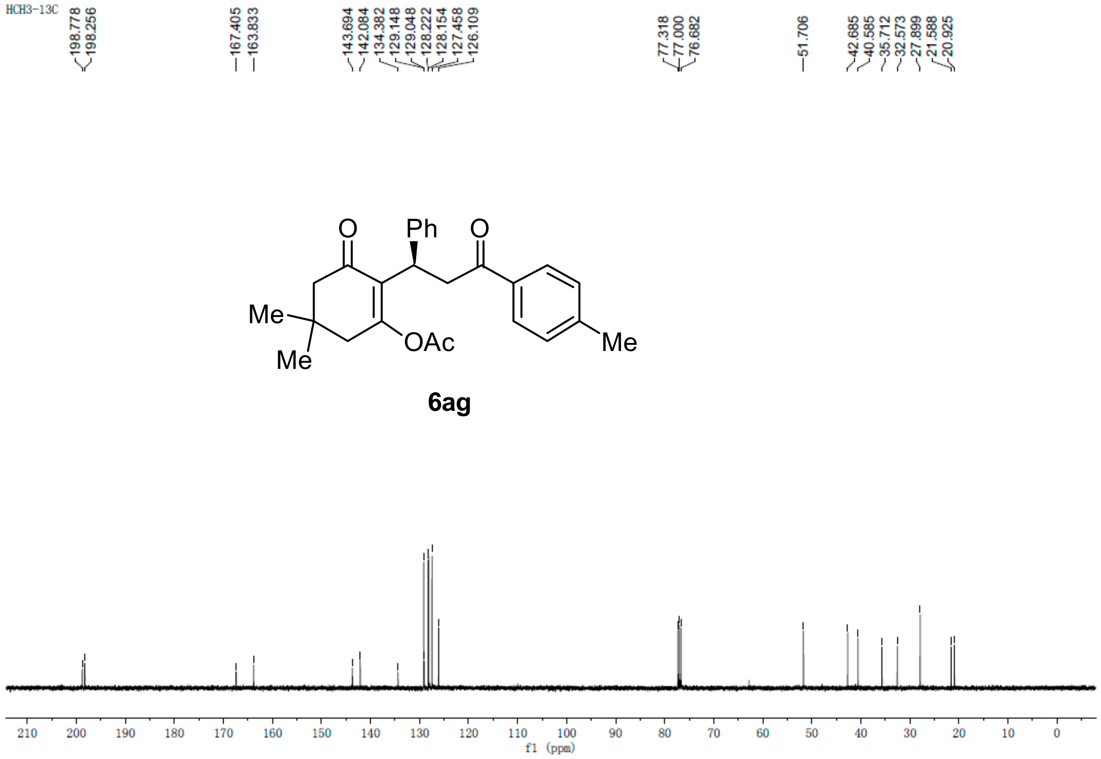
HCH3-13C
198.778
198.256
167.405
163.833
143.694
142.084
134.382
129.148
129.048
128.222
128.154
127.458
126.109
77.318
77.000
76.682
51.706
42.685
40.585
35.712
32.573
27.899
21.588
20.925
O
Ph
O
Me
Me
OAc
Me
6ag
f1 (ppm)

Chromatogram

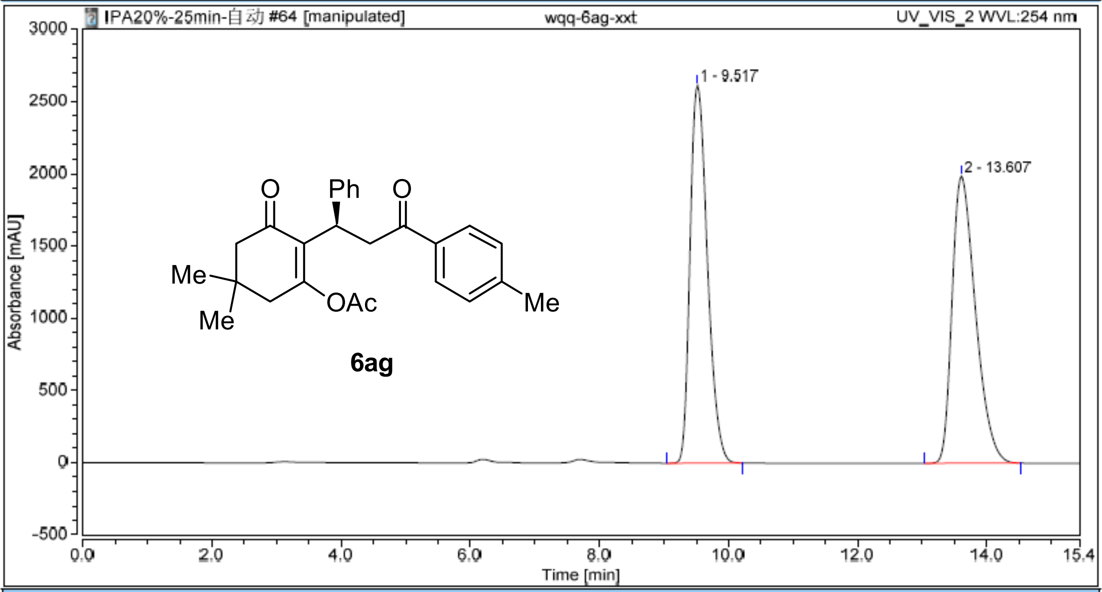

Integration Results

No.	Peak Name	Retention Time min	Area mAU*min	Height mAU	Relative Area %	Relative Height %	Amount n.a.
1		9.517	814.884	2615.938	49.34	56.83	n.a.
2		13.607	836.669	1987.432	50.66	43.17	n.a.
**Total:**			**1651.553**	**4603.370**	**100.00**	**100.00**	

Chromatogram

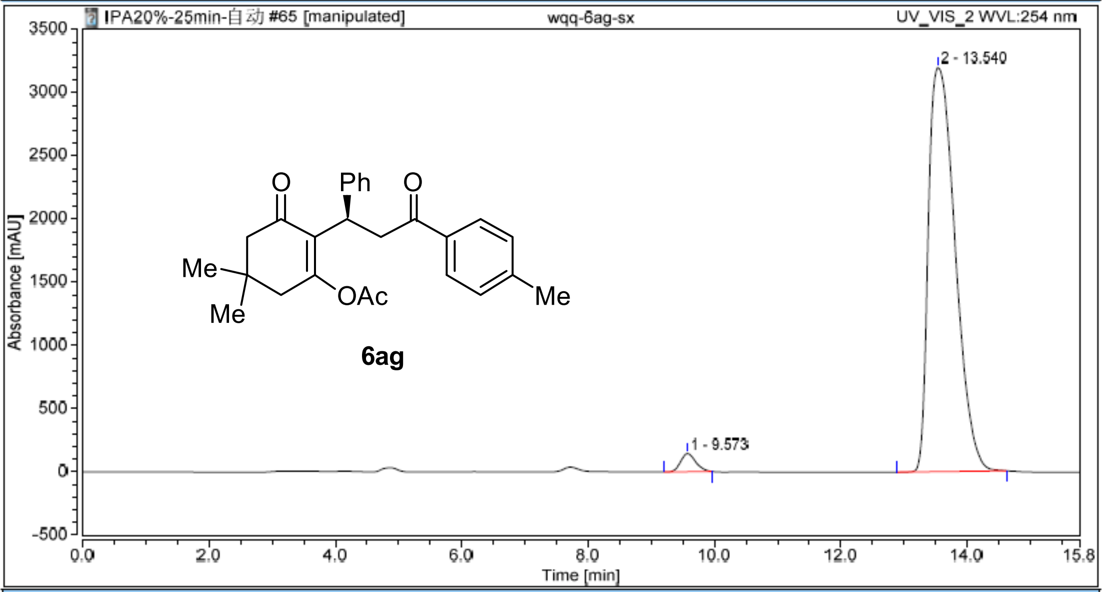

Integration Results

No.	Peak Name	Retention Time min	Area mAU*min	Height mAU	Relative Area %	Relative Height %	Amount n.a.
1		9.573	40.304	145.342	2.51	4.35	n.a.
2		13.540	1566.924	3196.046	97.49	95.65	n.a.
**Total:**			**1607.227**	**3341.388**	**100.00**	**100.00**	

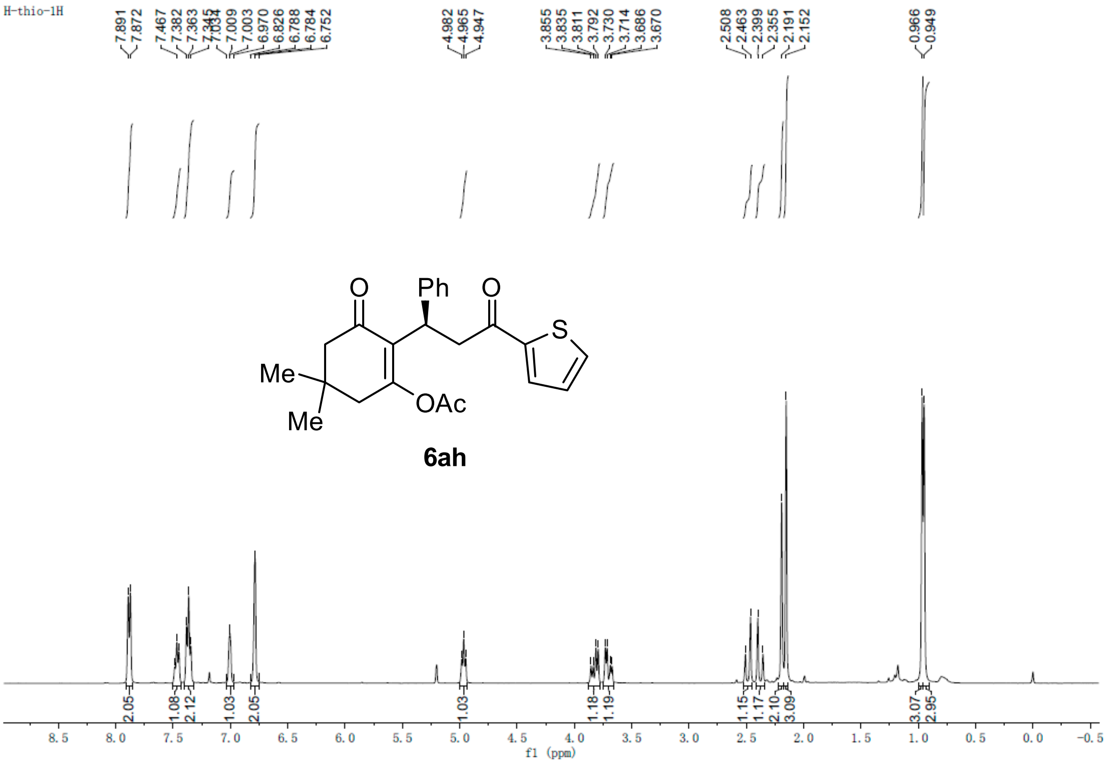
H-thio-1H
7.891
7.872
7.467
7.382
7.363
7.345
7.034
7.009
7.003
6.970
6.826
6.788
6.784
6.752
4.982
4.965
4.947
3.855
3.835
3.811
3.792
3.730
3.714
3.686
3.670
2.508
2.463
2.399
2.355
2.191
2.152
0.966
0.949
O
Ph
O
S
Me
OAc
Me
6ah
2.05
1.08
2.12
1.03
2.05
1.03
1.18
1.19
1.15
1.17
2.10
3.09
3.07
2.95
f1 (ppm)

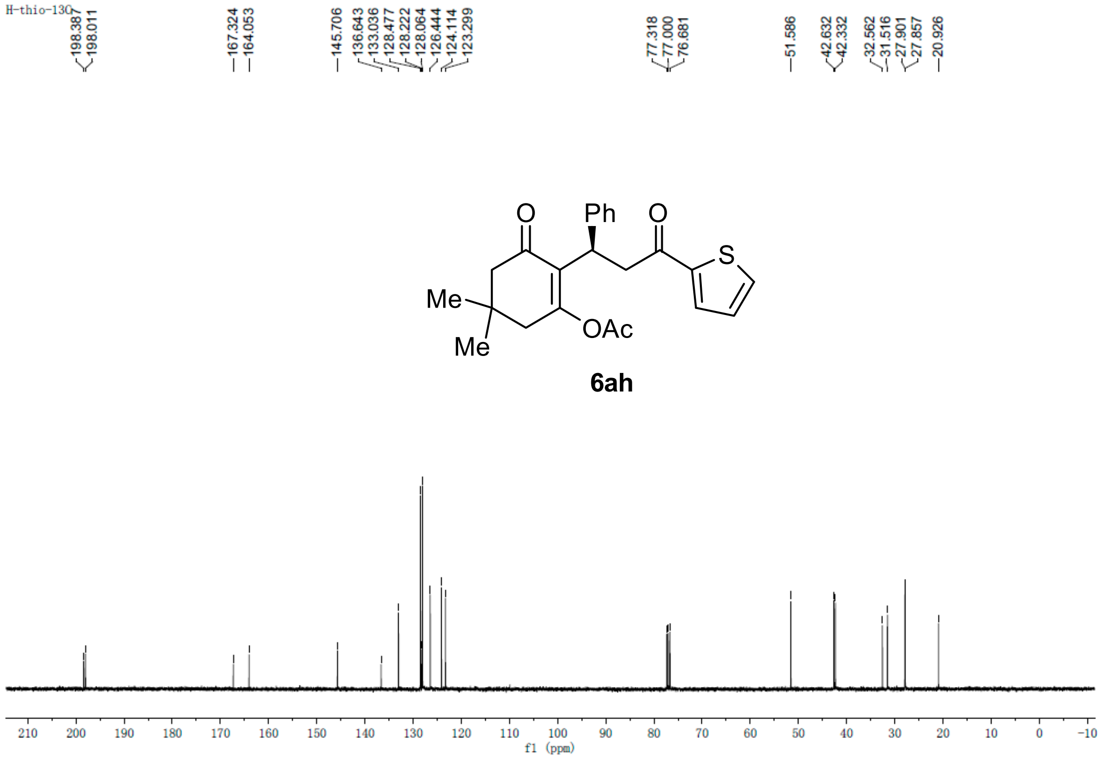
H-thio-13C
198.387
198.011
167.324
164.053
145.706
136.643
133.036
128.477
128.222
128.064
126.444
124.114
123.299
77.318
77.000
76.681
51.586
42.632
42.332
32.562
31.516
27.901
27.857
20.926
O
Ph
O
S
Me
OAc
Me
6ah
f1 (ppm)

Chromatogram

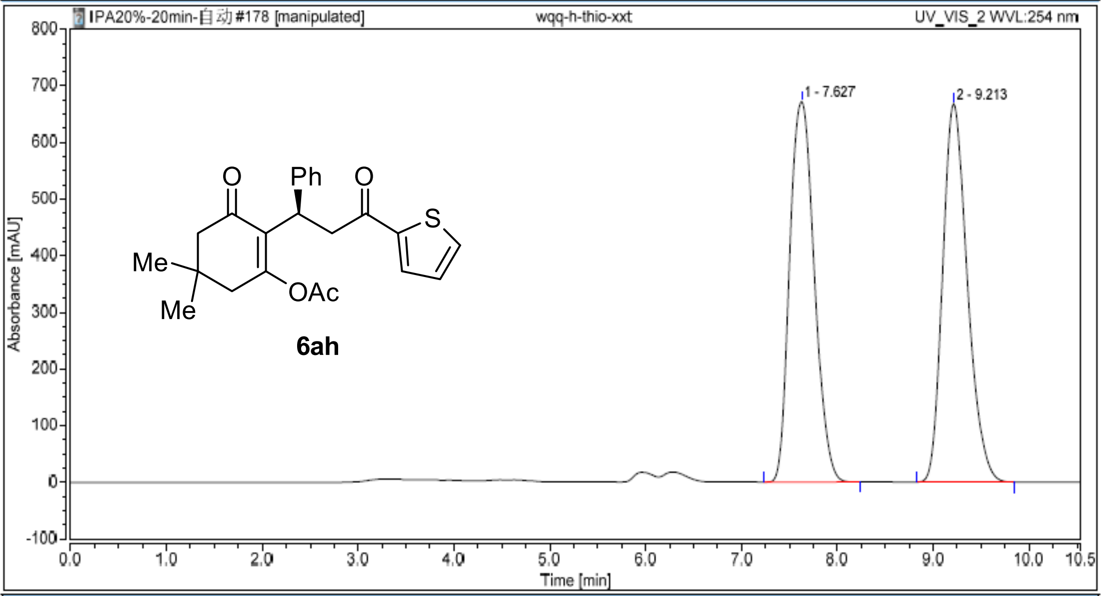

Integration Results

No.	Peak Name	Retention Time min	Area mAU*min	Height mAU	Relative Area %	Relative Height %	Amount n.a.
1		7.627	199.543	671.743	49.98	50.15	n.a.
2		9.213	199.728	667.727	50.02	49.85	n.a.
**Total:**			**399.270**	**1339.471**	**100.00**	**100.00**	

Chromatogram

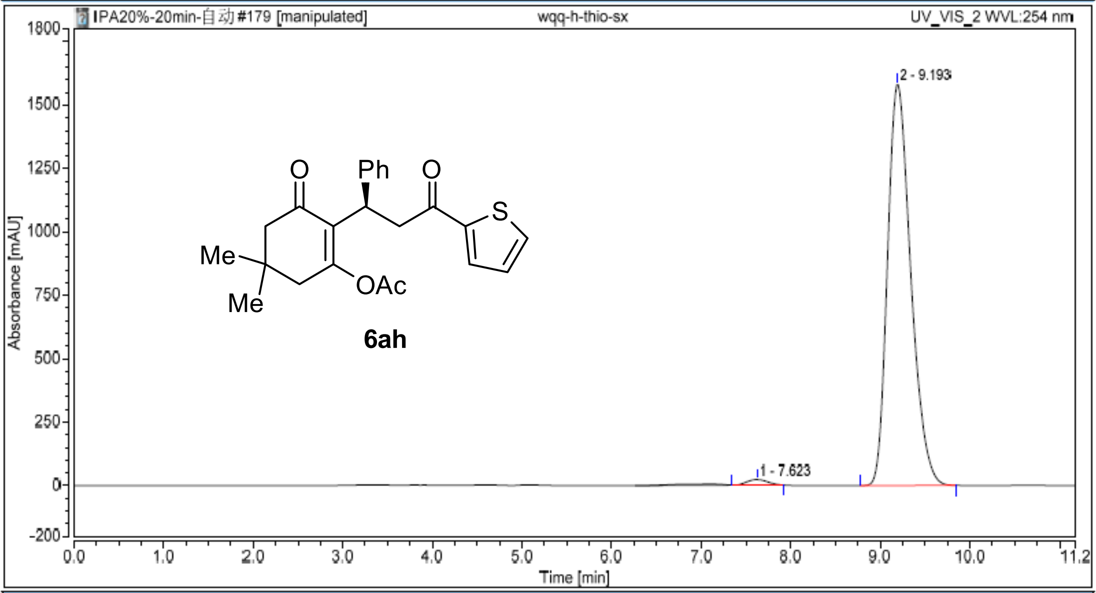

Integration Results

No.	Peak Name	Retention Time min	Area mAU*min	Height mAU	Relative Area %	Relative Height %	Amount n.a.
1		7.623	5.906	21.813	1.22	1.36	n.a.
2		9.193	476.388	1583.017	98.78	98.64	n.a.
**Total:**			**482.294**	**1604.830**	**100.00**	**100.00**	

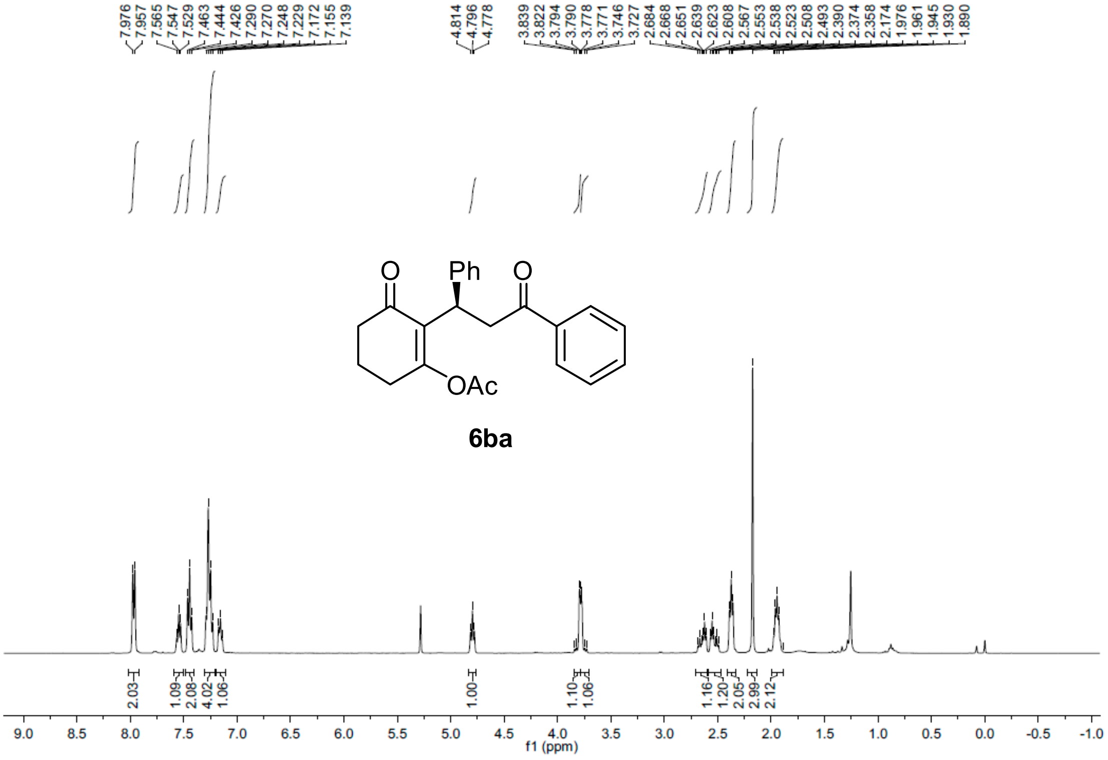

6ba

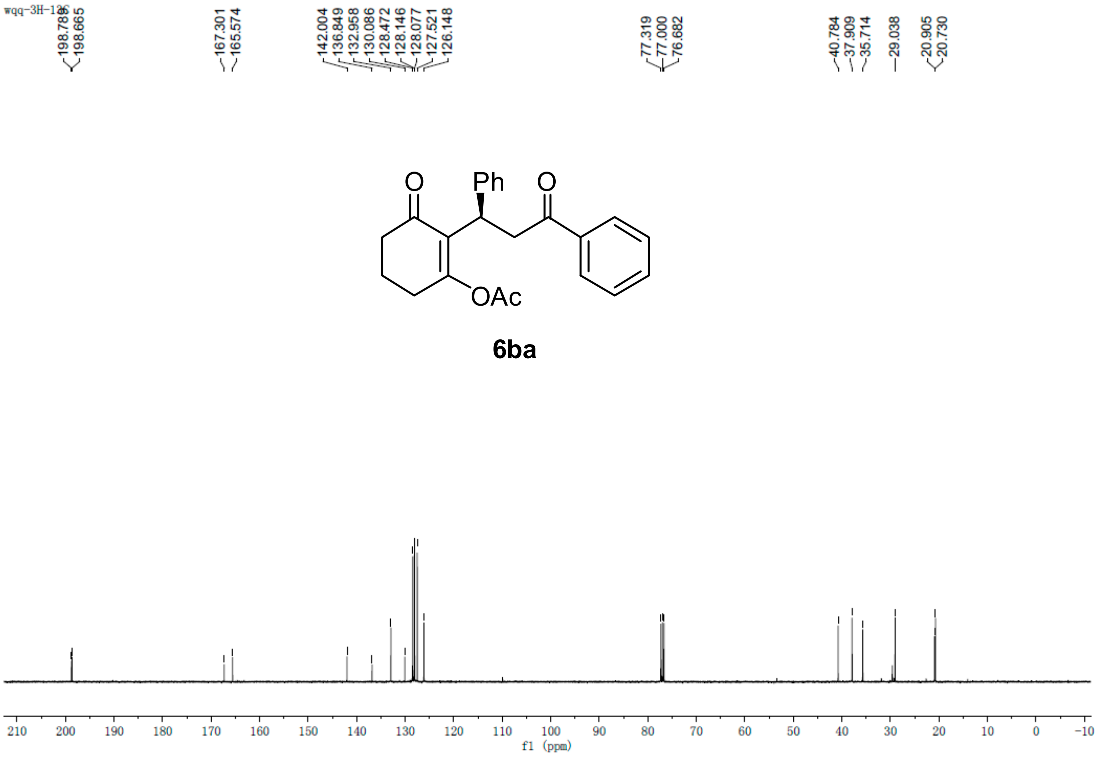

6ba

Chromatogram

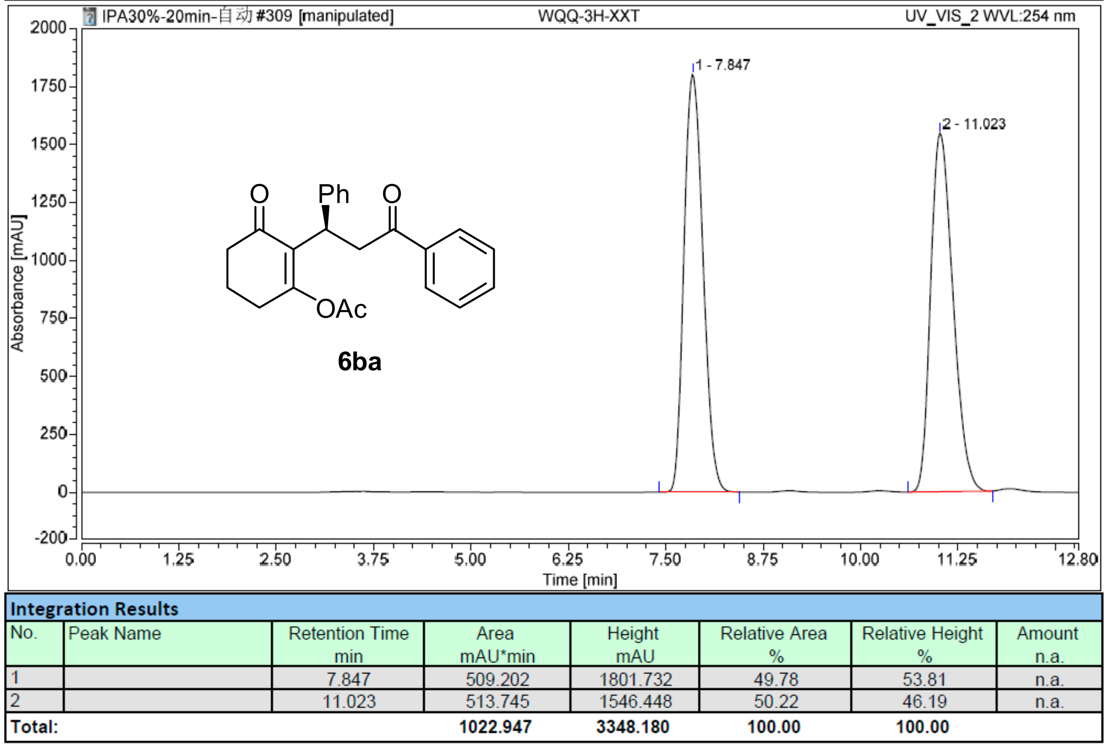

Integration Results

No.	Peak Name	Retention Time min	Area mAU*min	Height mAU	Relative Area %	Relative Height %	Amount n.a.
1		7.847	509.202	1801.732	49.78	53.81	n.a.
2		11.023	513.745	1546.448	50.22	46.19	n.a.
**Total:**			**1022.947**	**3348.180**	**100.00**	**100.00**	

Chromatogram

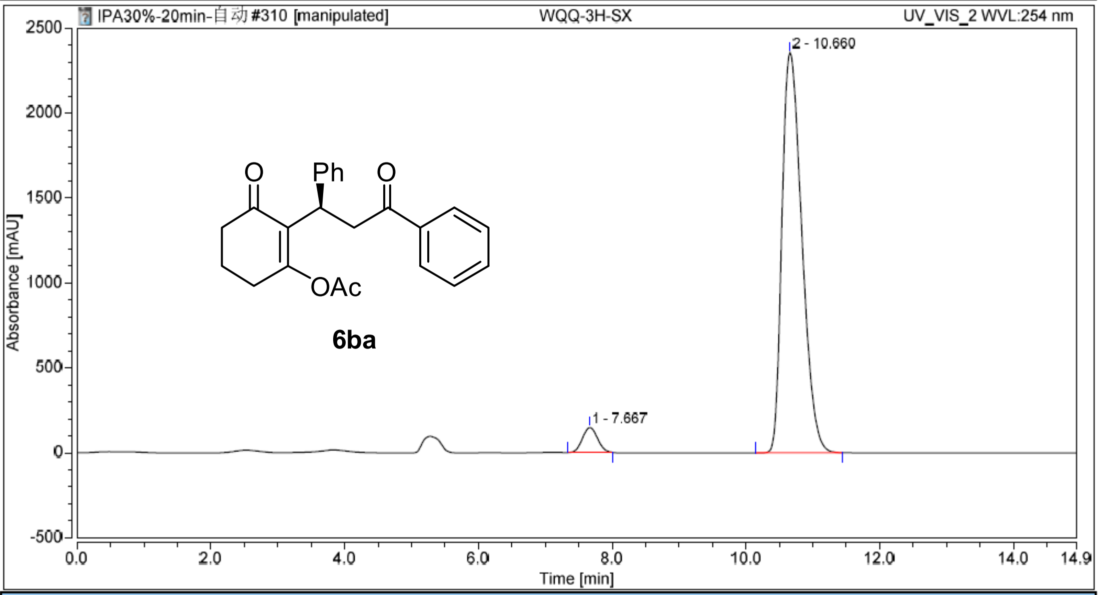

Integration Results

No.	Peak Name	Retention Time min	Area mAU*min	Height mAU	Relative Area %	Relative Height %	Amount n.a.
1		7.667	38.363	146.572	4.52	5.85	n.a.
2		10.660	810.356	2357.346	95.48	94.15	n.a.
**Total:**			**848.719**	**2503.919**	**100.00**	**100.00**	

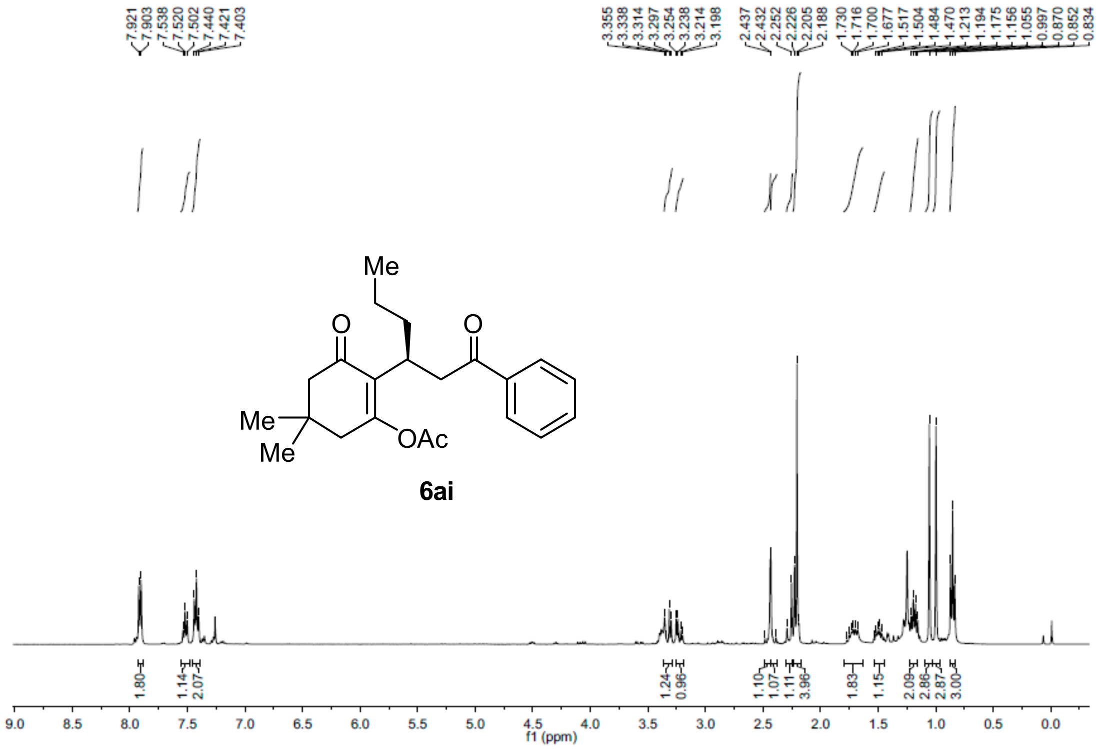
7.921
7.903
7.538
7.520
7.502
7.440
7.421
7.403
3.355
3.338
3.314
3.297
3.254
3.238
3.214
3.198
2.437
2.432
2.252
2.226
2.205
2.188
1.730
1.716
1.700
1.677
1.517
1.504
1.484
1.470
1.213
1.194
1.175
1.156
1.055
0.997
0.870
0.852
0.834
Me
O
O
Me
Me
OAc
6ai
1.80
1.14
2.07
1.24
0.96
1.10
1.07
1.11
3.96
1.83
1.15
2.09
2.86
2.87
3.00
f1 (ppm)

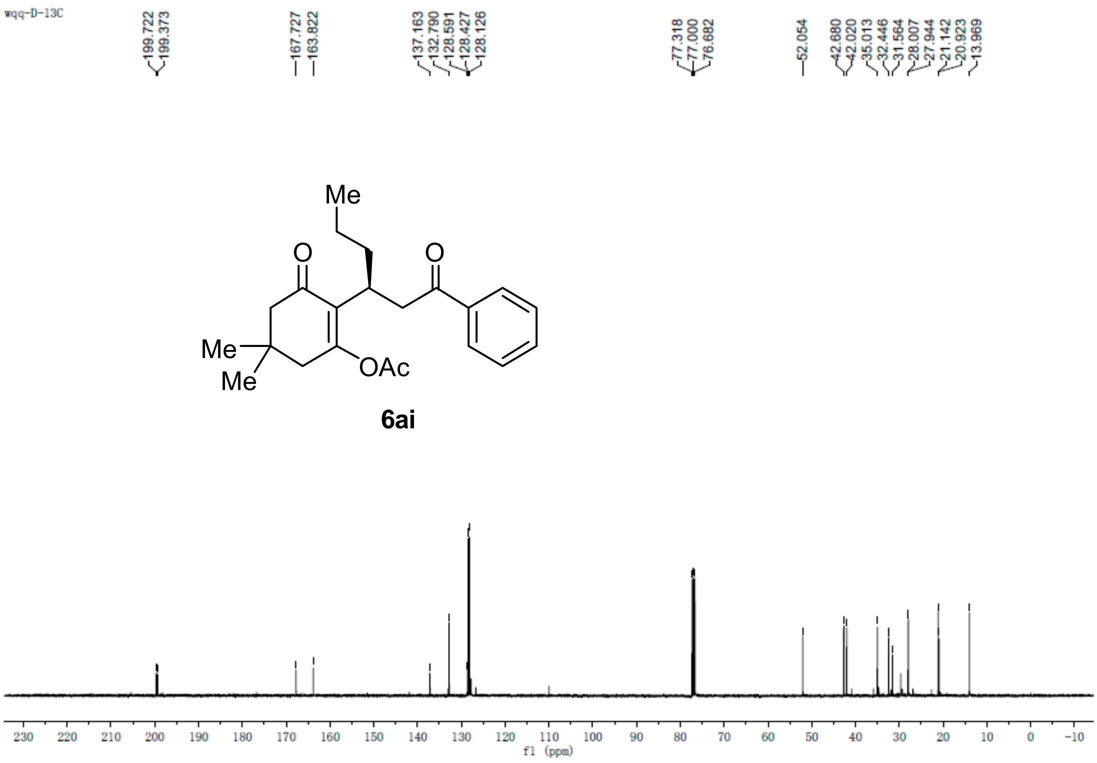
wqq-D-13C
199.722
199.373
167.727
163.822
137.163
132.790
128.591
128.427
128.126
77.318
77.000
76.682
52.054
42.680
42.020
35.013
32.446
31.564
28.007
27.944
21.142
20.923
13.969
Me
O
O
Me
Me
OAc
6ai
f1 (ppm)

Chromatogram

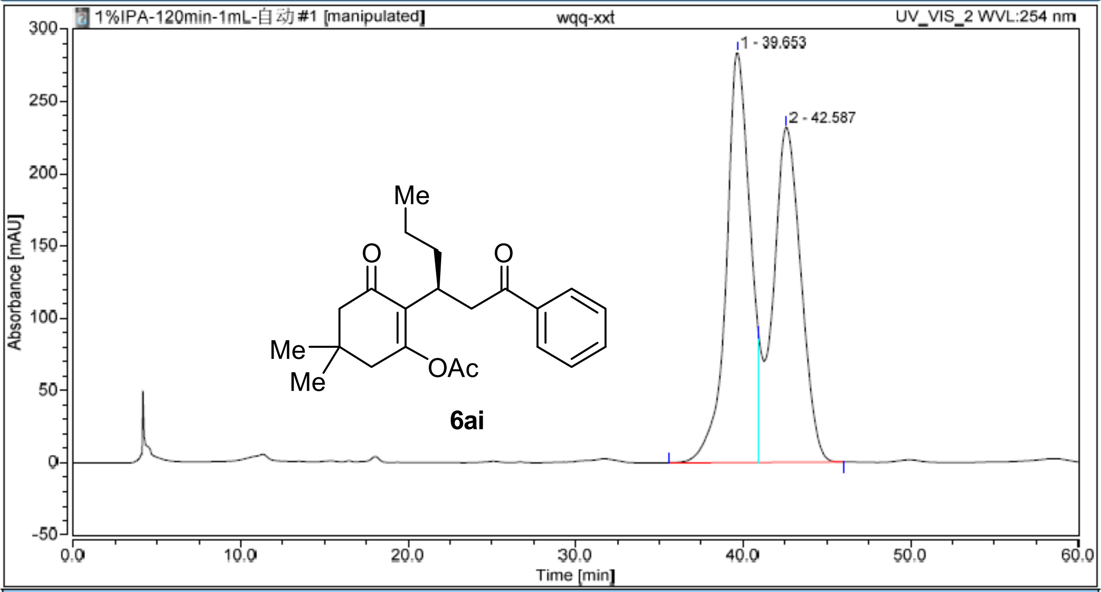

Integration Results

No.	Peak Name	Retention Time min	Area mAU*min	Height mAU	Relative Area %	Relative Height %	Amount n.a.
1		39.653	464.790	283.873	50.63	55.03	n.a.
2		42.587	453.190	231.983	49.37	44.97	n.a.
**Total:**			**917.979**	**515.856**	**100.00**	**100.00**	

Chromatogram

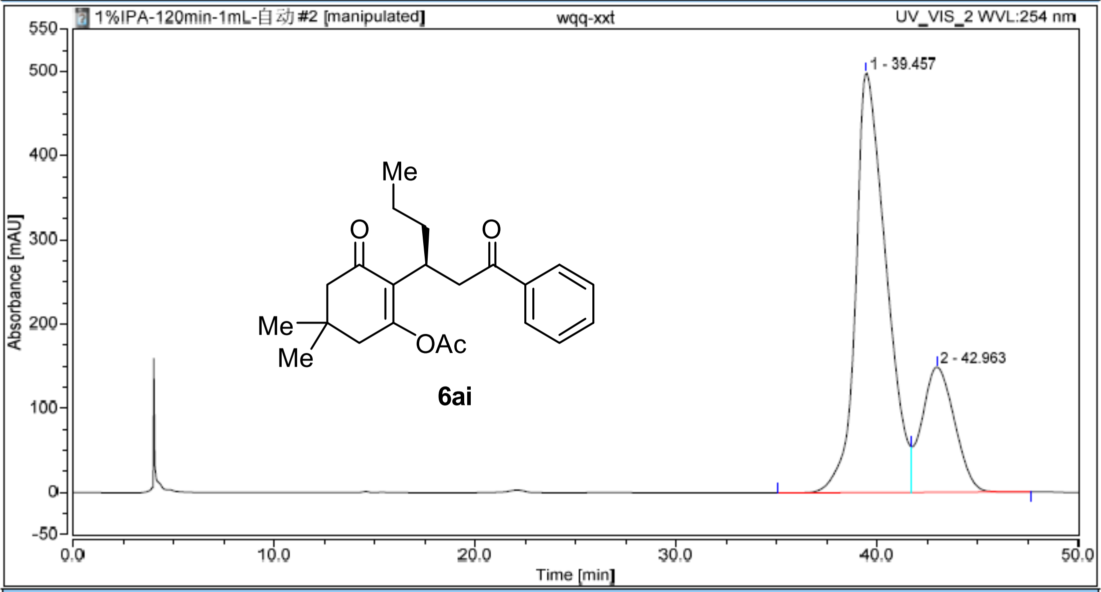

Integration Results

No.	Peak Name	Retention Time min	Area mAU*min	Height mAU	Relative Area %	Relative Height %	Amount n.a.
1		39.457	874.427	498.142	75.82	77.04	n.a.
2		42.963	278.934	148.459	24.18	22.96	n.a.
**Total:**			**1153.361**	**646.600**	**100.00**	**100.00**	

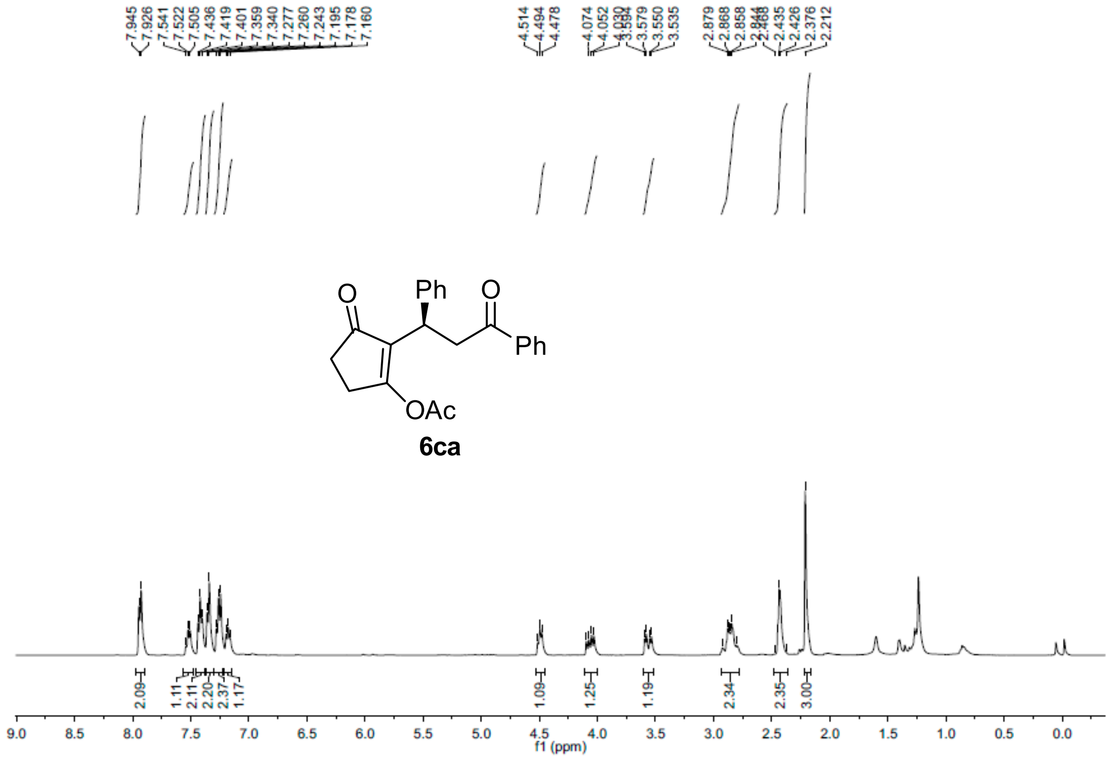
6ca
f1 (ppm)

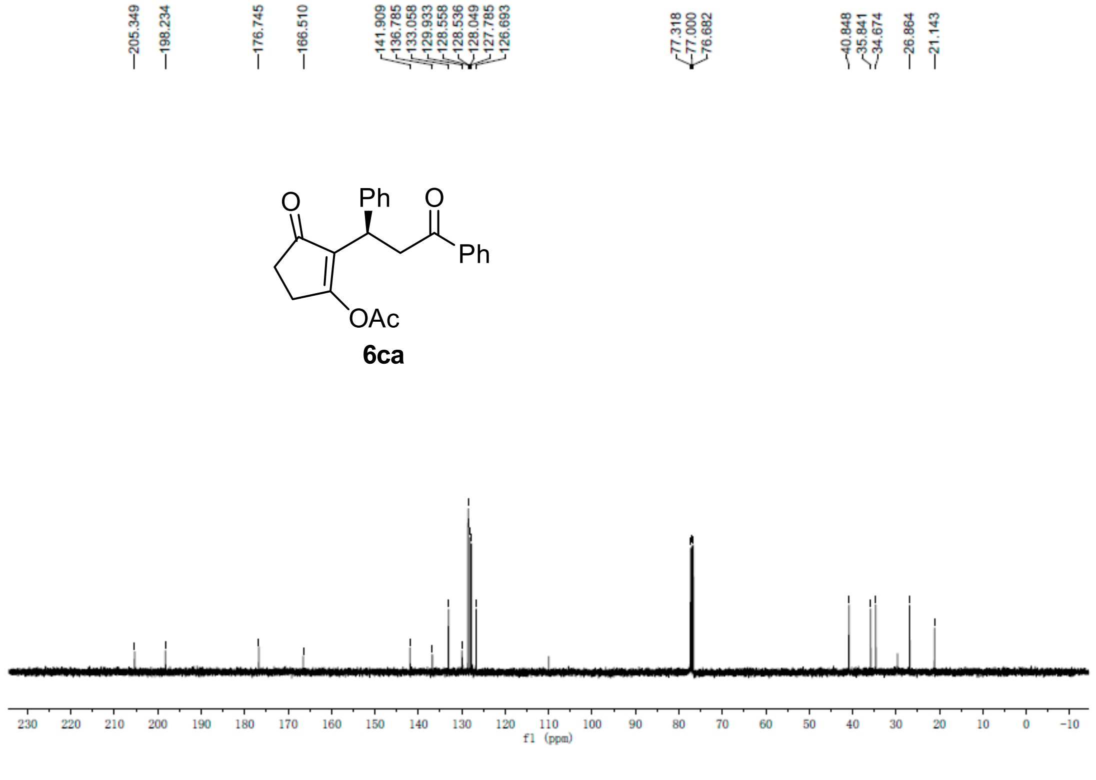
6ca
f1 (ppm)

Chromatogram

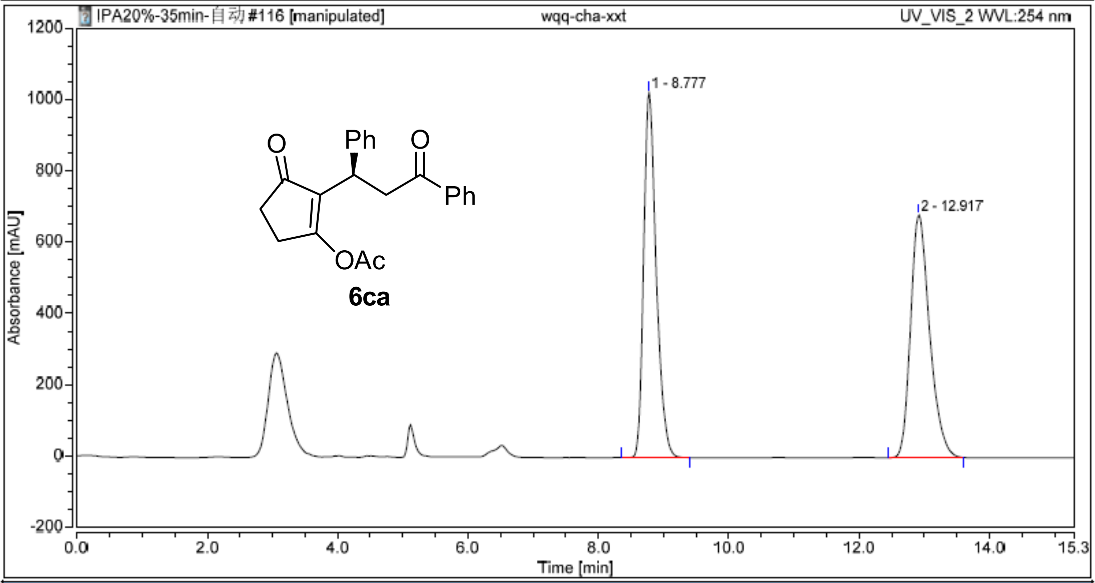

Integration Results

No.	Peak Name	Retention Time min	Area mAU*min	Height mAU	Relative Area %	Relative Height %	Amount n.a.
1		8.777	227.805	1023.131	49.68	60.06	n.a.
2		12.917	230.738	680.272	50.32	39.94	n.a.
Total:			**458.543**	**1703.403**	**100.00**	**100.00**	

Chromatogram

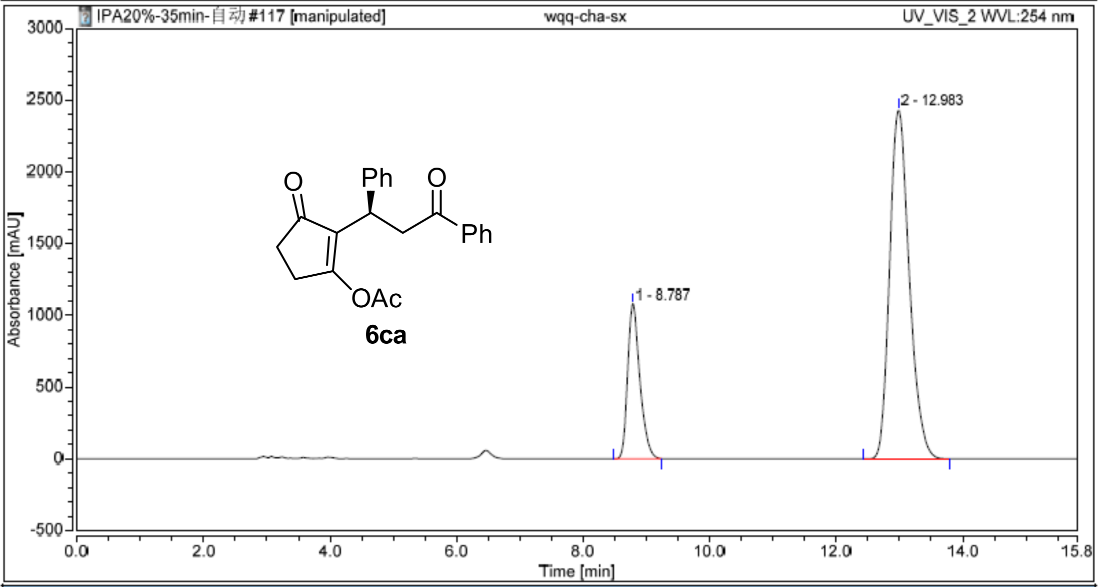

Integration Results

No.	Peak Name	Retention Time min	Area mAU*min	Height mAU	Relative Area %	Relative Height %	Amount n.a.
1		8.787	241.222	1079.742	21.60	30.74	n.a.
2		12.983	875.350	2432.648	78.40	69.26	n.a.
Total:			**1116.572**	**3512.390**	**100.00**	**100.00**	

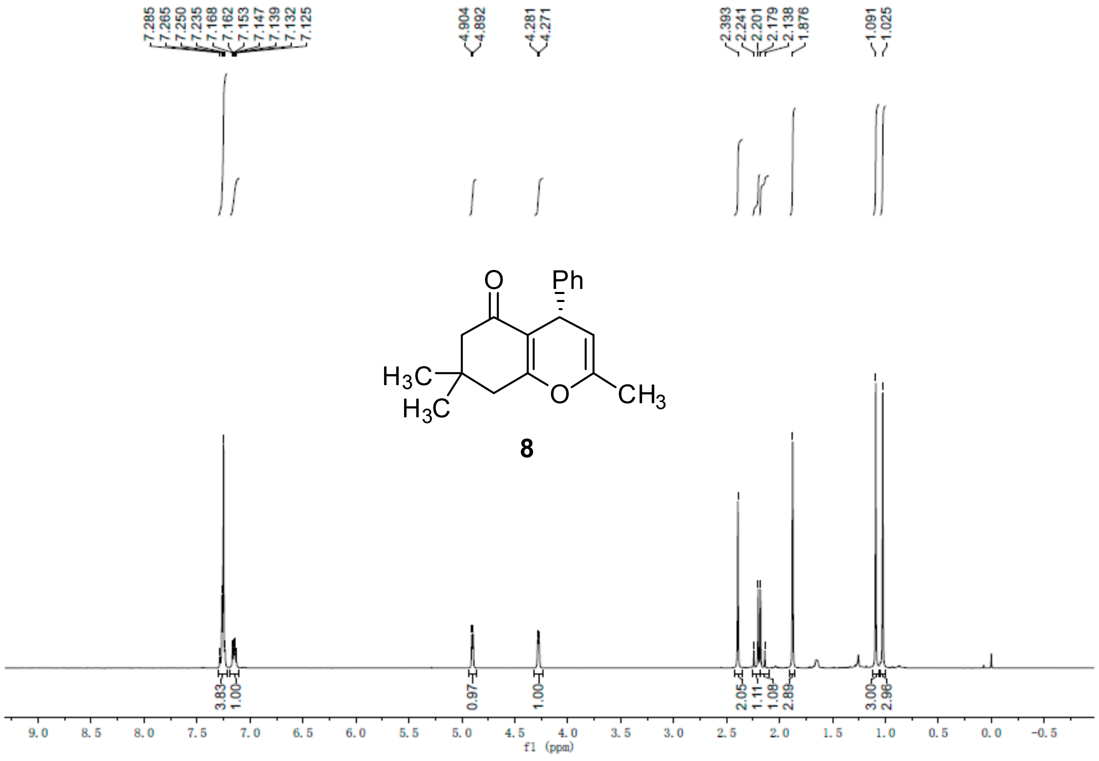
7.285
7.265
7.250
7.235
7.168
7.162
7.153
7.147
7.139
7.132
7.125
4.904
4.892
4.281
4.271
2.393
2.241
2.201
2.179
2.138
1.876
1.091
1.025
Ph
O
H3C
H3C
CH3
3.83
1.00
0.97
1.00
2.05
1.11
1.08
2.89
3.00
2.96
f1 (ppm)

8

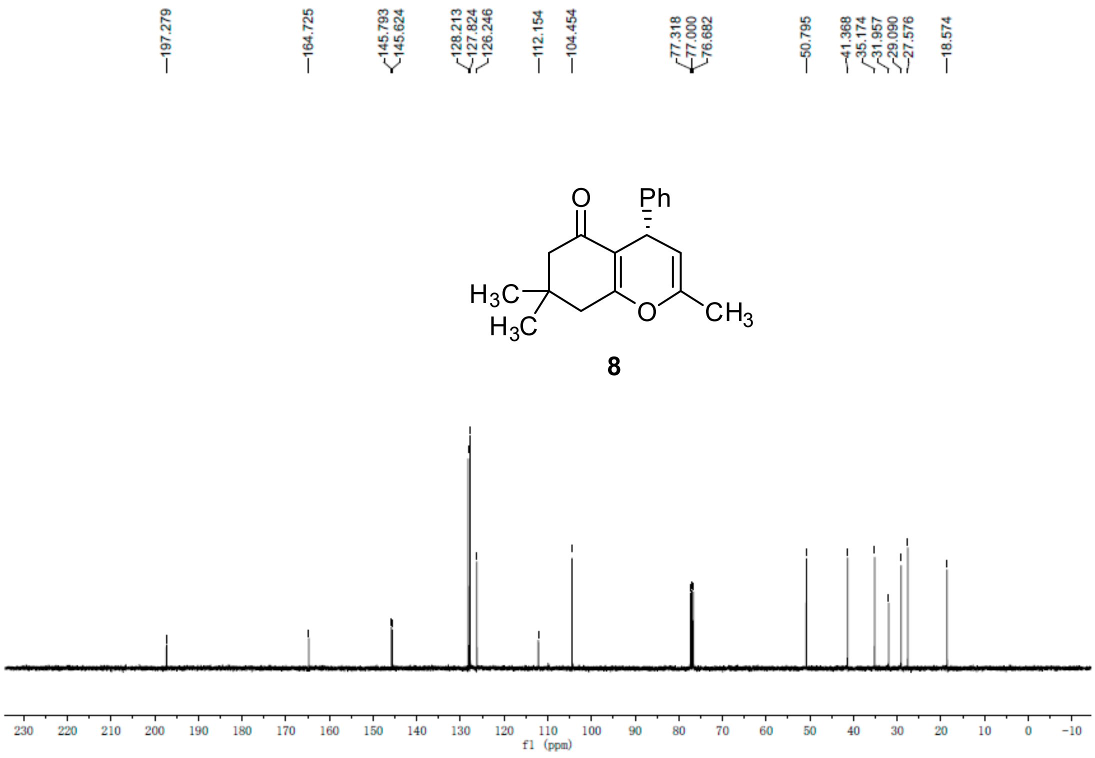
197.279
164.725
145.793
145.624
128.213
127.824
126.246
112.154
104.454
77.318
77.000
76.682
50.795
41.368
35.174
31.957
29.090
27.576
18.574
Ph
O
H3C
H3C
CH3
f1 (ppm)

8

Chromatogram

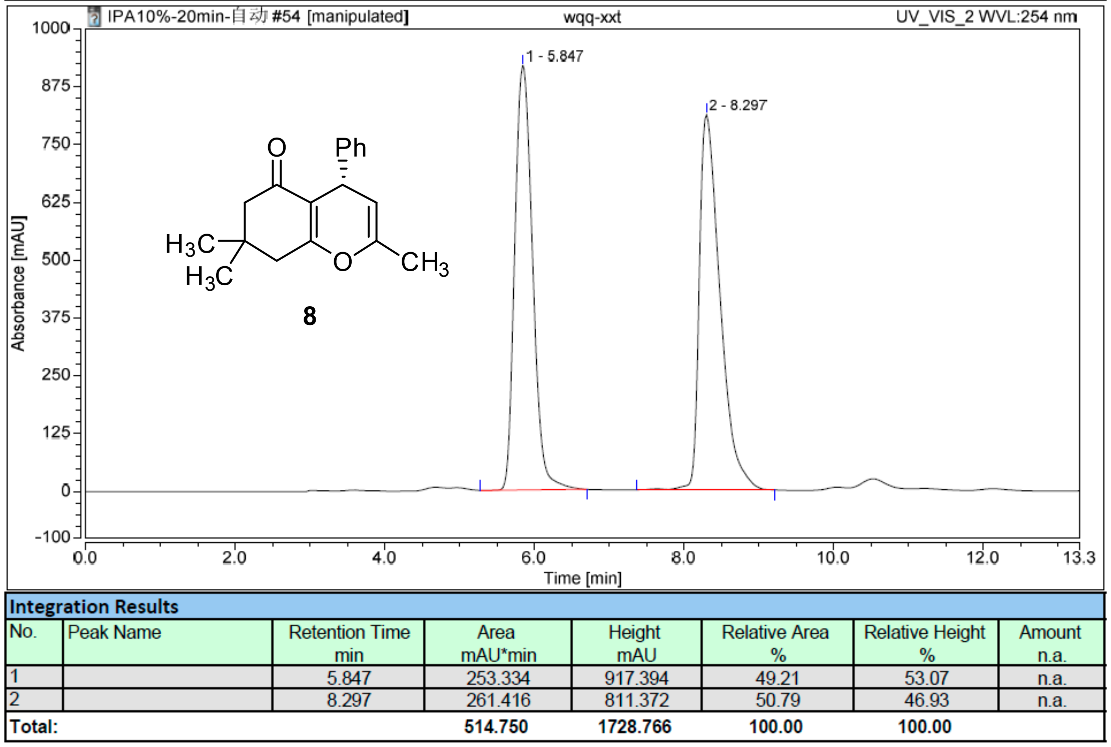

Integration Results

No.	Peak Name	Retention Time min	Area mAU*min	Height mAU	Relative Area %	Relative Height %	Amount n.a.
1		5.847	253.334	917.394	49.21	53.07	n.a.
2		8.297	261.416	811.372	50.79	46.93	n.a.
**Total:**			**514.750**	**1728.766**	**100.00**	**100.00**	

Chromatogram

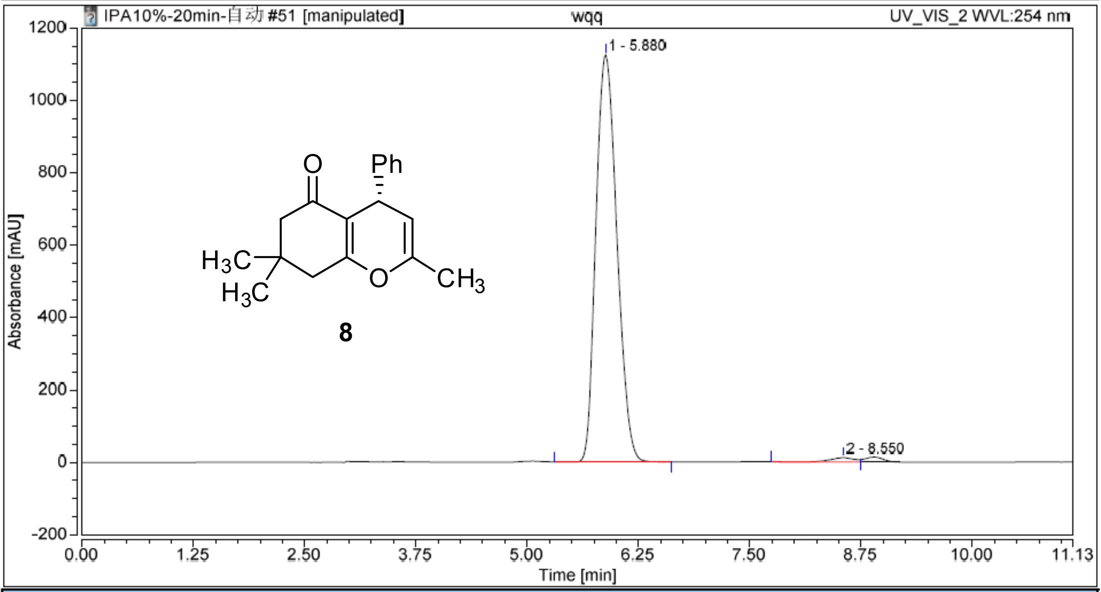

Integration Results

No.	Peak Name	Retention Time min	Area mAU*min	Height mAU	Relative Area %	Relative Height %	Amount n.a.
1		5.880	307.429	1125.455	98.89	98.98	n.a.
2		8.550	3.448	11.546	1.11	1.02	n.a.
**Total:**			**310.877**	**1137.002**	**100.00**	**100.00**	

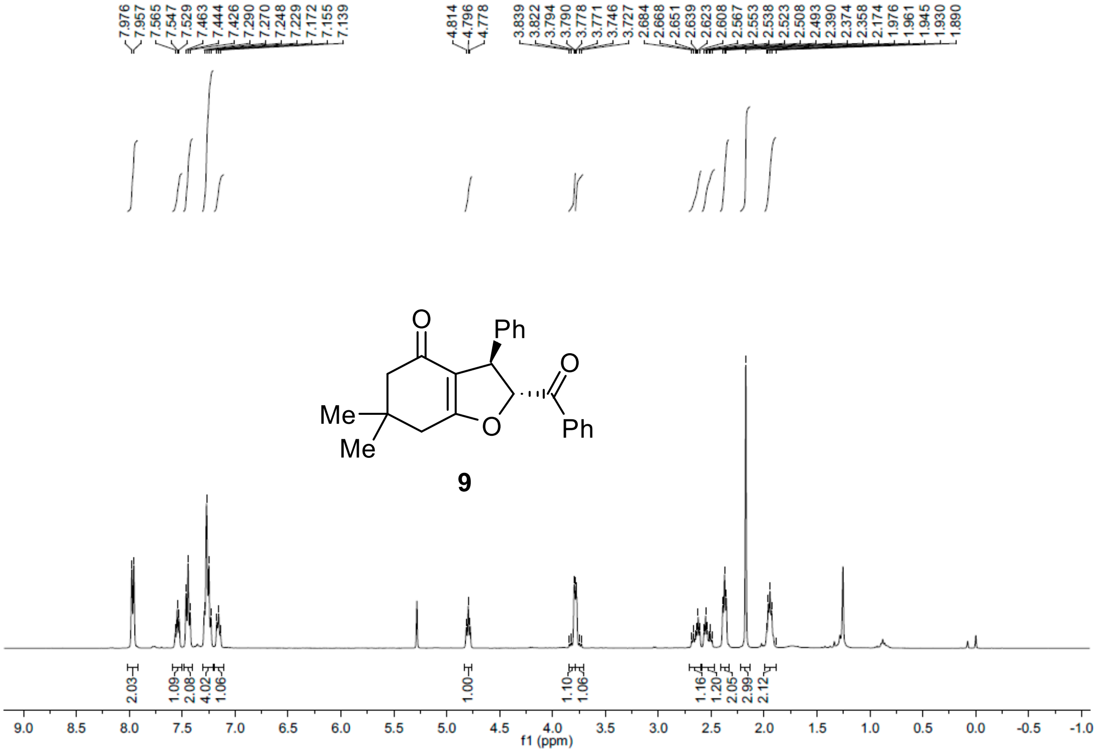

9

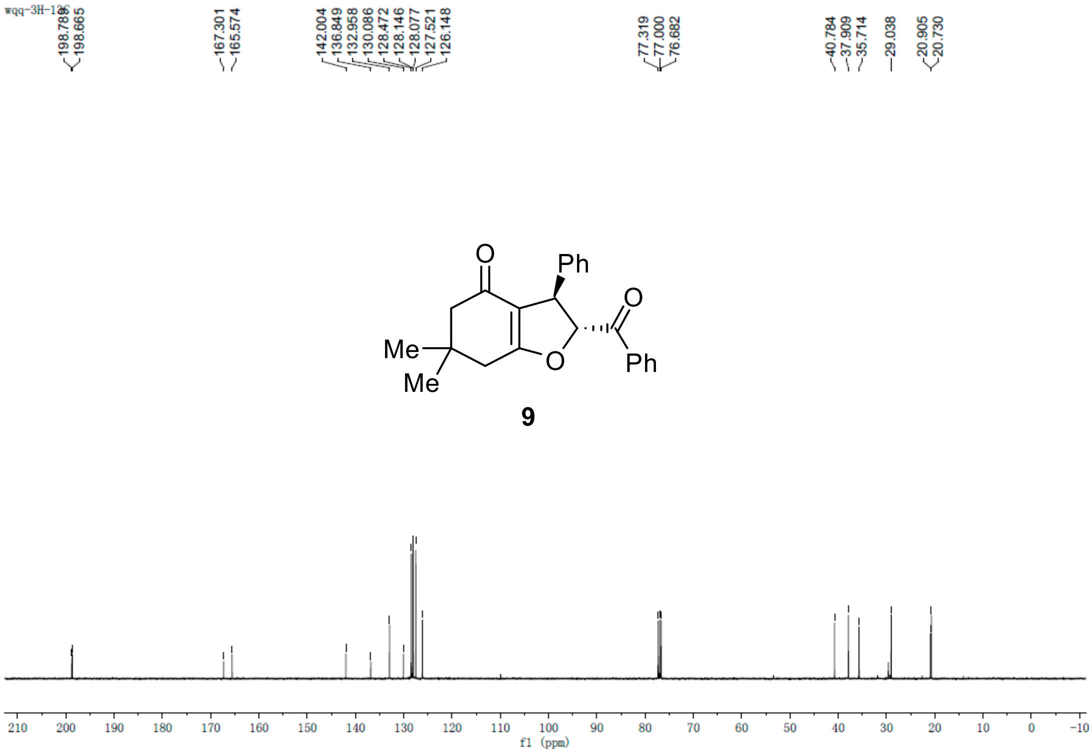

9

Chromatogram

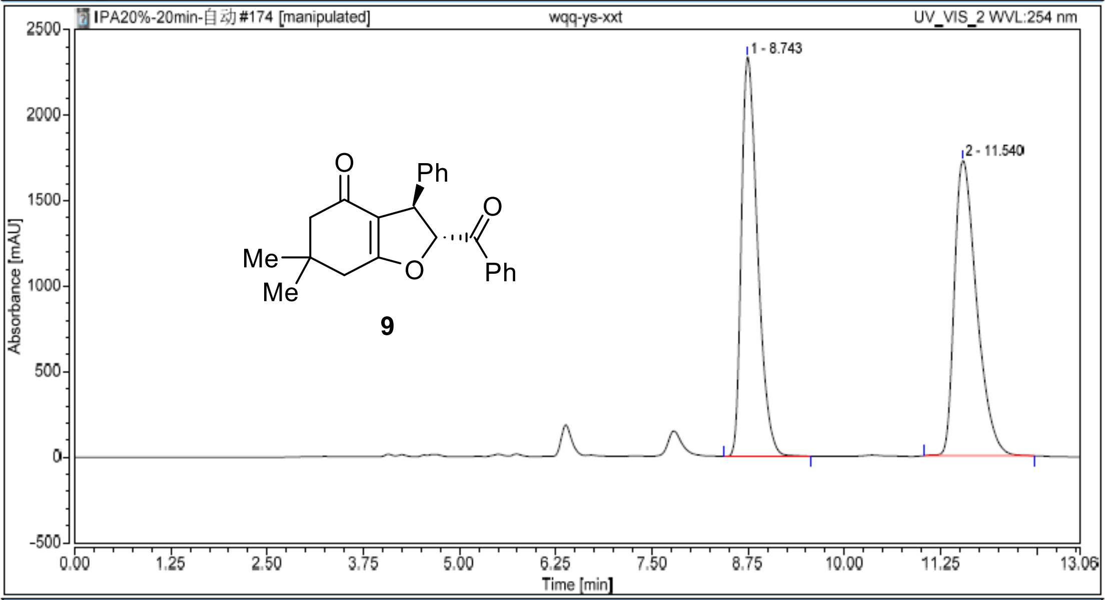

Integration Results

No.	Peak Name	Retention Time min	Area mAU*min	Height mAU	Relative Area %	Relative Height %	Amount n.a.
1		8.743	570.398	2332.910	49.65	57.49	n.a.
2		11.540	578.436	1725.124	50.35	42.51	n.a.
**Total:**			**1148.834**	**4058.034**	**100.00**	**100.00**	

Chromatogram

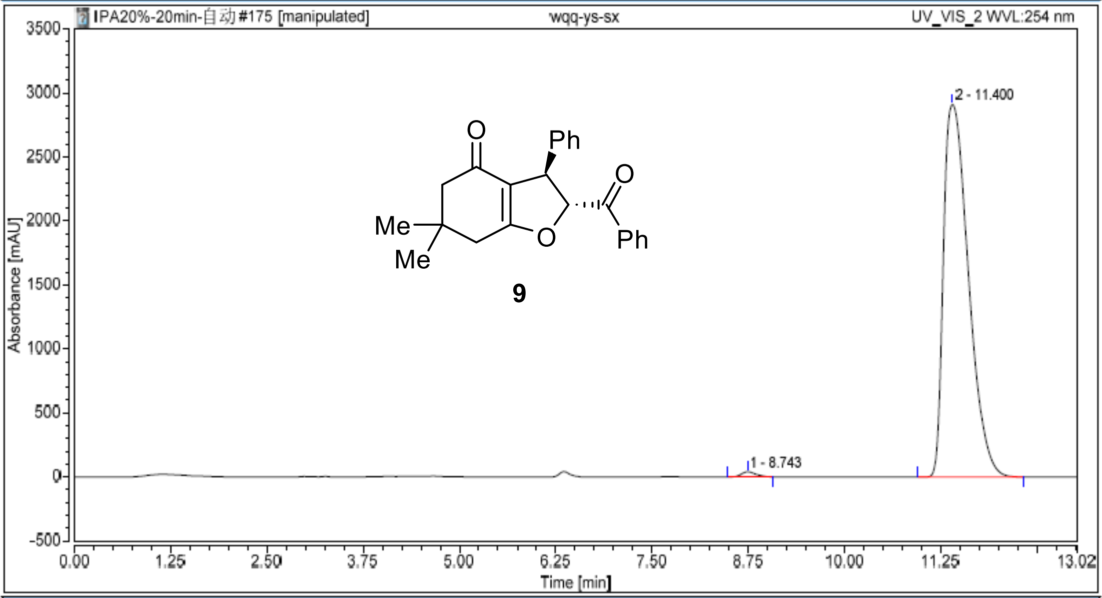

Integration Results

No.	Peak Name	Retention Time min	Area mAU*min	Height mAU	Relative Area %	Relative Height %	Amount n.a.
1		8.743	8.588	39.745	0.78	1.35	n.a.
2		11.400	1092.807	2910.217	99.22	98.65	n.a.
**Total:**			**1101.395**	**2949.963**	**100.00**	**100.00**	
